# The extraordinary osteology and functional morphology of the limbs in Palorchestidae, a family of strange extinct marsupial giants

**DOI:** 10.1371/journal.pone.0221824

**Published:** 2019-09-13

**Authors:** Hazel L. Richards, Rod T. Wells, Alistair R. Evans, Erich M. G. Fitzgerald, Justin W. Adams

**Affiliations:** 1 School of Biological Sciences, Monash University, Clayton, Victoria, Australia; 2 Geosciences, Museums Victoria, Melbourne, Victoria, Australia; 3 Ecology and Evolution, College of Science and Engineering, Flinders University, Adelaide, South Australia, Australia; 4 Palaeontology, South Australian Museum, Adelaide, South Australia, Australia; 5 Department of Anatomy & Developmental Biology, School of Biomedical Sciences, Faculty of Medicine, Nursing and Health Sciences, Monash University, Clayton, Victoria, Australia; Griffith University, AUSTRALIA

## Abstract

The Palorchestidae are a family of marsupial megafauna occurring across the eastern Australian continent from the late Oligocene through to their extinction in the Late Pleistocene. The group is known for their odd ‘tapir-like’ crania and distinctive clawed forelimbs, but their appendicular anatomy has never been formally described. We provide the first descriptions of the appendicular skeleton and body mass estimates for three palorchestid species, presenting newly-identified, and in some cases associated, material of mid-Miocene *Propalorchestes*, Plio-Pleistocene *Palorchestes parvus* and Pleistocene *Palorchestes azael* alongside detailed comparisons with extant and fossil vombatiform marsupials. We propose postcranial diagnostic characters at the family, genus and species level. Specialisation in the palorchestid appendicular skeleton evidently occurred much later than in the cranium and instead correlates with increasing body size within the lineage. We conclude that palorchestid forelimbs were highly specialised for the manipulation of their environment in the acquisition of browse, and that they may have adopted bipedal postures to feed. Our results indicate palorchestids were bigger than previously thought, with the largest species likely weighing over 1000 kg. Additionally, we show that *P*. *azael* exhibits some of the most unusual forelimb morphology of any mammal, with a uniquely fixed humeroulnar joint unlike any of their marsupial kin, living or extinct.

## Introduction

Australia’s marsupial megafauna became largely extinct during the Late Pleistocene, likely due to a combination of climatic change and human activity [1, 2]. The Vombatomorphia were an adaptively-diverse clade that once included carnivorous ambush predators (thylacoleonids), giant arboreal specialists (*Nimbadon* spp.), and the largest marsupial to have ever lived, *Diprotodon optatum* (see Table 1). Perhaps least understood among these vombatomorphs are the Palorchestidae. Palorchestids were a sister family to the better-known Diprotodontidae, whose closest living relatives are wombats (Vombatidae) and, more distantly, koalas (Phascolarctidae) [3–8]. These giant quadrupeds possessed a suite of morphological features totally unlike any living mammal, including a skull with hyper-retracted nasals, accompanied by powerful specialised forelimbs equipped with enormous scimitar-like claws. This led palaeontologists to speculate about ecological similarities between palorchestids and other large extinct clawed mammals like ground sloths and chalicotheres [9, 10], an ecomorph notably absent from modern faunas [11]. Despite an assortment of postcranial material identified in Australian museum collections since the 1970s [9, 10], historical emphasis towards craniodental research has meant that even basic descriptions of the palorchestid appendicular skeleton have yet to be published.

**Table 1:** Relationships, occurrences, appendicular osteology sources and previously published mass estimates for comparative vombatiform taxa.

		Taxonomy				Stratigraphic range	Appendicular osteology	Published mass estimate (kg)
Vombatiformes								
	Phascolarctomorphia							
		Phascolarctidae		*Phascolarctos*	*cinereus*	Quaternary	Lee and Carrick [12]	12[13]
	Vombatomorphia							
		Thylacoleonidae^†^		*Thylacoleo*	*carnifex*	Plio-Pleistocene	Finch and Freedman [14]	87^[^^15^^,^ ^16^^]^
		Vombatidae		*Phascolonus*^†^	*gigas*	Pleistocene	Stirling [17]	250^[^^6^^,^ ^16^^]^
				*Lasiorhinus*	*latifrons*	Quaternary	Scott and Richardson [18]	25^[^^13^^]^
				*Vombatus*	*ursinus*	Quaternary	Owen [19]	26^[^^13^^]^
		Diprotodontoidea^†^						
		Diprotodontidae^†^							
			Zygomaturinae	*Nimbadon*	*lavarackorum*	Miocene	Black et al. [20]	70^[^^16^^,^ ^20^^]^
				*Neohelos*	*stirtoni*	Miocene	Murray et al. [21]	250^[^^16^^,^ ^22^^]^
				*Zygomaturus*	*trilobus*	Pleistocene	Scott [23]	675^[^^16^^,^ ^22^^]^
			Diprotodontinae	*Ngapakaldia*	*tedfordi*	Oligo-Miocene	Munson [24]	‘sheep sized’ ^[^^25^^]*^
					*bonythoni*	Oligo-Miocene	Munson [24]	‘sheep sized’^[^^25^^]*^
				*Diprotodon*	*optatum*	Pleistocene	Owen [26]	2786^[^^27^^]^
		**Palorchestidae**^†^						
				***Propalorchestes***	**sp.**	Oligo-Miocene	This study	‘lamb sized’^[^^28^^]*^
				***Palorchestes***	***parvus***	Plio-Pleistocene	This study	100^[^^29^^]*^
					***azael***	Pleistocene	This study	500^[^^29^^]*^

† indicates extinct taxa. Taxonomy and occurrences adapted from Murray [6], Black [7] and Black et al. [5] (see [8] for an alternative position for Thylacoleonidae).

Mass estimation method used by these authors was that of Anderson et al. [16] using stylopodial circumferences, and are means except where taxa are extant, or * where no method was reported.

Here we present the first formal descriptions of palorchestid appendicular morphology, highlighting morphological trends within the lineage over evolutionary time and proposing diagnostic characters at the family, genus and species level where possible. We also provide the first quantitative body mass estimates for the group. This work represents an important first step toward further taxonomic, biomechanical and palaeoecological analyses of these unusual marsupials.

### Historic and current understanding of palorchestids

Palorchestids have been recorded at sites across eastern mainland Australia and Tasmania, and as a group occurred from the late Oligocene though to the major megafaunal extinction in the Late Pleistocene [7, 25, 30, 31]. Their narrow face, scooped incisors and bilophodont molar morphology suggest a selective browser diet, supported by dental microwear analysis of a single *P*. *azael* molar [32], while a specialised bark-feeding niche has also been proposed [10]. Palorchestid body mass has been roughly approximated as ‘lamb-sized’ in the earliest *Propalorchestes* species [28], 100 kg in the Plio-Pleistocene *P*. *parvus*, and 500 kg in the largest, most abundant and latest-surviving *Palorchestes azael* [29] (Table 1).

Despite being known principally from craniodental material, speculative reconstructions of palorchestid body form abound in the popular literature (reviewed in [33]). The form of such reconstructions has varied widely in the 145 years since the initial description of the type species *Palorchestes azael*. Owen’s [34] original misinterpretation of the macropod-like midlinks between molar lophs in the fragmentary holotype (NHMUK PV OR 46316) led to assumptions of a kangaroo-like body [35]. Eventual taxonomic reassessment by Woods [3], noting the lack of a diagnostic macropodine masseteric canal, placed them within the Diprotodontoidea, which suggested a more vombatomorph body shape. More recent reconstructions often resemble a ‘marsupial tapir’ largely due to the elongate palorchestid snout and autapomorphic retracted nasal bones bearing a superficial resemblance to those of tapirs [25]. Recent discovery of new fossils and subsequent re-examination of all known palorchestid cranial material have challenged this longstanding tapir analogy, omitting the hypothesised proboscis in favour of a sensitive prehensile lip accompanied by a long protrusible tongue [36]. Whatever its fleshy appearance, this unique rostral morphology was already evident in the earliest-known palorchestid crania, indicating early cranial specialisation at some point after their divergence from the Diprotodontidae [28, 37].

Palorchestid fossil finds are exceptionally rare, but have occurred over a large geographic range of what would have been forested environments [38, 39]. As a result, some have speculated they may have been solitary animals with slow reproductive rates and large home ranges [10, 40]. This can be contrasted with the fossils of other large diprotodontoids, such as *Nimbadon* [41] and *Diprotodon* [42], which are often recovered in groups of many individuals of various ontogenetic stages. Several sympatric palorchestid species occur at multiple localities and time periods across eastern Australian deposits [30, 39, 40, 43], and recently named additional species show that the evolution within the lineage was more complex than previously thought [38, 44–46].

The only palorchestid postcrania so far described as such in the peer-reviewed literature are a series of six caudal vertebrae assigned to P. azael [47], indicating the likelihood of a well-developed tail in that species. From later excavations at Victoria Fossil Cave, Wells [9] reported finding huge laterally-compressed ungual phalanges in association with some limb material distinct from those of other megafauna. These were tentatively assigned to Palorchestes but never formally described. In the popular book Kadimakara, Flannery and Archer [10] recount their recognition of an unidentified partial skeleton in the collection at Museums Victoria as P. azael based on diagnostic associated molars. Although this specimen (NMV P157144) was in poor condition and lacked locality data, its distinctive limb elements enabled them to identify another set of postcrania from Buchan, Victoria (NMV P159792) due to key morphological overlap between the two. This then led to recognition of a further palorchestid specimen recovered from Wee Jasper, New South Wales (Australian Museum AM F58870), which was subsequently assigned to P. parvus on the basis of its smaller size than the Victorian specimens, and association with a diagnostic premaxilla fragment. Although Flannery and Archer’s chapter provides a tantalising glimpse of the unique morphology of these limb bones, no photographs or formal descriptions have been presented.

### Geological settings

Table 2 provides an overview of the localities and geological contexts in which the main associated material described here was found.

**Table 2:** Geological settings for the main associated palorchestid material described.

Locality	Locality description	Formation	Age	Taxa
Buchan Caves, Victoria	Buchan Caves Reserve, East Gippsland, 370 km east of Melbourne	Caves are formed within Early Devonian Buchan Group limestone [48]. Pleistocene deposits found both within soft *terra rossa* sediments lining the cave floor and embedded in calcite flowstone of the walls and ceiling [49]	Uranium-thorium dating of calcite flowstone surrounding a *Palorchestes azael* skull yielded a date of 275 ka [49]	*Palorchestes azael* *P*. *parvus*
Keilor, Victoria	Confluence of Maribyrnong River and Dry Creek, approximately 16 km northwest of Melbourne	D Clay fossil bearing unit of the Arundel Formation, composed of fine-grained overbank silts [50, 51]	The 40–25 ka date range estimated by Joyce and Anderson [52] for the D Clay was questioned by Duncan [51] due to likely contamination of samples. They are currently assumed to be older than 31 ka	*P*. *azael*
Wee Jasper, New South Wales	Either Punchbowl or Signature cave in the Wee Jasper system, approximately 40 km north west of Canberra	Cave within Early Late Devonian Taemas Limestone formation, consisting of fossiliferous interbedded limestone and calcareous mudstone [53]	Sediments determined through magnetostratigraphy to date between 2.0 and 0.73 Ma [54]. One megafaunal fossil was dated to at least 45–30 ka via U-series analysis [55]	*P*. *parvus*
Bullock Creek, Northern Territory	In a river valley situated on Camfield Station, approximately 500 km south of Darwin	Camfield Limestone Beds freshwater carbonate unit	Biocorrelation and mammalian stage-of-evolution comparisons constrain the site to Mid-Miocene, between 16–13 Ma [22, 31, 55–57]	*Propalorchestes* sp.

## Methods

### Descriptions and comparisons

Here we collate and describe the appendicular material for *Palorchestes azael*, *P*. *parvus* and *Propalorchestes* accessible across five Australian museum collections (with one specimen from Natural History Museum, London). We figure the best-preserved examples of the representative morphology throughout. Over the course of this research additional material has been recognised in collections and referred where possible to the species level.

*Palorchestes azael*, the type species and that with the most known material, is first described in detail relative to other vombatiform marsupials. *Palorchestes parvus* and *Propalorchestes* are then described in terms of key differences to the type species.

Where possible, apomorphic diagnostic features at the family, genus and species level are proposed for palorchestid species, although full character state and phylogenetic analyses are beyond the scope of the present study. These emended diagnoses are aggregated at the beginning of the Systematic Palaeontology section to maximise accessibility of this information for the reader.

Throughout the text, comparisons are made with other vombatiform marsupials referred to in-text by generic names except for *Ngapakaldia*, where two species are referred to as *Ng*. *bonythoni* and *Ng*. *tedfordi*. The specific comparative taxa used and their relationships to palorchestids are detailed in Table 1 along with literature sources for their own appendicular osteological descriptions. Museum registration details for comparative vombatiform specimens are provided in S1 Table, and key limb elements from these comparative taxa are illustrated in S1–S3 Figs.

Select measurements are given in-text, with comprehensive measurements of all palorchestid specimens also available in S1 Table. Measurements < 150 mm were taken with digital callipers to the nearest tenth of a millimetre. Measurements > 150 mm were taken with vinyl tape to the nearest millimetre.

Osteological, myological and directional terminology follow *Illustrated Veterinary Anatomical Nomenclature* [58] except when inappropriate for marsupials, where nomenclature follows recent descriptive work by Harvey and Warburton [59], Warburton et al. [60] and Warburton and Marchal [61].

### Body mass estimation

The body masses of many extinct vombatiform taxa have previously been estimated using an equation derived by Anderson et al. [16], where minimum midshaft circumferences of the humerus and femur were used to predict mammalian mass (Table 1). The relationship between stylopodial circumference and body mass is thought to be highly conserved due to the fundamental weight-bearing role of these elements in quadrupedal terrestrial animals, regardless of posture [62–64]. However, such circumference-based methods may yield inflated mass estimates for taxa that have stylopodia of particularly irregular (non-circular) section profile (see *Megatherium* femora in Casinos [65]). Campione and Evans [63], in their work revisiting the Anderson et al.[16] method, concluded that their predictive equation based on combined humeral and femoral circumferences was inappropriate for highly fossorial species such as moles, due to their exceedingly apomorphic humeral morphology. We anticipate similar issues with the *Palorchestes azael* humerus, as its minimum circumference is distorted by distally-positioned pectoral and proximally-extensive supracondylar crests projecting from the diaphysis.

To assess this, we generated predictive equations based on humeral, femoral and combined circumferences so mass estimates could be compared. As per the Campione and Evans [63] method, we measured minimum humeral and femoral circumferences using thin measuring tapes. To their tetrapod dataset we added koala (*Phascolarctos cinereus*) and common wombat (*Vombatus ursinus*) data from Wroe et al. [27] to represent the closest living palorchestid relatives (total sample species n = 255, S2 Table). From this modified dataset we derived predictive equations using Model I (OLS) bivariate regressions using femoral, humeral and combined circumferences as the independent variables and body mass as the dependent variable (all log_10_ transformed). For each equation we report percent prediction error (PPE) and standard error of the estimate (SEE) as calculated through the MASSTIMATE package [66] in R [67]. Depending on availability of associated material, we then used one or more of these equations to estimate body mass for each palorchestid specimen. Additionally, we applied this method to revise previously-reported mass estimates for our extinct comparative vombatiform taxa, provided in S2 Table.

#### Systematic palaeontology

**Supercohort** MARSUPIALIA Illiger, 1811; *sensu* Cuvier, 1817

**Order** DIPROTODONTIA Owen, 1866

**Suborder** VOMBATIFORMES Woodburne, 1984

**Infraorder** VOMBATOMORPHIA Aplin & Archer, 1987

**Superfamily** DIPROTODONTOIDEA Archer & Bartholomai 1978

**Family** PALORCHESTIDAE Tate, 1948; *sensu* Archer & Bartholomai 1978

### Emended diagnosis

Members of Family Palorchestidae can be diagnosed based on craniodental features described by Woods [3] and emended by Trusler [36]. Additionally, they can be recognised from the following shared appendicular diagnostic features:

**Humerus.** Palorchestid humeri distinguished from diprotodontids by: Medial epicondyle strongly developed, causing substantial widening of distal humerus relative to length; Insertion facet for *mm*. *latissimus dorsi* & *teres major* ovoid in medial view, located at least halfway down diaphysis; Insertion facet for *mm*. *latissimus dorsi* & *teres major* has a posterolateral margin recurved as a ridge overhanging the posterior diaphysis.

**Unguals.** Palorchestid ungual phalanges distinguished from diprotodontids by: Ungual processes dorsoventrally deep and laterally compressed; Contour of ventral margin in lateral view angled and not a continuous arc; Extensor process dorsally deflected.

**Genus** *PALORCHESTES* Owen 1873

### Emended diagnosis

Members of the genus *Palorchestes* can be diagnosed based on craniodental features described by Woods [3] and emended by Trusler [36]. Additionally, they can be recognised from the following shared diagnostic appendicular features:

**Humerus.** *Palorchestes* humeri distinguished from diprotodontids by: Pectoral crest inferior margin falcate in medial and lateral views; Pectoral crest distal tip curls medially over bicipital sulcus. Differs from *Propalorchestes* in these humeral characters, as well as: Deltoid tuberosity well developed laterally; Capitulum and trochlea distal surfaces project equally distally in anterior view; Medial epicondyle more expansive relative to humeral length.

**Species** *Palorchestes azael* Owen, 1873

### Emended diagnosis

*Palorchestes azael* can be diagnosed based on craniodental features described originally by Woods [3] and emended in detail by Trusler [36]. Additionally, it may be distinguished from other *Palorchestes* species by the following diagnostic appendicular features:

**Humerus.** Differs from *P*. *parvus* in: Pectoral crest entirely separate from deltoid tuberosity on the lateral diaphysis; Olecranon fossa absent; Trochlear facet almost completely flat and inferiorly facing (articular surface not visible in anterior view); Trochlea deeper anteroposteriorly than capitulum in distal view; Rugose muscle scar for *m*. *epitrochleoanconeus* present on posterior surface of medial epicondyle.

**Ulna.** Differs from *P*. *parvus* in: Trochlear surface narrow, elongate and flattened.

**Species** *Palorchestes parvus* De Vis, 1895

### Emended diagnosis

*Palorchestes parvus* can be diagnosed based on craniodental features described by Woods [3] and supplemented by Trusler [36]. Additionally, they can be recognised from the following diagnostic appendicular features:

**Humerus.** Differs from *P*. *azael* in: Distal tip of pectoral crest and deltoid tuberosity connected by superolaterally-oriented oblique ridge as in extant wombats; Olecranon fossa present; Trochlear surface slightly convex and just visible in anterior view; Rugose muscle scar for *m*. *epitrochleoanconeus* absent.

**Ulna.** Differs from *P*. *azael* in: Trochlear surface wide and concave both proximodistally and mediolaterally.

**Genus** *PROPALORCHESTES* Murray 1986

**Species** *Propalorchestes novaculocephalus* Murray 1986

*Propalorchestes ponticulus* Murray 1990

### Emended diagnosis

Specimens belonging to the genus *Propalorchestes* can be diagnosed based on cranial features described in detail by Murray [68] and emended to include dental features in Murray [28]. Additionally, they can be recognised from the following diagnostic appendicular features:

**Humerus.** Differs from *Palorchestes* by: Pectoral crest thin and gracile medially; Pectoral crest terminates in distinct tubercle at distal end; Deltoid tuberosity on lateral diaphysis weak; Capitulum more distally extensive than trochlea in anterior view; Radial fossa present and distinct; Olecranon fossa present and deep.

## Comparative descriptions

### Palorchestes azael Owen 1873

#### Referred material

Measurements for all referred material below are provided in S1 Table.

**NMV P157144.** Associated partial skeleton including: mandible with diagnostic molars; left scapula fragment (glenoid cavity and base of spine with partial supraspinous/infraspinous/subscapular fossae only); right humerus fragment (distal two-thirds of shaft and epiphysis, missing capitulum and lateral epicondyle); left ulna fragment (proximal two-fifths, olecranon and anconeal surface damaged); left and right os coxae fragments (acetabular regions and partial ischia only); and femur fragment (diaphysis only). Unknown collector from unknown locality.

**Fig 1:**
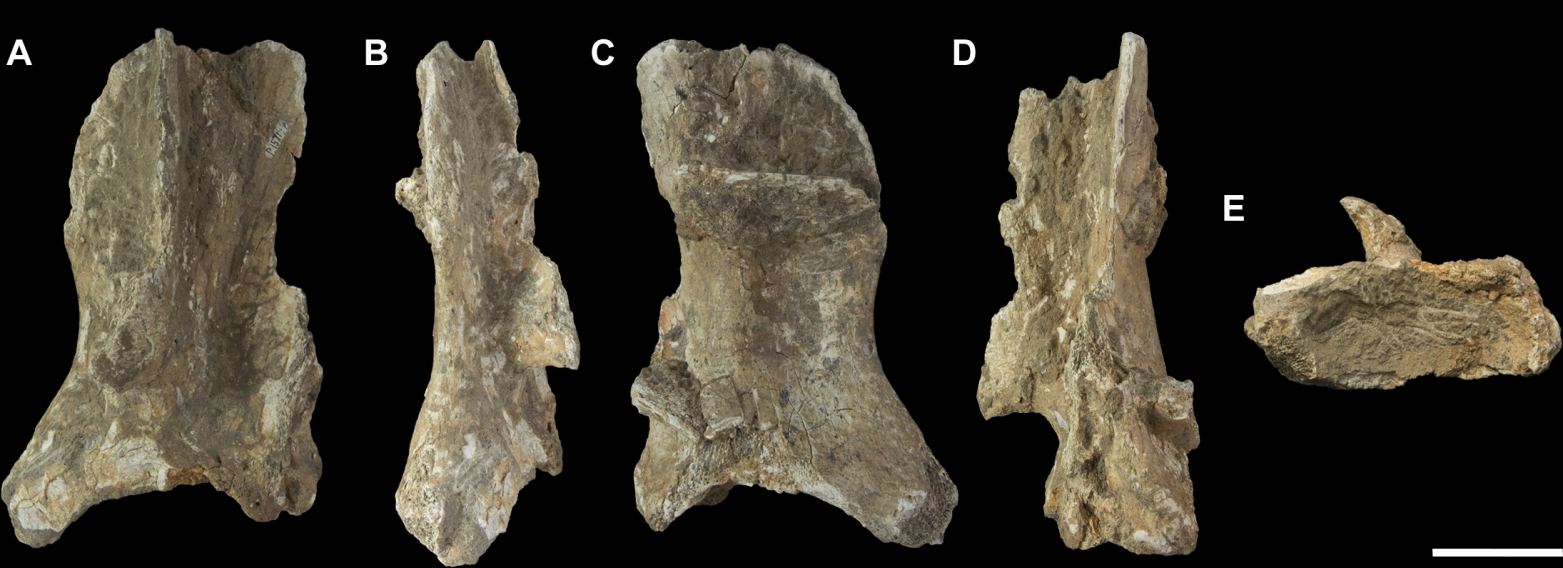
Left scapula fragment of *Palorchestes azael* NMV P157144. (A) lateral; (B) anterior; (C) medial; (D) posterior; (E) distal views. Scale bar 50 mm.

**Fig 2:**
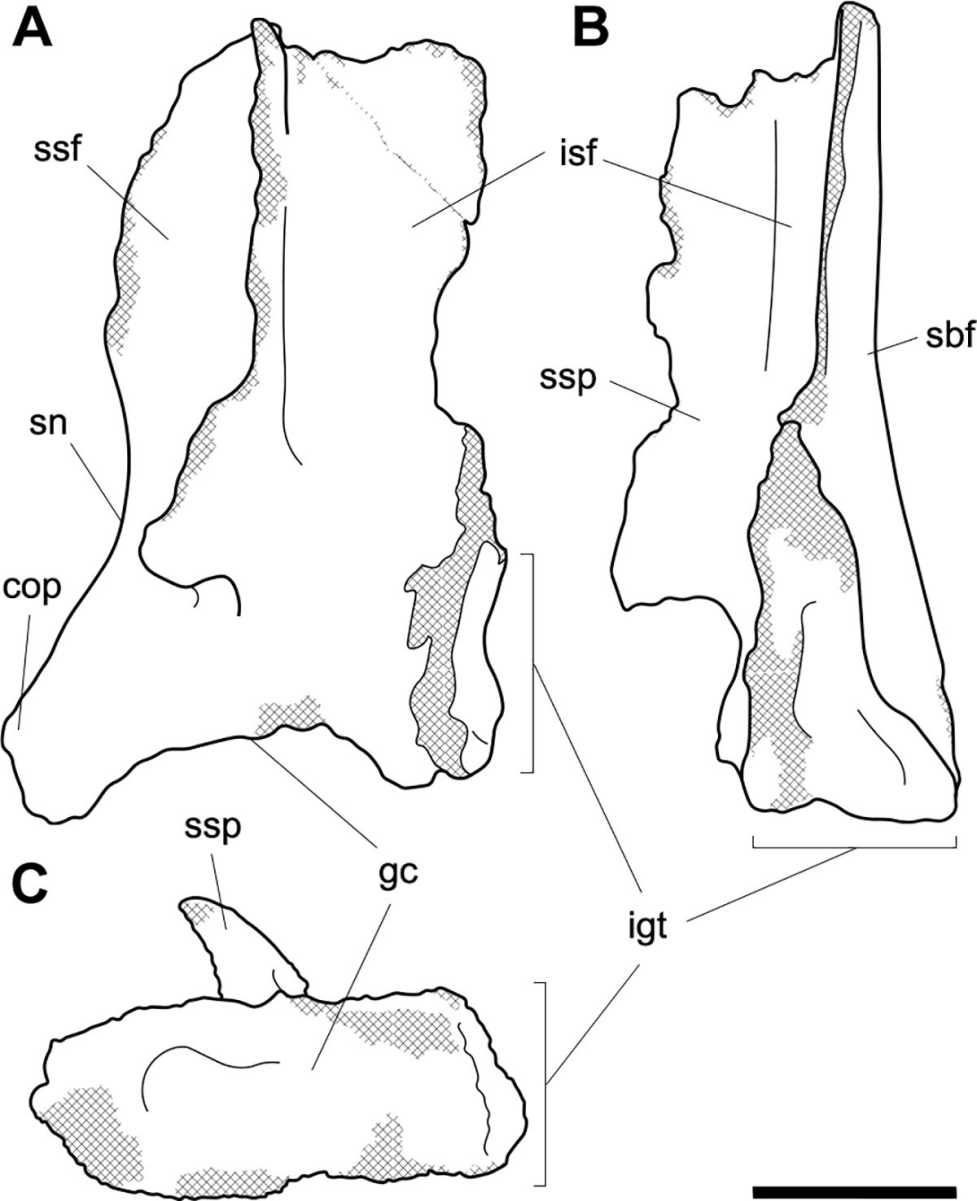
Labelled illustrations of *Palorchestes azael* left scapula NMV P157144. (A) lateral; (B) posterior; (C) distal views. Hatching indicates surface damage to cortical bone, dashed lines indicate inferred bone contours. Abbreviations: **cop**, coracoid process; **gc**, glenoid cavity; **igt**, infraglenoid tubercle; **isf**, infraspinous fossa; **sbf**, subscapular fossa; **sn**, scapular notch; **ssf**, supraspinous fossa; **ssp**, scapular spine. Scale bar 50 mm.

**Fig 3:**
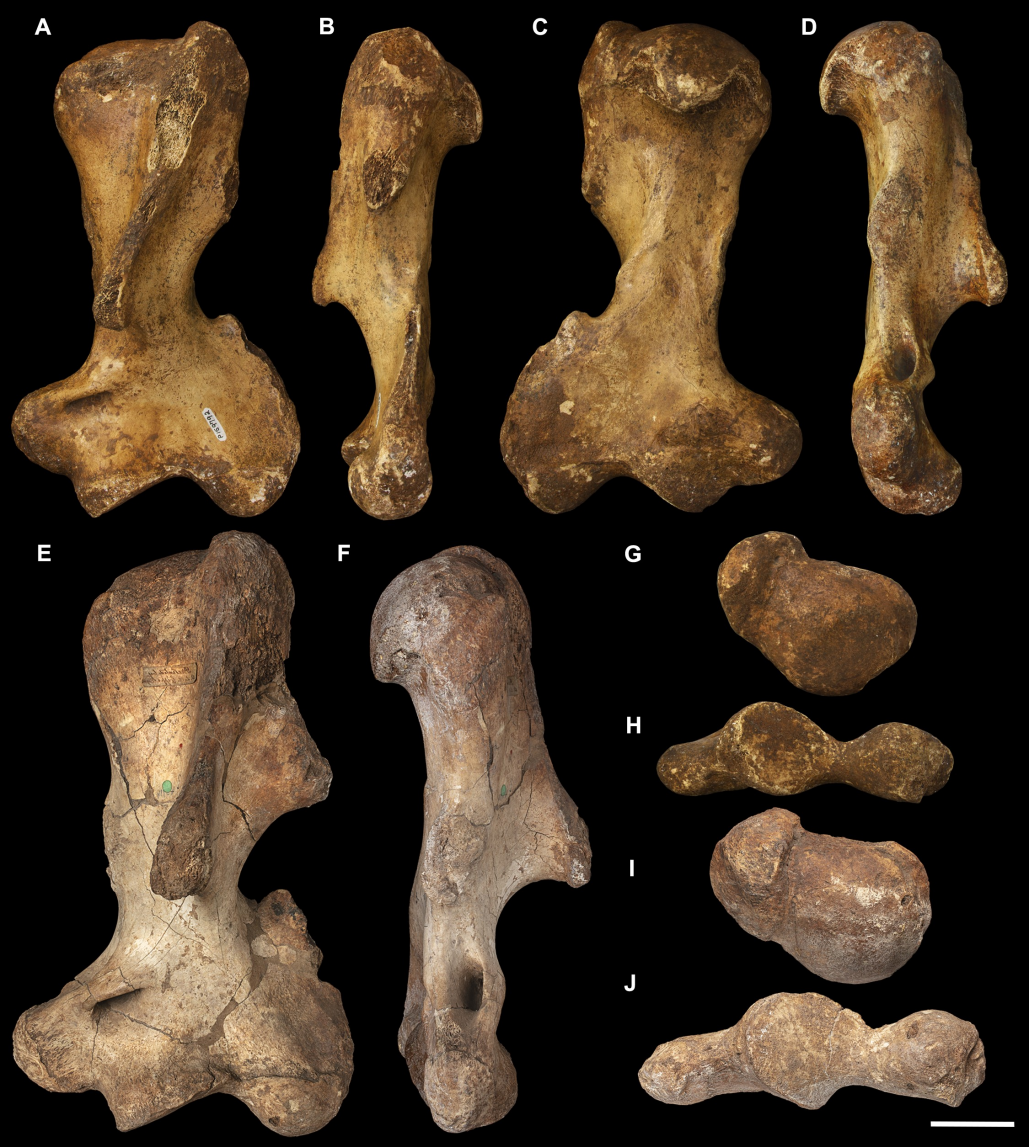
Humeri of *Palorchestes azael*. Left side humerus NMV P159792 (A-D, G-H) and right side humerus NHMUK PV OR 46914 (E-F, I-J, mirrored for comparison). (A) anterior; (B) lateral; (C) posterior; (D) medial; (G) proximal and (H) distal views. Right humerus NHMUK PV OR 46914 in (E) anterior; (F) medial; (I) proximal and (J) distal views. Scale bar 50 mm.

**Fig 4:**
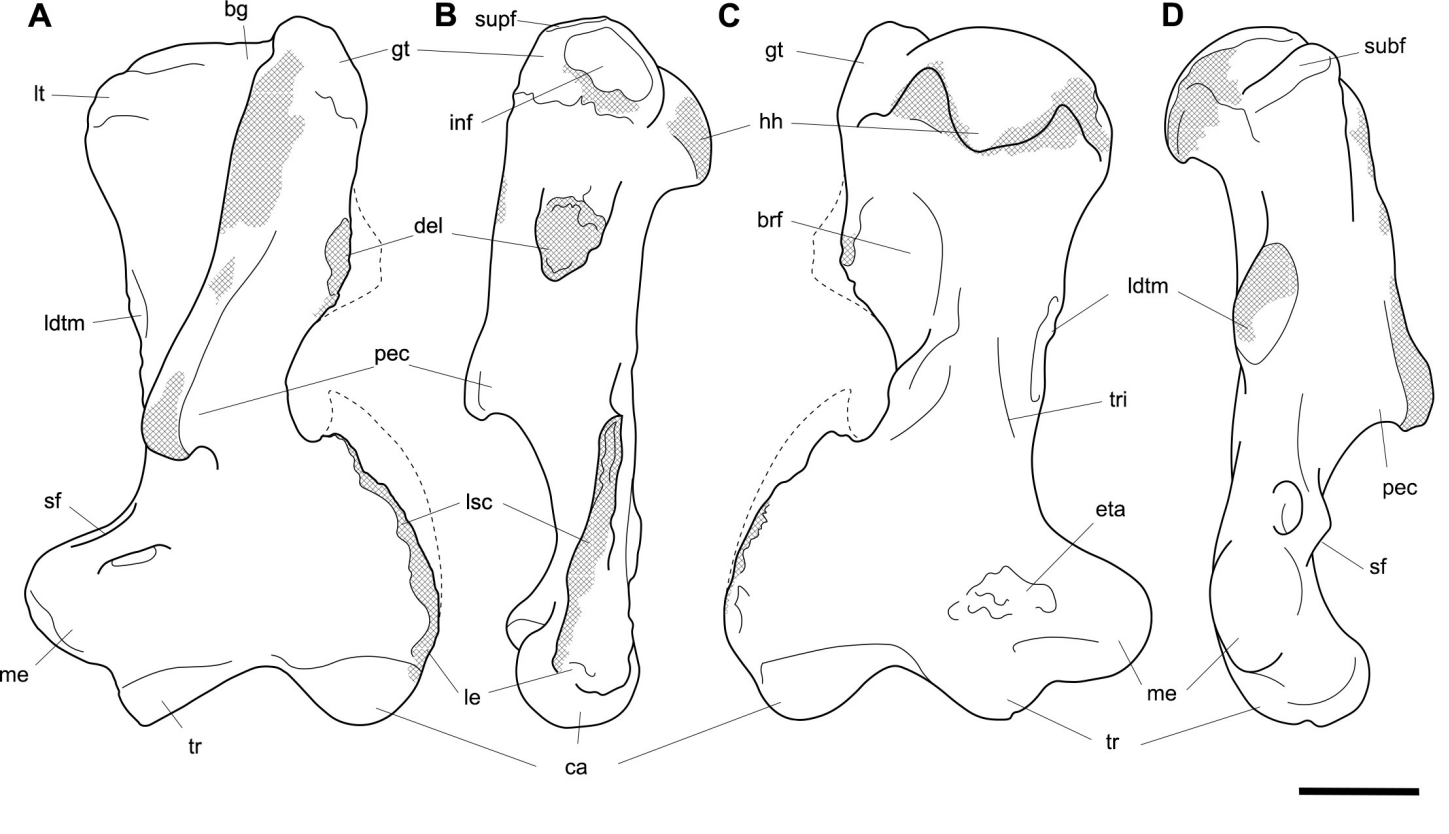
Labelled illustrations of the *Palorchestes azael* left humerus NMV P159792. (A) anterior; (B) lateral; (C) posterior; (D) medial views. Hatching indicates surface damage to cortical bone, dashed lines indicate inferred bone contours. Abbreviations: **bg**, bicipital groove; **brf**, fossa for *m*. *brachialis* origin; **ca**, capitulum; **del**, deltoid insertion; **eta**, origin for *m*. *epitrochleoanconeus*; **gt**, greater tubercle; **hh**, humeral head; **inf**, fossa for insertion of *m*. *infraspinatus;* **ldtm**, insertion for *mm*. *latissimus dorsi* and *teres major;* **le**, lateral epicondyle; **lsc**, lateral supracondylar crest; **lt**, lesser tubercle; **me**, medial epicondyle; **pec**, pectoral crest; **subf**, fossa for insertion of *m*. *subscapularis;* **supf**, fossa for insertion of *m*. *supraspinatus;* **sf**, supracondylar foramen; **tr**, trochlea; **tri**, origin for humeral heads of *m*. *triceps brachii*. Scale bar 50 mm.

**Fig 5:**
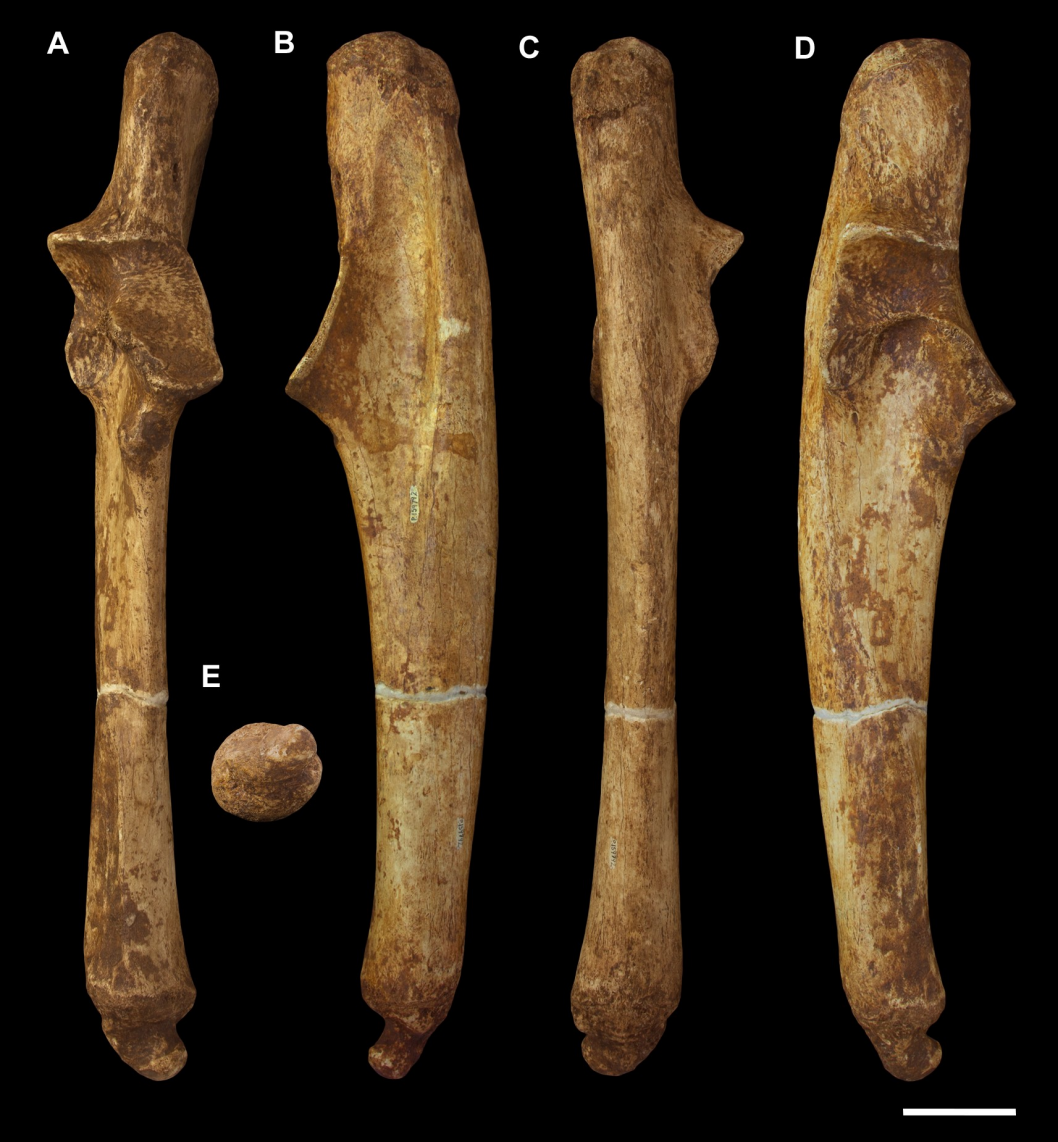
Right ulna of *Palorchestes azael* NMV P159792. (A) anterior; (B) medial; (C) posterior; (D) lateral; (E) distal views. Scale bar 50 mm.

**Fig 6:**
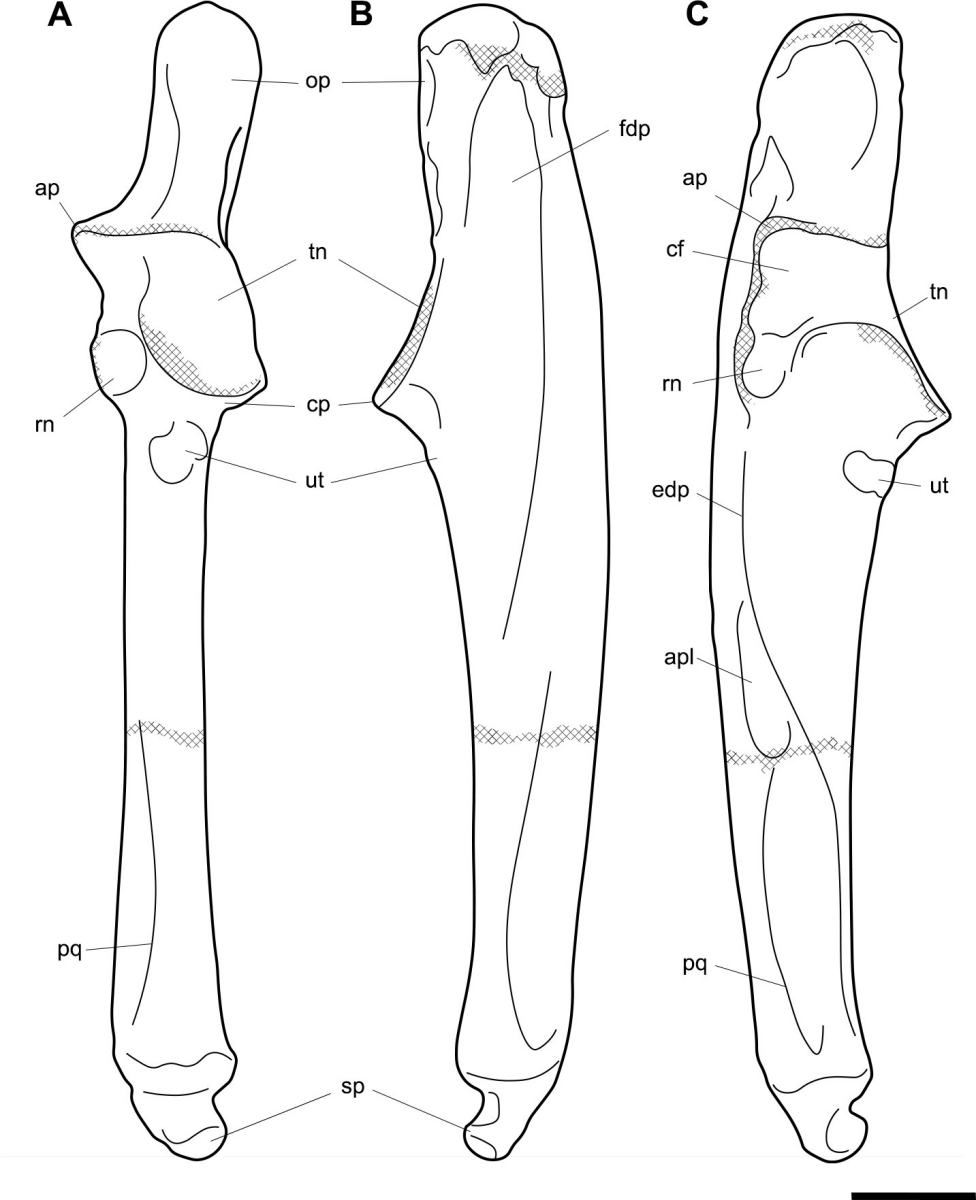
Labelled illustrations of the *Palorchestes azael* right ulna NMV P159792. (A) anterior; (B) medial; (C) lateral views. Hatching indicates surface damage to cortical bone. Abbreviations: **ap**, anconeal process; **apl**, origin for *m*. *abductor pollicis longus*; **cf**, capitular facet; **cp**, coronoid process; **edp**, origin for *m*. *extensor digitorum profundus;* **fdp**, fossa for origin of *m*. *flexor digitorum profundus;* **op**, olecranon process; **pq**, origin for *m*. *pronator quadratus;* **rn**, radial notch; **sp**, styloid process; **tn**, trochlear notch; **ut**, ulnar tuberosity. Scale bar 50 mm.

**NHMUK PV OR 46914.** Right humerus (diaphyseal cortex fractured and repaired). This element was figured and attributed to *Nototherium mitchelli* by Owen ([69], plate CXXVII Figs 1–6). Collected by W. L. R. Gipps from Castlereagh River, Mendooran, NSW, in 1875.

**NMV P159792.** Associated partial skeleton including: left humerus (superficial damage to upper third of pectoral crest, lateral supracondylar ridge); right humerus (middle third of shaft with partial pectoral crest, both epiphyses missing); left ulna (proximal two-thirds only); right ulna; right radius (open distal metaphysis with missing distal epiphysis); right os coxa; left os coxa (fragment with acetabulum, superior iliopubic ramus and ischial tuberosity only) with detached symphyseal epiphysis; left femur (shaft fragment only); right tibia (lateral proximal epiphysis damaged, hole in cortex of superior posterior diaphysis); ungual phalanx (dorsal process missing). Unfused metaphyses indicate bones were not yet fully mature. Humeral and ulnar morphology consistent with NMV P157144 but is smaller (S1 Table), other elements are here referred to the species by association with this humerus and ulna. Collected by F. Spry from Buchan Caves (probably Foul Air Cave), VIC, in 1907. Identified by Flannery and Archer in 1980, articulated humerus and ulna illustrated in Flannery and Archer [10].

**NMV P26534.** Right femur (superficial damage to rim of head, lateral margin of lateral condyle). Associated with NMV P159792 but given own registration when figured by Scott ([23], Plate 21) as a ‘phascolomyform’ femur. Collected by F. Spry from Buchan Caves (probably Foul Air Cave), VIC, in 1907.

**AM F5458.** Right ulna fragment (proximal third, olecranon damaged). Unknown collector from Wellington Caves, NSW, pre-1898.

**NHMUK PV OR 47828.** Left ulna fragment (proximal fourth, olecranon damaged). Collected by G. Bennett from unknown locality, circa 1877.

**AM F58934.** Right humerus fragment (middle third, both epiphyses missing). Collected by R. Wright from Ginnagulla, NSW, in 1977.

**NMV P252196.** Ungual phalanx (some erosion to edges of ungual crests). This element was tentatively attributed to *Thylacoleo* and photographed by Wakefield [70], pg. 79. Collected by N. A. Wakefield from Mount Hamilton Caves, VIC, in 1963.

**SAMA P28945, P28946, P28947.** Associated podial elements including: Two diagnostic ungual phalanges; associated intermediate phalanx. Collected by R. T. Wells from Victoria Fossil Cave, South Australia, 1970s.

**SAMA P55199.** Left humerus (distal half of immature bone with open articular metaphyses, and distal articular and medial traction epiphyses missing). Collected by J. S. Lockie from Puralka, VIC.

All following specimens were collected as part of the 1970s Gallus excavations at Keilor, VIC, found associated in the same ‘D Clay’ layer, and assigned to *Zygomaturus trilobus* by Marshall [50]. While no palorchestid material is yet reported from Keilor, these elements strongly differ from the morphology seen in *Zygomaturus*, and some were identified as palorchestid by Szalay [71], so we refer them to *Palorchestes azael* on the basis of their very large size and morphological similarity to elements of AM F58870.

**NMV P29619.** Metatarsal 5 (proximolateral tuberosity eroded).

**NMV P29620.** Metatarsal 4.

**NMV P29621.** Right cuboid.

**NMV P29622.** Right ectocuneiform.

**NMV P29623.** Right navicular.

**NMV P254089.** Left astragalus (posterolateral part missing). Figured in Szalay ([71], Fig 8.46 E-F).

**NMV P30723.** Right calcaneus (tip of calcaneal tuber missing). Figured in Szalay ([71], Fig 7.83 A-C).

#### Scapula (Figs 1 and 2)

The only known scapula for *P*. *azael* is part of the associated skeleton NMV P157144 (Figs 1 and 2). It is very eroded and encrusted with matrix which could not be safely removed. Preserving only the glenoid and distal portions, its proximodistal length, anteroposterior width and overall shape are not known. What little morphology is retained appears wombat-like, with the notable exception of the enlarged infraglenoid tubercle.

**Glenoid fossa.** The glenoid fossa is shallow, anteroposteriorly elongate and mediolaterally narrow as is typical of vombatiforms, although none of the outer circumference is preserved so the details of its shape are uncertain. The coracoid process is damaged and not clearly discernible.

**Supraspinous and infraspinous fossae.** Little of the supra- and infraspinous fossae are preserved, although the scapular notch is intact and appears more deeply curved than in vombatids and *Diprotodon* but less than in zygomaturines.

**Scapular spine.** The base of the distal scapular spine is preserved, showing it to curl anteriorly over the supraspinous fossa as in zygomaturines. The dorsal margin and acromion are missing.

**Infraglenoid tubercle.** The infraglenoid tubercle, though damaged, is very extensive mediolaterally and superoinferiorly, and deeply pitted. Roughly triangular in shape, it would have provided large origin for the long head of *m*. *triceps brachii*. It does not appear to have projected away from the glenoid to alter the posterior scapular border as it does in derived diprotodontids like *Diprotodon* and *Zygomaturus*, instead resembling the vombatid condition, though being thicker mediolaterally.

#### Humerus (Figs 3 and 4)

The humerus of *Palorchestes azael* is remarkable in its blade-like pectoral crest and its curiously flat trochlear surface (Figs 3 and 4). Of the two complete humeri figured, the larger (NHMUK PV OR 46914) shows marked increase in relative size and rugosity of muscle attachments, while the smaller humerus with its unfused metaphyses likely represents an osteologically-immature animal. Descriptions below are based on the former.

**Head.** The domed head extends its articular surface posteroinferiorly to provide increased posterior surface area, similar to that of other large-bodied diprotodontoids, rather than the superiorly-oriented head in vombatids. Viewed medially, this articular surface ‘beaks’ out from the humeral neck at its posteroinferior tip. The head is flanked anteriorly by greater and lesser tubercles separated by a broad and shallow bicipital sulcus.

**Greater tubercle.** The greater tubercle is rugose and projects above the humeral head, narrowing to a proximally rounded tip resembling that of *Lasiorhinus* in shape and proportion. Discrete proximal (for *m*. *supraspinatus*) and lateral (for *m*. *infraspinatus*) facets are present. The tubercle continues inferomedially to give rise to the pectoral crest. Viewed laterally, the greater tubercle is broad and occupies two thirds of the total anteroposterior depth of the proximal humerus.

**Lesser tubercle.** The lesser tubercle in *P*. *azael* is comparatively poorly developed among diprotodontoids. In medial view, it extends obliquely from its uppermost tip anteriorly to its distal edge posteriorly, terminating proximal to the tip of the beaked humeral head. A medially-oriented facet for insertion of *m*. *subscapularis* is, like in other palorchestids, narrow and steeply oblique, though less steep than in *Nimbadon*.

**Deltoid tuberosity.** Viewed anteriorly the deltoid tuberosity is extensive and approximately triangular in shape, projecting strongly from the lateral diaphysis and contour of the greater tubercle above. It tapers to a rugose, bulbous terminus at its apex which lies a third of the way down the humeral shaft. The posterolateral lip of this deltoid tuberosity curls posteriorly to form the lateral margin of the fossa for *m*. *brachialis*. The clear separation of this large tuberosity from the pectoral insertion crest, contrasting with morphology of other palorchestids and vombatids, suggests a strongly developed scapular deltoid in *P*. *azael* and a reduction in the clavicular deltoid, with no distinct attachment site visible for this muscle. This arrangement resembles that seen in *Diprotodon*, although in *P*. *azael* the deltoid tuberosity differs strongly from that taxon in its triangular form and far more proximal position relative to the pectoral crest.

**Pectoral crest.** The pectoral crest is a distinctive feature of the *P*. *azael* humerus, originating from the greater tubercle just lateral to the humeral midline. It follows an oblique inferomedial path along the anterior shaft, becoming a laminar crest with a rectangular sectional profile which increases in height and thickness as it curls around medially. At its distal tip it swells to a bulbous terminus in the largest specimens, which then re-joins the shaft via a recurved, sickle-shaped ‘pulley’, presumably for the passage of *m*. *biceps brachii* descending along its medial plane. At its zenith the anterior projection of this pectoral crest from the shaft is greater than in any other vombatiform studied, in both relative and absolute extent.

**Tuberosity for *mm*. *teres major* and *latissimus dorsi*.** An elongate, ovoid muscle scar lays halfway down the medial aspect of the humeral shaft for the insertion of *mm*. *teres major* and *latissimus dorsi*. Its posterior edge overhangs and sharply demarcates the posterior from the anterior shaft surface. This insertion is relatively larger, better developed and more distal than in any other vombatiform studied.

**Diaphysis.** The humeral diaphysis is highly irregular in section and in profile due to the many large muscle attachment crests along its relatively short length. It is stout and broad in anterior view with an almost ‘hourglass’ silhouette due to the flared proximal and distal ends and narrow constriction in line with the lateral supracondylar crest. In lateral view the posterior contour of the shaft is near-flat (as is typical for diprotodontoids), while the anterior contour is dominated by the hooked pectoral crest. The posterior diaphyseal surface features obvious scars for the origins of the humeral heads of *m*. *triceps brachii*. At the base of the humeral neck is a distinct fossa for the origin of *m*. *brachialis*, so expansive as to nearly reach the shaft midline as in *Phascolonus*. This fossa curves laterally beneath the deltoid tuberosity and anterior to the lateral supracondylar crest. There is no olecranon fossa on the posterior aspect of the distal humeral shaft, which is flat and smooth but for a marked rugose patch immediately proximal to the trochlea. This forms the origin for *m*. *epitrochleoanconeus*.

**Lateral epicondyle.** The lateral epicondyle projects only slightly beyond the lateral margin of the capitulum. In lateral view, the lateral epicondyle is thick inferiorly before curving proximally up and tapering into the lateral supracondylar crest, a sheet of bone that presumably terminated in a hooked process as in most other vombatiform marsupials. Almost the entire edge of this crest is damaged in all specimens observed. However, the concave, arced profile that remains at the top of the crest, curling superiorly from the lateral shaft, suggests such a hooked process was once present. This crest would have provided origin for *mm*. *brachioradialis* and *extensor carpi radialis*.

**Trochlea.** The trochlea is another notably unique feature of the *P*. *azael* humerus. Its articular area is completely flattened and would have allowed almost no parasagittal movement of the ulna. Rather than a pulley-shaped or domed articulation as in other vombatiforms, it is a simple, distolaterally-facing ovoid facet oriented ~115° to the neighbouring capitulum. In distal view the trochlea is offset anteriorly from the dorsal (coronal) plane of the humerus, projecting cranially much more than the smaller capitulum. In the larger *P*. *azael* specimens the posterior trochlear surface curves superiorly to provide slightly more anconeal articulation than in the smaller humeri.

**Capitulum.** In anterior view the capitulum is near hemi-spherical and lacks an obvious radial fossa proximally. In distal view it is ~20% smaller than the trochlea. Posteriorly, the joint surface for articulation with the anconeal process of the ulna extends slightly higher than on the anterior side of the capitulum and is slightly elevated from the posterior shaft surface.

**Supracondylar foramen.** The bridge defining the ceiling of the supracondylar foramen is broad proximally and tapers somewhat at its mediodistal end. This bridge lies almost horizontally along the corresponding contour of the medial epicondyle, giving the underlying foramen a vertical orientation unlike the more oblique foramina of other vombatiforms (where present). The foramen is approximately four times longer than its breadth.

**Medial epicondyle.** The medial epicondyle in anterior view is rounded and projects strongly medially, its axis perpendicular to the vertical axis of the humeral shaft in both dorsal and transverse planes. The medial epicondyle constitutes approximately 32% of the total distal humeral width, making it the largest, both relatively and absolutely, among the vombatiforms.

#### Ulna (Figs 5 and 6)

Generally, the ulnar shape and proportions of *P*. *azael* resembles that of extant wombats, *Ngapakaldia* and *Thylacoleo* much more than other large diprotodontoids, albeit with some significant alterations (see S2 Fig). Overall the ulna is straight when viewed anteriorly, while in lateral view it is slightly convex posteriorly, giving a bowed appearance (Figs 5 and 6).

**Olecranon process.** The olecranon is very enlarged and elongate, representing nearly a quarter of the total ulnar length, and in lateral view appears quadrangular with a slight bulbous projection posteriorly. Unlike in the diprotodontid ulna, the olecranon lies along the axis of the shaft with no posterior deflection, and medial deflection is only very slight–less than in extant wombat or *Ng*. *bonythoni* ulnae and much less than the medially-curled olecranon in *Phascolonus*. The olecranon is deep in the sagittal plane, slightly more so than at the midshaft of the bone, and does not taper proximally as extant wombats do. The transverse width at the dorsal edge of the olecranon is around half its dorso-ventral depth.

**Trochlear notch.** The trochlear notch and trochlear surface are flattened (in agreement with the corresponding articular surface of the humerus), with only the slightest concavity as the trochlear surface ramps distally to form the coronoid process. The orientation of the trochlear surface is slightly less steep than in vombatids, being approximately 45° to the axis of the shaft. This contrasts with the near-horizontal equivalent in diprotodontids and is not nearly as concave as the latter. In *P*. *azael* this flat trochlear surface lies on a medially-projecting platform, creating a deep elongate fossa beneath it on the medial shaft surface as in vombatids and *Ng*. *bonythoni*. The floor of this fossa represents the narrowest lateral dimension of the ulna overall and provided origin for *m*. *flexor digitorum profundus*. The lateral semilunar facet for the humeral capitulum is concave and less than half the proximodistal length of the trochlear surface.

**Coronoid process.** In lateral view the coronoid process is robust with a triangular profile, owing to the flattened trochlear surface on the proximal side and the gradual slope of diaphyseal bone on the distal side. It is less dorsoventrally extensive relative to the diaphyseal thickness than in all other comparative taxa observed.

**Anconeal process.** The anconeal process is strongly laterally deflected, its axis in the transverse plane offset by ~90° to that of the shaft so that in medial view it is not visible.

**Radial notch.** The radial notch projects laterally and is similar to the anconeal process in its extent. The radial notch is more posteriorly offset than any other taxon studied, positioned very near the dorsal border of the shaft. A deep, inset ligamentous pit lies directly mediad to it for attachment of the annular ligament. Like the notch, this pit is more dorsally positioned than in other vombatomorphs.

**Ulnar tuberosity.** The ulnar tuberosity for insertion of *mm*. *brachialis* and *biceps brachii* is approximately 15 mm in diameter. It is raised, bulging and is situated well distal to the coronoid process and radial notch on the lateral aspect of the ventral shaft margin. In relative displacement of the tuberosity from the coronoid, the *P*. *azael* ulna is surpassed only by that of *Phascolonus*.

**Diaphysis.** The diaphysis varies in section profile along its length from ovo-quadrangular proximally, lachrymiform at midshaft due to the thickened posterior border, to subcircular at the distal epiphysis. This circular distal section contrasts with the mediolaterally-flattened epiphyses of vombatid and *Ng*. *tedfordi* ulnae, being more similar to those of *Diprotodon* and *Zygomaturus*, likely corresponding to changes in directional stresses associated with increased body mass. The *P*. *azael* ulnar shaft also varies in anteroposterior depth along its length, being deepest at the ulnar tuberosity and gently tapering distally. The lateral diaphyseal surface is largely smooth and lacks the marked longitudinal scarring seen in diprotodontid ulnae. The most obvious muscle attachments are two large ovoid scars–one arising laterally at the approximate midshaft (*mm*. *abductor pollicis longus* and *extensor digitorum profundus*), the other occupying the distal third of the lateral diaphysis (*m*. *pronator quadratus*). The latter scar strongly resembles the condition in *Ngapakaldia*.

**Styloid process.** The styloid process is thick and distinctly anteromedially deflected like in vombatids, tapering from a wide, dorsolaterally-elongate base to a small bulb featuring a distally-facing convex articular facet for the triquetrum. The styloid is ovoid in distal view and much smaller than the surrounding epiphysis, similar to that of *Ng*. *tedfordi* in proportions and shape and unlike the inflated, bulbous styloid in *Ng*. *bonythoni*, *Neohelos*, *Diprotodon* and *Zygomaturus*.

#### Radius (Figs 7 and 8)

**Fig 7:**
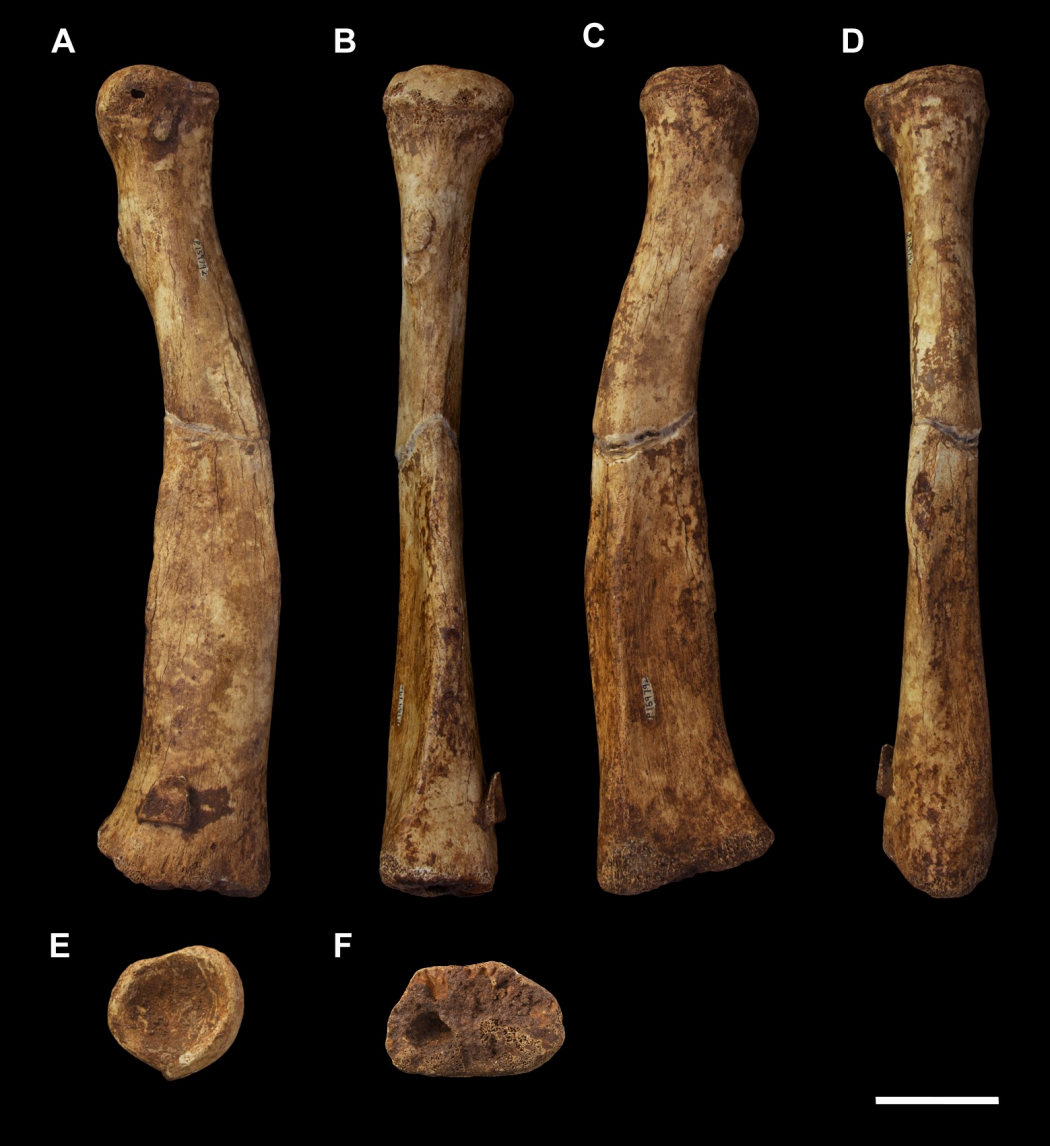
Right radius of *Palorchestes azael* NMV P159792. (A) dorsal; (B) medial; (C) ventral; (D) lateral; (E) proximal; (F) distal views. Scale bar 50 mm.

**Fig 8:**
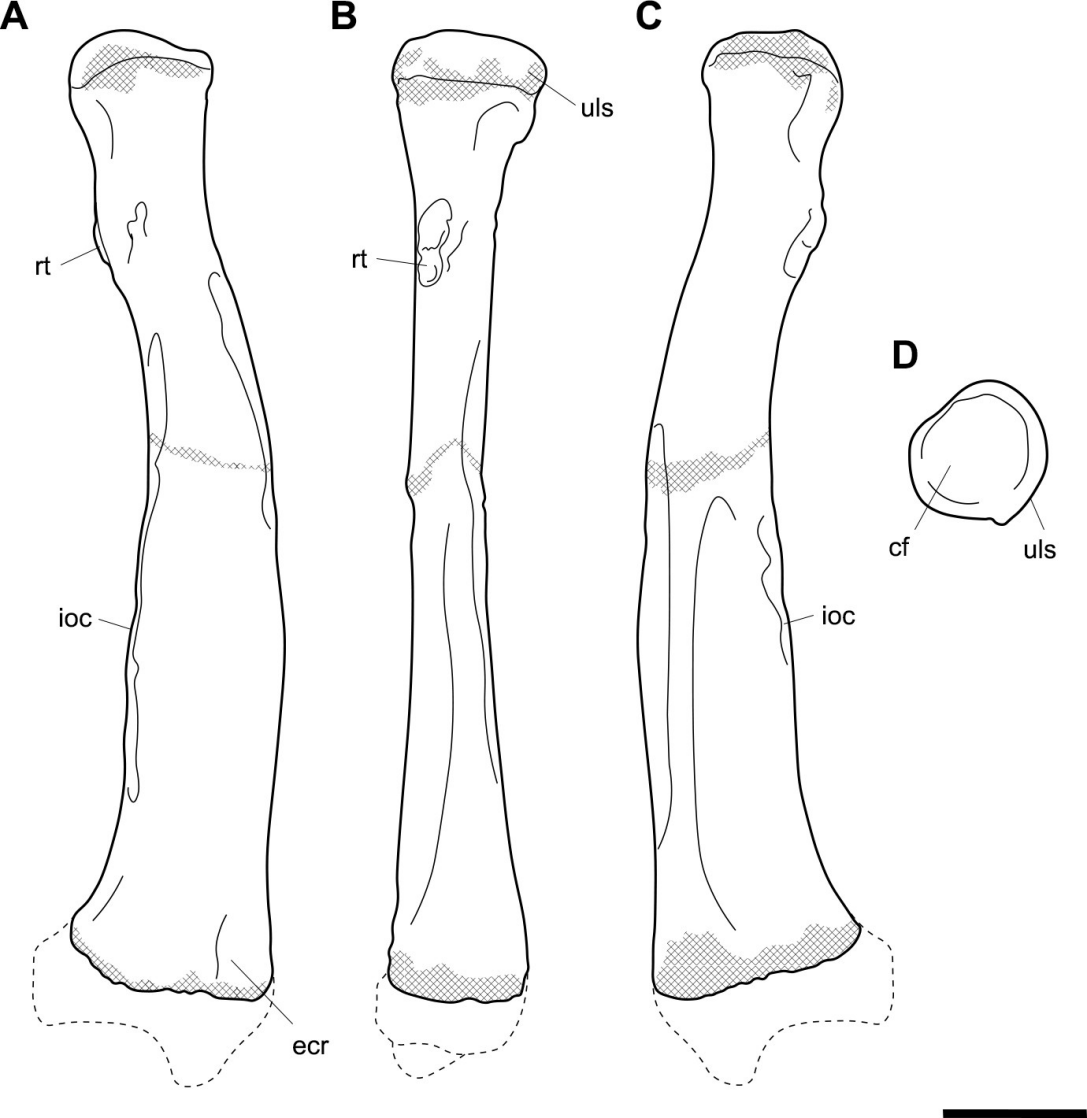
Labelled illustrations of the *Palorchestes azael* right radius NMV P159792. (A) dorsal; (B) medial; (C) ventral views. Hatching indicates surface damage to cortical bone, dashed lines indicate inferred bone contours. Abbreviations: **cf**, capitular fossa; **ecr**, notch for tendon of *m*. *extensor carpi radialis;* **ioc**, interosseous crest; **rt**, radial tuberosity; **uls**, articular surface for ulna. Scale bar 50 mm.

The palorchestid radius is most like that of *Vombatus* in overall proportions (Figs 7 and 8). The angle of the head and neck, and the degree to which the diaphysis curves medially down toward the styloid process is very vombatid. Like wombats, the radial midshaft is triangular in section. It becomes progressively more mediolaterally expanded to appear flatter and bulkier in distal section than the wombat though still distinctly trapezoidal, unlike the triangular distal profile of *Diprotodon* and *Phascolonus* radii.

**Head.** In proximal view, the perimeter of the capitular fossa is suboval, with the slight transverse elongation seen in wombats and *Ng*. *tedfordi*, rather than the more truly circular fossae of *Diprotodon* and *Phascolonus* radial heads. The hemispherical fossa is deeply cupped, closest in relative depth to that of *Ng*. *tedfordi*, though lacking the distinct craniolateral tilt and sharp rim edge of the latter. The articular surface for the ulna occupies two-fifths of the capitular circumference, roughly equivalent to the vombatid condition and less than in *Ng*. *tedfordi*.

**Radial tuberosity.** The radial tuberosity for insertion of *m*. *biceps brachii* is more distally situated on the shaft than in *Vombatus*, closely resembling *Lasiorhinus* and *Ngapakaldia* spp. in relative positioning. However, the radial tuberosity in *P*. *azael* is not prominent and does not project from the shaft anything like the marked tuberosities seen in the radii of *Phascolonus*, extant wombats or *Ngapakaldia* spp., suggesting reduced size or contractile force in *m*. *biceps brachii*.

**Diaphysis.** The interosseous crest begins immediately distal to the radial tuberosity and is less sharply defined than in extant wombats, and dramatically less so than the broad flange present in *Phascolonus*. The crest continues along the lateral border of the diaphysis, forming the ‘apex’ of the triangular midshaft section profile and terminating in a slightly rugose area ¾ of the way down the shaft. This rugosity differs strongly from the *Vombatus* and *Phascolonus* forms in which it is deeply pitted. The shaft becomes extremely thick and robust distally, more so than any other taxon studied. The medial diaphyseal border is dominated by the insertion scar for *m*. *pronator quadratus*, which is very similar in positioning and extent to that seen in extant wombats.

**Distal end.** The distal section profile of the *P*. *azael* radius is trapezoidal, with a flattened dorsal surface parallel to the slightly concave ventral surface. This contrasts with the dorsoventrally broad, triangular distal profile of diprotodontid and *Vombatus* radii, the flattened palorchestid state being most similar to that of *Lasiorhinus* and *Ngapakaldia*.

#### Ungual (Figs 9 and 10)

**Fig 9:**
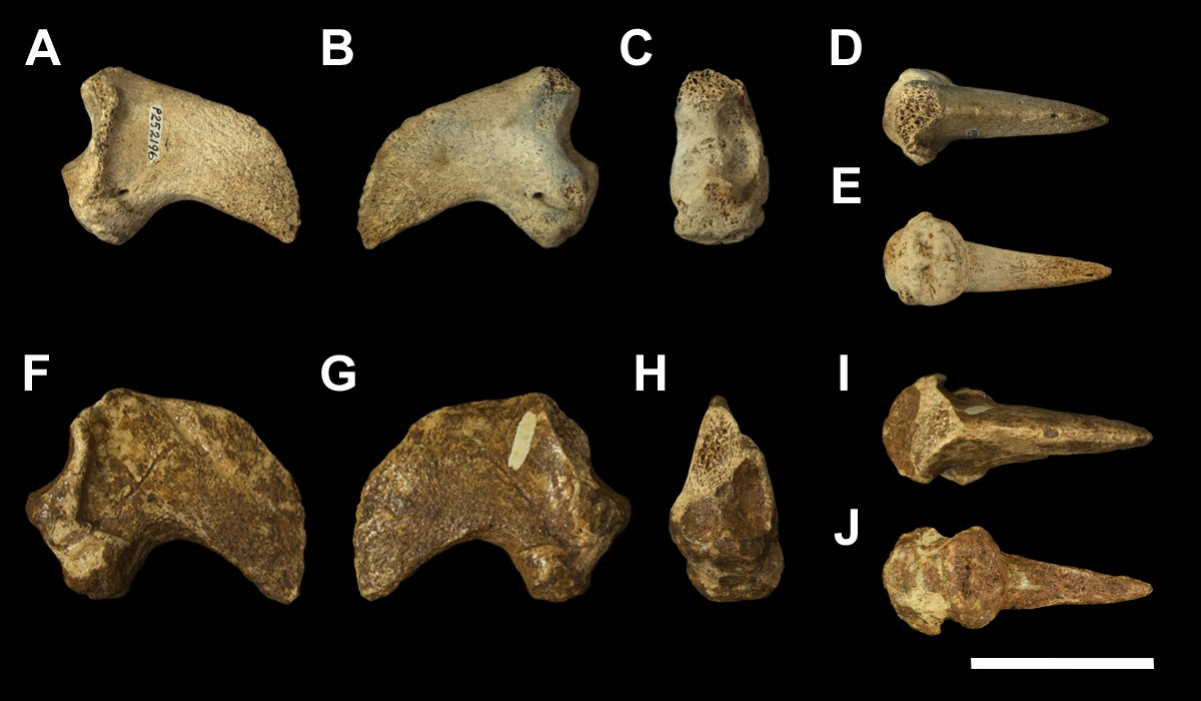
Unassociated ungual phalanges of *Palorchestes azael*. NMV P252196 (A-E) and NMV P159792 (F-J) in (A,F) lateral; (B, G) medial; (C, H) proximal; (D, I) dorsal; (E, J) volar views. Scale bar 50 mm.

**Fig 10:**
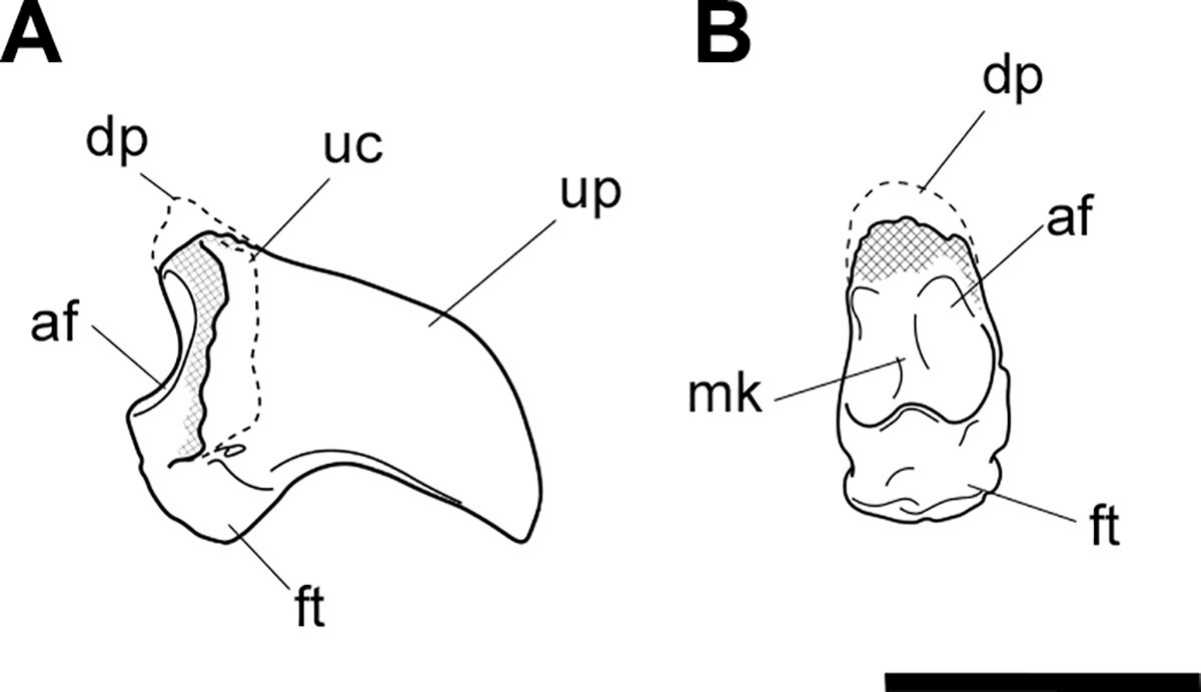
Labelled illustration of ungual phalanx of *Palorchestes azael* NMV P252196. (A) Lateral; (B) proximal views. Hatching indicates surface damage to cortical bone, dashed lines indicate inferred bone contours. Abbreviations: **af**, articular facet; **dp**, dorsal process; **ft**, flexor tubercle; **mk**, median keel; **uc**, ungual crest (estimated); **up**, ungual process. Scale bar 50 mm.

Palorchestid unguals are morphologically distinct from all other vombatiforms in their deep, laterally-compressed, lunate shape (Figs 9 and 10). Overall, they most closely resemble those of *Phascolarctos* or *Nimbadon*, but in palorchestids they are dorsoventrally deeper and larger relative to the proximal and intermediate phalanges.

**Ungual crests.** When preserved, these crests are very well-developed plates of bone, projecting distally to an even greater extent than in *Nimbadon* or extant *Phascolarctos*. The resulting sulcus would have provided a deep and secure attachment for the proximal rim of the keratinous claw sheath.

**Proximal end.** The articular facets for the intermediate phalangeal condyles are transversely narrow, deep semilunate notches with a low median crest. In proximal view the facets are slightly tapered dorsally and broader ventrally, giving an elongate trapezoidal shape overall in this aspect. The dorsal extensor process is expanded and is more dorsally deflected than in any other vombatiform, overhanging the joint while allowing a degree of hyperextension. Dilated and robust flexor tubercles on the plantar surface are larger relative to the rest of the ungual than other large diprotodontoids. These tubercles are as mediolaterally broad as the articular facets, giving a more quadrate shape to this region of the ungual in proximal view unlike the ventrally tapered appearance in *Ngapakaldia* and *Neohelos*.

Whether the assigned *P*. *azael* unguals are manual or pedal is not known.

#### Os coxa (Figs 11 and 12)

**Fig 11:**
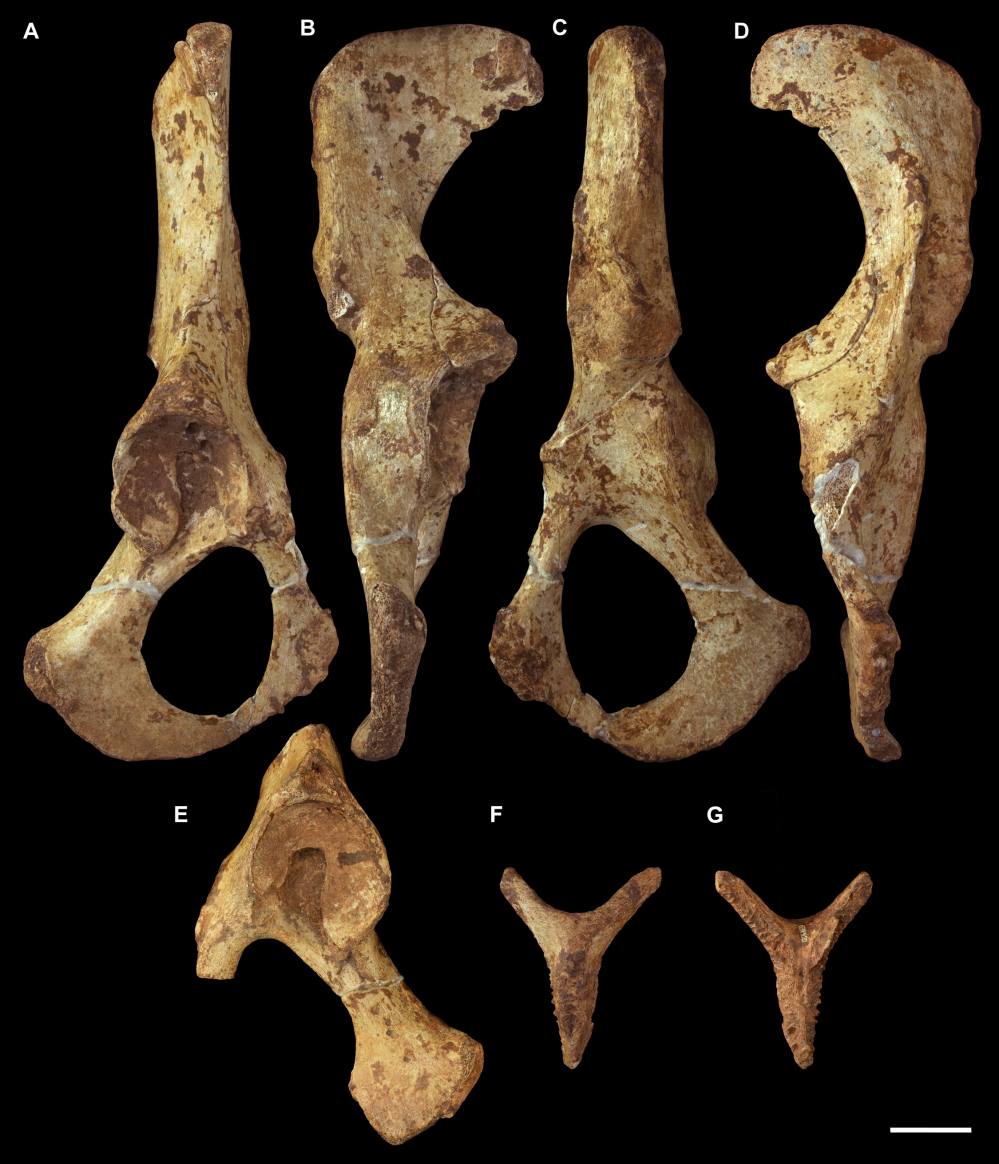
Associated pelvic elements of *Palorchestes azael* NMV P159792. Right side os coxa in (A) lateral view; (B) dorsal; (C) medial; (D) ventral views. Left side os coxa fragment in (E) lateral view. Pubic symphyseal epiphysis in (F) anterior; (G) posterior view. Scale bar 50 mm.

**Fig 12:**
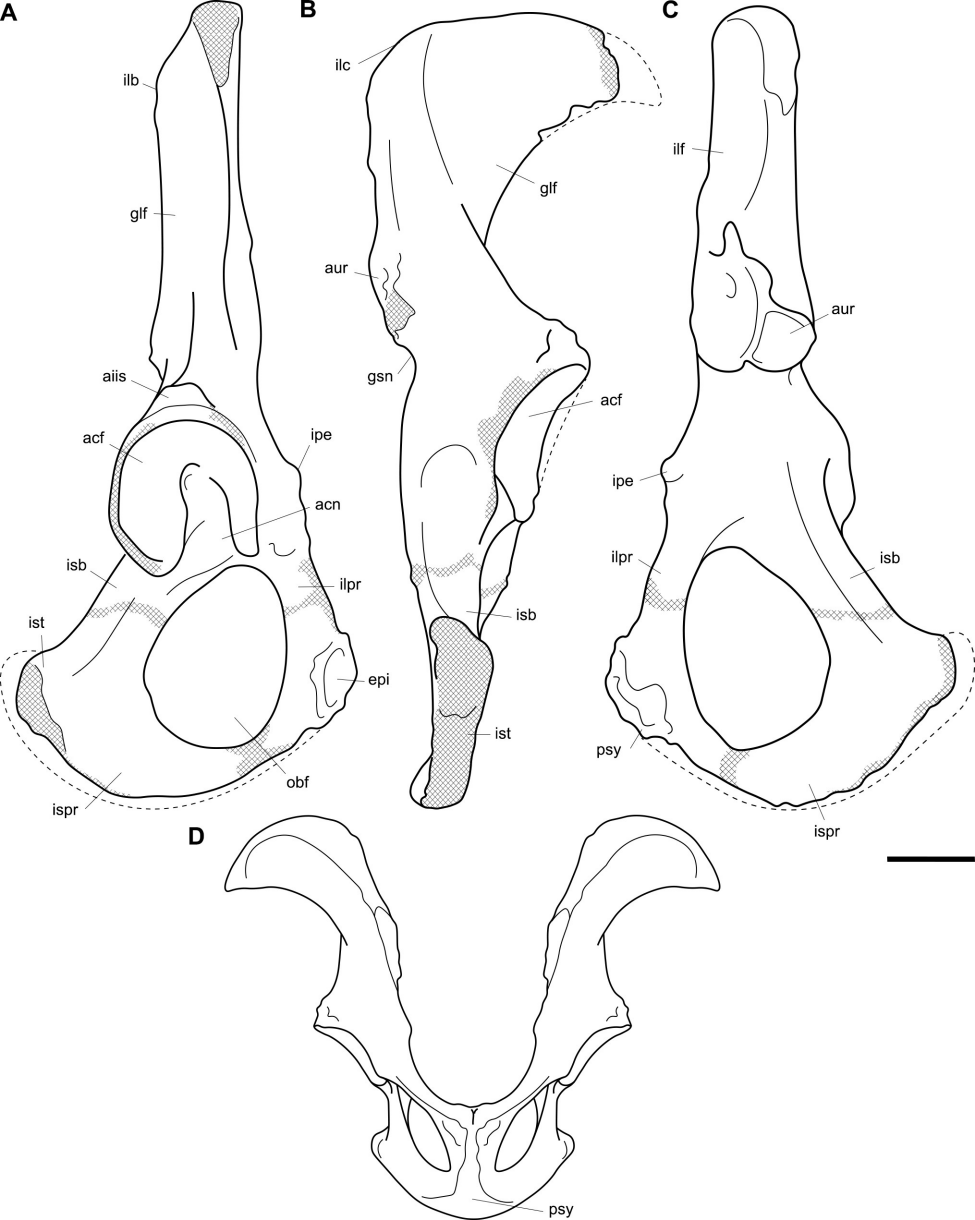
Labelled illustrations of the *Palorchestes azael* os coxae. (A) right side os coxa, lateral view; (B) right side os coxa, dorsal view; (C) right side os coxa, medial view; (D) reconstructed pelvic girdle, superoventral view (this view not to scale). Hatching indicates surface damage to cortical bone, dashed lines indicate inferred bone contours. Abbreviations: **acf**, acetabular fossa; **acn**, acetabular notch; **aiis**, anterior inferior iliac spine; **aur**, auricular surface; **epi**, facet for articulation with epipubic bone; **glf**, gluteal fossa; **gsn**, greater sciatic notch; **ilb**, iliac blade; **ilc**, iliac crest; **ilf**, iliacus fossa; **ilpr**, iliopubic ramus; **ipe**, iliopectineal eminence; **isb**, ischial body; **ispr**, ischiopubic ramus; **ist**, ischial tuberosity; **obf**, obturator foramen; **psy**, pubic symphysis. Scale bar 50 mm.

The os coxa overall is gracile and slender, lacking broad muscle attachment surfaces or pronounced tuberosities (Figs 11 and 12).

**Iliac blades.** In *P*. *azael* the iliac blades are sickle-shaped, with mediolaterally narrow dimensions, quite unlike the laterally flared ilia of *Zygomaturus* and *Diprotodon*. In relative length the ilia are intermediate between those taxa and the elongate ilia of extant wombats. They resemble *Neohelos* ilia but have a more deeply concave lateral margin and are slightly broader. The best-preserved specimen is missing the lateral tip of the blade and the epiphyseal rim along its proximal margin–their contours are estimated in Fig 12. In lateral view the ilium is oriented to create a 140° angle with the body of the ischium, a much more dorsally-rotated position than in *Zygomaturus* or the almost aligned *Diprotodon*. The ilium is slightly twisted anteriorly causing the posterior (gluteal) blade surface to be visible in lateral aspect.

**Ischial body.** The body of the ischium in *P*. *azael* is long, with a flattened ovoid profile. It is relatively longer than in *Diprotodon* or *Zygomaturus*, with a similarly upturned distal end for the ischial tuberosity. Unlike extant wombat ischia, there is no ischial spine present.

**Ischial tuberosity.** The ischial tuberosity is a gracile, elongate, weakly rugose structure in posterior view and lacking the distinct triangular shape with posterolateral projections seen in wombats. It appears narrower and shorter than in all other diprotodontoids studied.

**Acetabulum.** The shape of the acetabular margin in lateral view is arched, with a slightly tapered point at its superior margin greatly overhanging the acetabular fossa. Inside the acetabulum the notch is narrow, leaving a large articular surface area within the socket resembling the condition in *Zygomaturus* in extent and shape, though less buttressed around the perimeter than the latter. There is a deeply excavated fossa for the ligamentum teres. The acetabulum overall is shallower and less circular than in diprotodontids and vombatids, and more posterolaterally oriented than in the latter.

**Anterior inferior iliac spine.** The anterior inferior iliac spine for the origin of *m*. *rectus femoris* is located inferiorly and close to the acetabular margin as in *Zygomaturus*, though it is a narrower muscle scar in *P*. *azael*.

**Iliopectineal eminence.** The iliopectineal eminence is smaller, sharper and more distinct in *P*. *azael* than any other taxon studied.

**Iliopubic ramus.** The iliopubic ramus in *P*. *azael* is slender overall, with a large ovoid fossa for articulation with the epipubic bone. This ramus is relatively longer and more slender than in *Ngapakaldia*, *Phascolonus* or *Diprotodon* and more inferiorly directed than in *Neohelos*, with more circular section profile.

**Obturator foramen.** The obturator foramen is ovotriangular and slightly elongate, with the apex of its shape directly under the pectineal origin, similar to that seen in *Ngapakaldia* and *Neohelos*, though relatively broader than both.

**Inferior/ischiopubic ramus.** The ischiopubic ramus is long relative to the ischial body, indicating a deep pelvic cavity as in diprotodontids. It is dorsoventrally shallow at its pubic end. It deepens dorsoventrally as it extends posteriorly, though not as much as in *Phascolonus*, upturning but not increasing in transverse thickness as it gives rise to the ischial tuberosity.

**Auricular surface.** The auricular surface is rugose, with two distinct articular facets; a small one facing medially and a longer, deeper one facing more anteriorly. The sacral surface is craniocaudally short compared to that of *Zygomaturus* and much shorter than in *Phascolonus*, being more similar to the *Ngapakaldia* form. Inferior to the auricular surface, a small greater sciatic notch is visible in posterior view.

**Detached symphysial epiphysis.** Associated with the NMV P159792 os coxae of *P*. *azael* is the detached symphyseal epiphysis from the ventral pubis. This provides the subpubic angle and angle of the anterior pelvic brim, indicating *P*. *azael* had a more V-shaped girdle as in *Ngapakaldia* than the U-shape in vombatids or broad, open girdle of large diprotodontids. The reconstruction in Fig 12D shows that the ilia are less laterally extensive than in vombatids or large diprotodontids, possible evidence for a slenderer body form in *P*. *azael*.

#### Femur (Figs 13 and 14)

**Fig 13:**
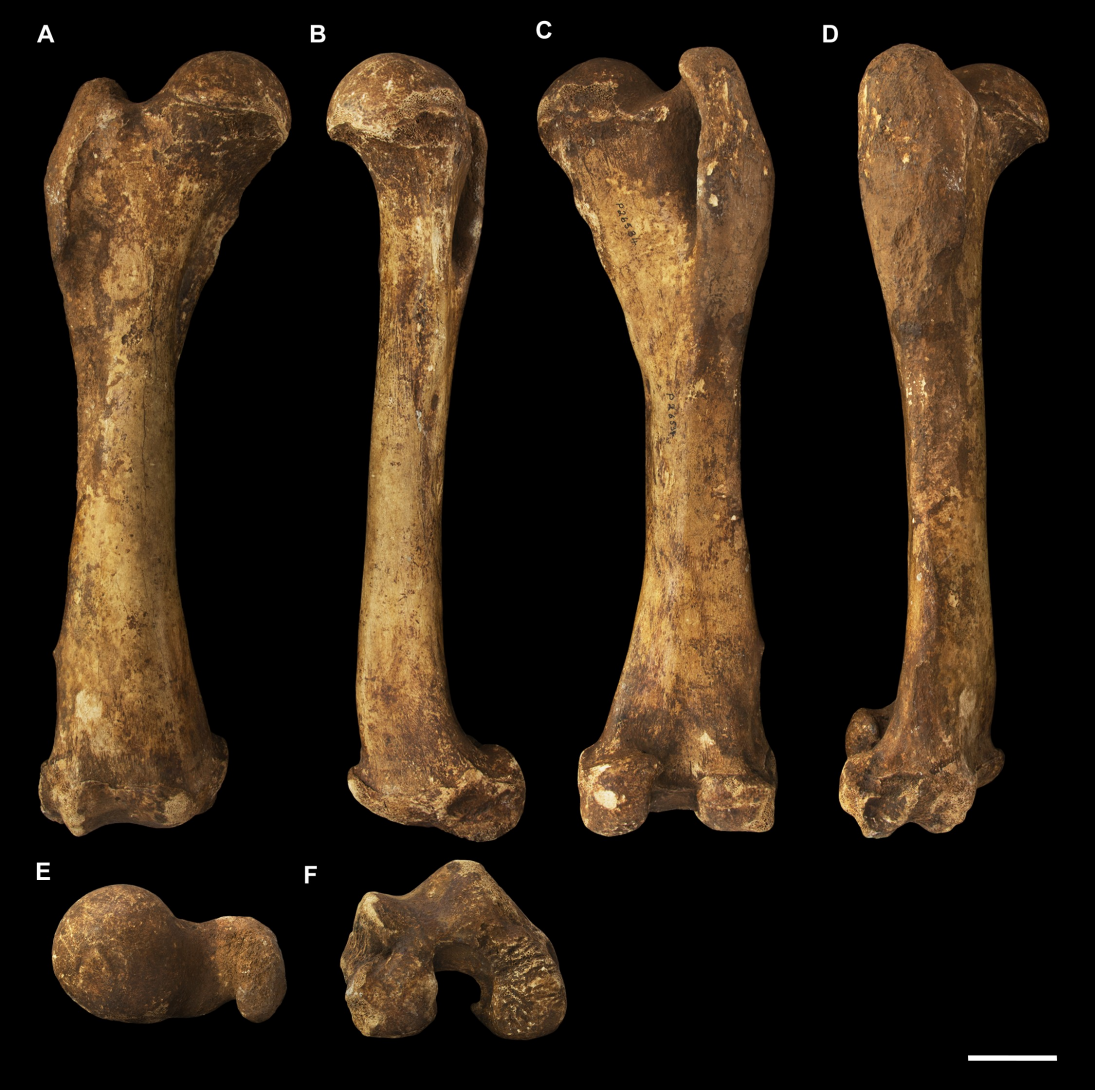
Right femur of *Palorchestes azael* NMV P26534. (A) anterior; (B) medial; (C) posterior; (D) lateral; (E) proximal; (F) distal views. Scale bar 50 mm.

**Fig 14:**
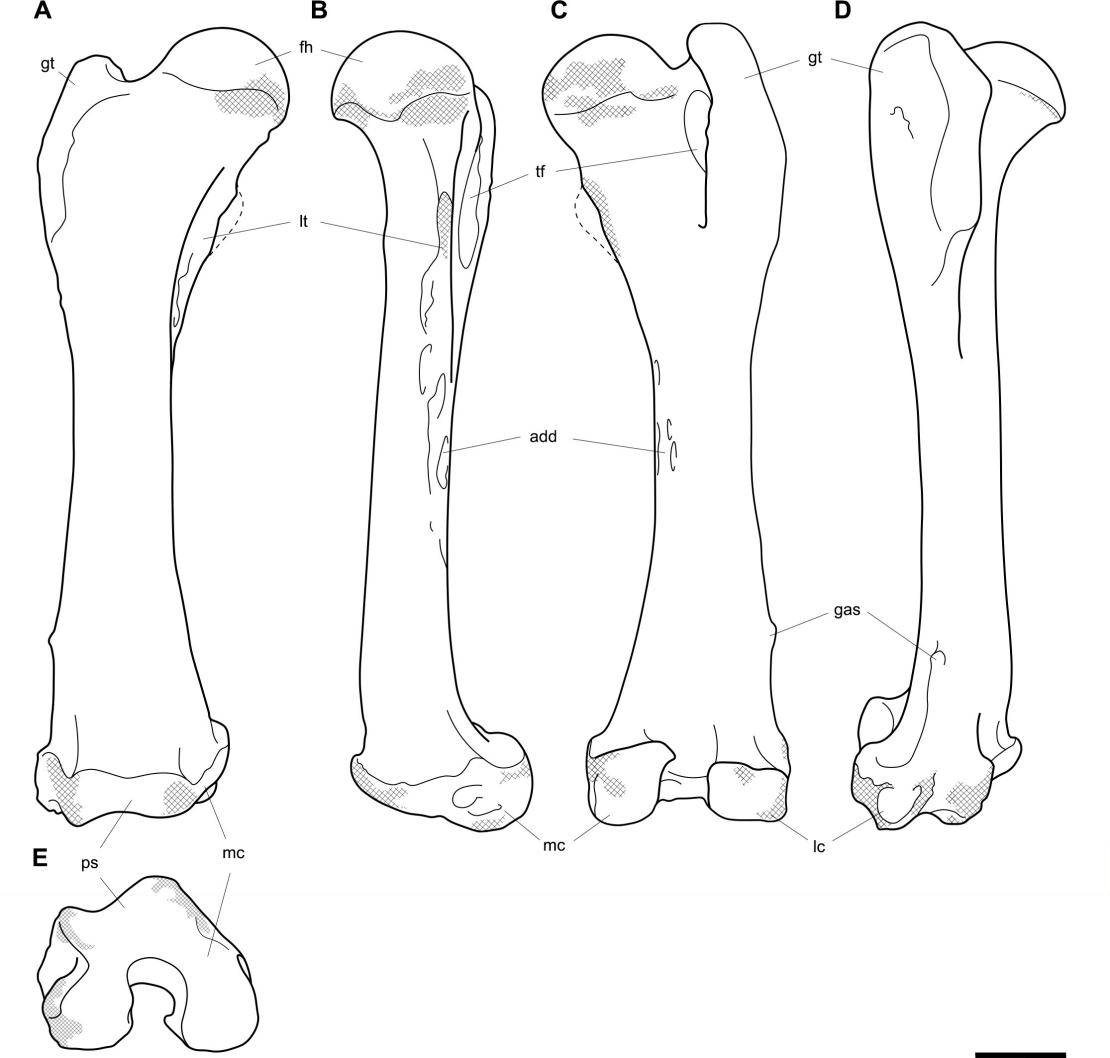
Labelled illustrations of the *Palorchestes azael* right femur NMV P26534. (A) anterior; (B) medial; (C) posterior; (D) lateral views. Hatching indicates surface damage to cortical bone, dashed lines indicate inferred bone contours. Abbreviations: **add**, insertion scars for adductor muscles; **fh**, femoral head; **gt**, greater trochanter; **gas**, origin for *m*. *gastrocnemius* lateral head; lc, lateral condyle; **lt**, lesser trochanter; **mc**, medial condyle; **ps**, patellar surface; **tf**, trochanteric fossa. Scale bar 50 mm.

The *Palorchestes azael* femur is elongate and gracile for its size, with a slender sub-cylindrical shaft and narrow epiphyses. In proportions and overall shape it strongly resembles the femur of extant *Vombatus* (see S3 Fig), but is of course significantly larger, with a weaker lesser trochanter and no gluteal tuberosity (Figs 13 and 14).

**Femoral head.** The apex of the hemispherical head lies slightly proximal to the greater trochanter when the femoral condyles are placed on a flat surface. The neck is stout and very short relative to femora of similarly-sized diprotodontids, and projects anteromedially, positioning the head such that ~30% of its bulk overhangs the anterior shaft when viewed medially. In proximal view the head is significantly deeper cranio-caudally than the diaphysis or neighbouring greater trochanter, being very similar in this respect to the anatomy of *Vombatus* and differing from other large diprotodontoids.

**Greater trochanter.** In anterior view the greater trochanter is tapered proximally as it projects strongly above the femoral neck unlike in diprotodontids. The anterolateral surface of the greater trochanteric epiphysis projects anteriorly from the shaft and curls slightly medially. In lateral view the trochanter appears expanded anteroposteriorly to give a rounded appearance, and the anterior projection of its distal portion is clearly appreciated. Posteriorly, the trochanteric fossa is very deep and relatively longer superoinferiorly than any other taxon studied. There is no discernible intertrochanteric crest or gluteal tuberosity.

**Lesser trochanter.** The lesser trochanter is an extensive plate-like flange on the proximomedial femoral shaft, arising at the femoral neck and extending distally to become a rugose insertion scar that extends to a third of the way down the entire bone length. Some damage to the proximal portion of this crest obscures its full extent, but overall the lesser trochanter appears to be similar to those of *Diprotodon* and *Zygomaturus* in that it is a broad crest rather than a distinct tubercle, especially appreciated in posterior view.

The proximal femur as a whole resembles that of *Phascolonus* more than any other vombatiform, though with key differences in *P*. *azael* including; a more acute angle between the higher greater trochanter and shorter femoral neck, more distally-extensive articular surface around all edges of the head, and a mediolaterally narrower proximal femur overall.

**Diaphysis.** The femoral diaphysis in *P*. *azael* is smooth overall. Like in other diprotodontoids there is no linea aspera as such, however the femoral shaft shows elliptical pits, grooves and muscle scarring in the area immediately distal to the lesser trochanter on the medial and posteromedial midshaft. These mark insertion areas for adductor musculature and are much more pronounced than in other vombatiforms studied. On the distolateral diaphysis a raised area bordered by a crest marks the origin for lateral head of *m*. *gastrocnemius*, similarly situated to that in *Zygomaturus* but sharper and more distinct than in the latter.

**Medial condyle.** In distal view the anteroposterior extent of the medial condyle is ~30% greater than that of the lateral (as is typical of large vombatiforms). It is mediolaterally narrower and more oblique to the sagittal axis of the bone than the lateral condyle. The medial surface of the condyle is deeply pitted for the attachment of the medial collateral ligament. In posterior view, the medial condyle presents a narrower articular surface than the lateral, with a hooked process curling in toward the bone midline as in vombatids and *Thylacoleo*, though more pronounced than both. In anterior view the distal surfaces of the medial and lateral condyles sit approximately level to a plane perpendicular to the femoral shaft, unlike the marked distal offset of the lateral condyle in large diprotodontids and *Phascolonus*.

**Lateral condyle.** The lateral condyle in distal view is anteroposteriorly shorter, mediolaterally broader and oriented more closely along the sagittal axis than its medial counterpart. In posterior view, the lateral condyle is very short proximodistally relative to the medial condyle. This contrasts both with the anatomy of *Diprotodon* and *Phascolonus* in which the lateral is taller, and with the subequal heights of the condyles in most other diprotodontids. The *Ngapakaldia* femur presents a similar condition to palorchestids, however in the latter overall both condyles appear short proximodistally relative to femoral length when compared to all other vombatiforms.

**Patellar surface.** The patellar surface in *P*. *azael* is quadrangular and only shallowly indented. In distal view the medial crest of the patellar surface only modestly protrudes from the distal femur, far less than the protrusion seen in large diprotodontids or *Phascolonus*, being more similar to extant wombats.

#### Tibia (Figs 15 and 16)

**Fig 15:**
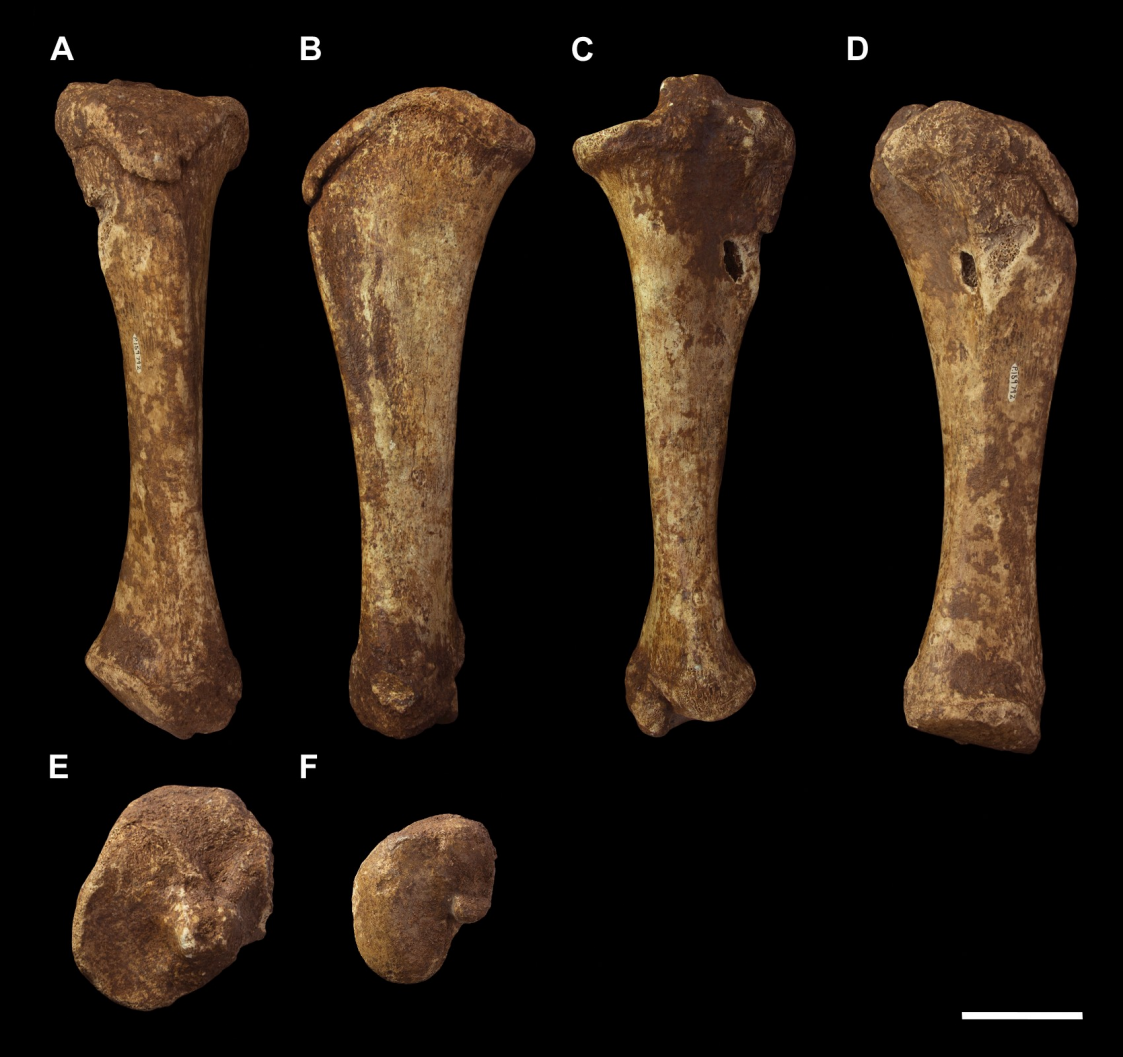
Left tibia of *Palorchestes azael* NMV P159792. (A) anterior; (B) medial; (C) posterior; (D) lateral; (E) proximal; (F) distal views. Scale bar 50 mm.

**Fig 16:**
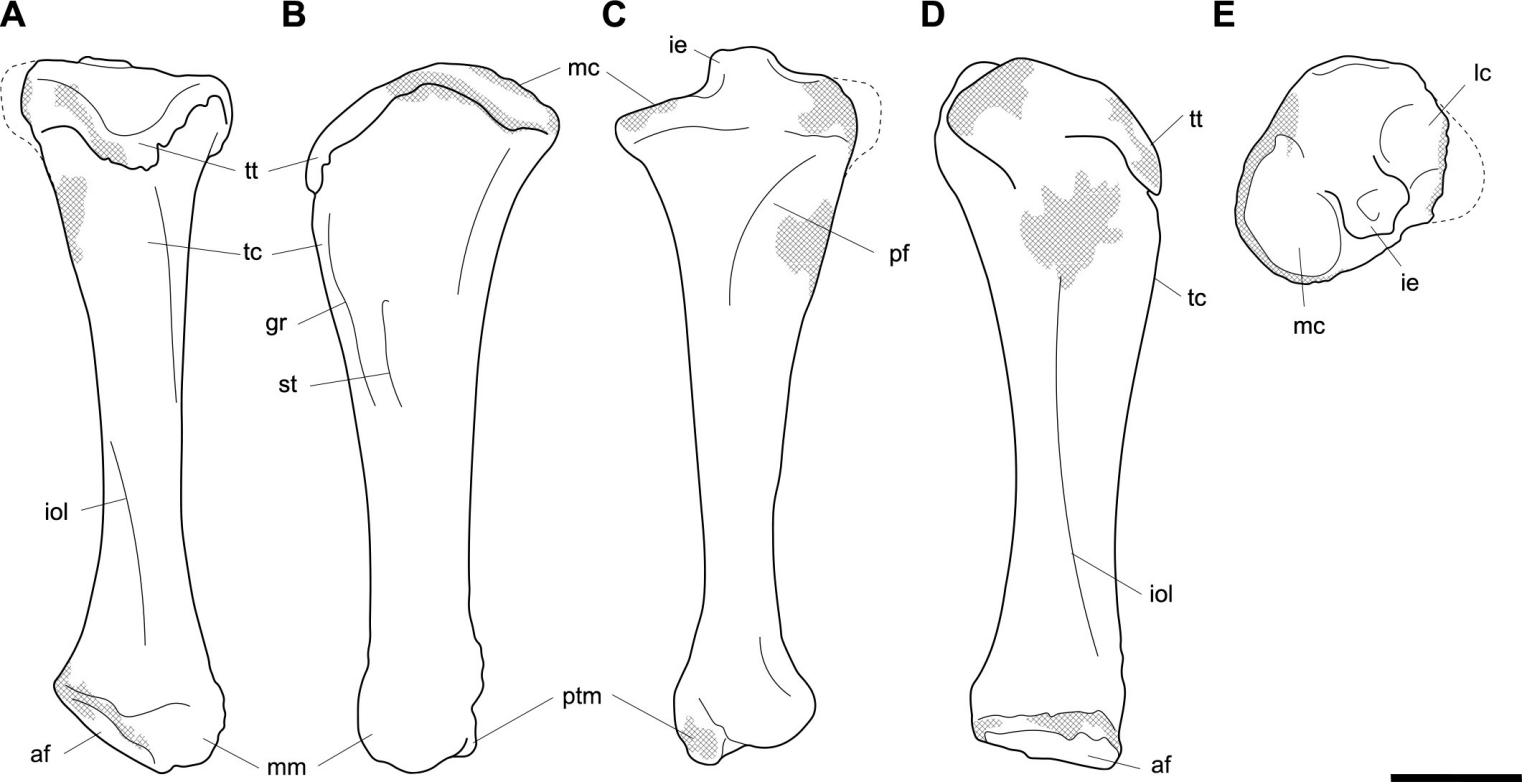
Labelled illustrations of the *Palorchestes azael* left tibia NMV P159792. (A) anterior; (B) medial; (C) posterior; (D) lateral; (E) proximal views. Hatching indicates surface damage to cortical bone, dashed lines indicate inferred bone contours. Abbreviations: **af**, astragalar facet; **gr**, insertion of *m*. *gracilis;* **ie**, intercondylar eminence; **iol**, interosseus line; **lc**, lateral condylar surface; **mc**, medial condylar surface; **mm**, medial malleolus; **pf**, popliteal fossa; **ptm**, posterior tubercle of medial malleolus; **st**, insertion of *m*. *semitendinosus;* **tc**, tibial crest; **tt**, tibial tuberosity. Scale bar 50 mm.

Other than thicker transverse dimensions along the diaphysis, the tibia of *P*. *azael* resembles that of *Vombatus* in general proportions in that it is longer relative to the epiphyses than in diprotodontids or *Phascolonus* (Figs 15 and 16).

**Proximal articulations.** The medial condylar surface is suboval, much broader in all dimensions than in *P*. *parvus* and closer in extent to the *Phascolonus* tiba. It is concave but not as deep or regular as those seen in *Diprotodon* and *Zygomaturus*. The convex lateral condylar surface is elevated on the tibial plateau relative to the medial condyle and is a near-level surface, rather than the gently posteriorly-sloped surfaces in *Phascolonus* and *Diprotodon* or the steeply sloped equivalent in *Zygomaturus*. The anterior plateau and tibial tuberosity comprise almost half of the total surface of the proximal tibia. This creates a proximal tibial surface that is expanded anteroposteriorly, approaching the vombatid condition more than that of diprotodontoids. The intercondylar eminence is a quadrangular protuberance in anterior aspect, similar to that of vombatids but more robust in proximal view. The tibial tuberosity for insertion of the patellar ligament is an inverted isosceles triangle, more elongate than in vombatids and unlike the trapezoidal diprotodontid condition. The lateralmost portion of the proximal tibia is eroded so the shape and extent of the fibular articulation in *P*. *azael* is not known. A concave popliteal fossa lies beneath the tibial plateau on the posterior side.

**Diaphysis.** The diaphysis in midsection is flattened medially and convex laterally, creating distinct anterior and posterior borders between these surfaces. The principal difference with the *Vombatus* tibia is that the tibial crest in *P*. *azael* is more proximally positioned and not as convex in mediolateral view. The line for attachment of the tibiofibular interosseus ligament lies obliquely along the lower anterolateral diaphysis, while medially the scars for *m*. *gracilis* and *m*. *semitendinosus* are strongly expressed.

**Distal articulations.** The distal articular surface is similar to that of vombatids in basic shape, contours and orientation. The astragalar facet is highly convex and continues further anterolaterally than in vombatids to provide a large laterally-facing articular surface, larger than and totally unlike the flat facet in diprotodontids. The posterior tubercle of the malleolus is a small conical process, more distinct than in diprotodontids and vombatids while being shorter and barely extending past the distal articular surface (it is absent in *Phascolonus*). Behind this lies an incised groove for the ankle and digital flexor tendons.

#### Pes (Figs 17 and 18)

**Fig 17:**
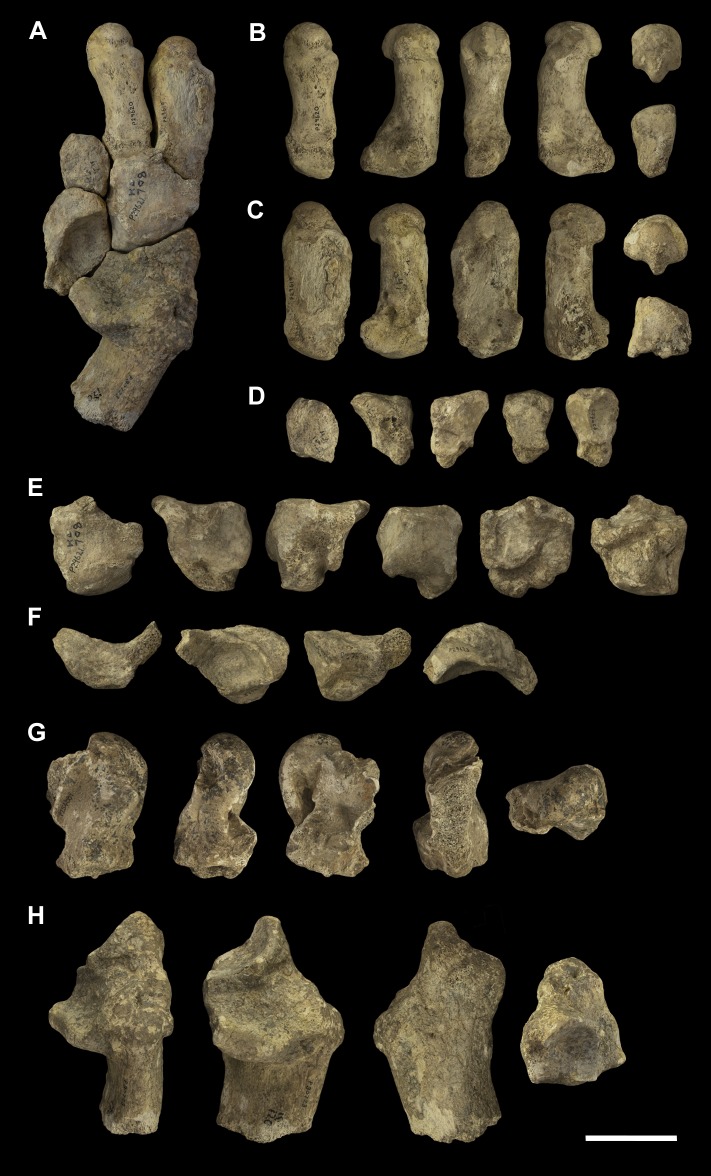
Associated pedal elements of *Palorchestes azael*. (A) articulated partial right side pes in dorsal view; (B) right side metatarsal 4 NMV P29620 in (left to right, top to bottom) dorsal, medial, plantar, lateral, distal and proximal views; (C) right side metatarsal 5 NMV P29619 in dorsal, medial, plantar, lateral, distal and proximal views; (D) right side ectocuneiform NMV P29622 in dorsal, medial, lateral, proximal and distal views; (E) right side cuboid NMV P29621 in dorsal, medial, lateral, proximal, distal and plantar views; (F) right side navicular NMV P29623 in dorsal, proximal, distal and plantar views; (G) left side astragalus fragment NMV P254089 in dorsal, medial, plantar, lateral and distal views; (H) right side calcaneus NMV P30723 in dorsal, medial, lateral and distal views. Scale bar 50 mm.

**Fig 18:**
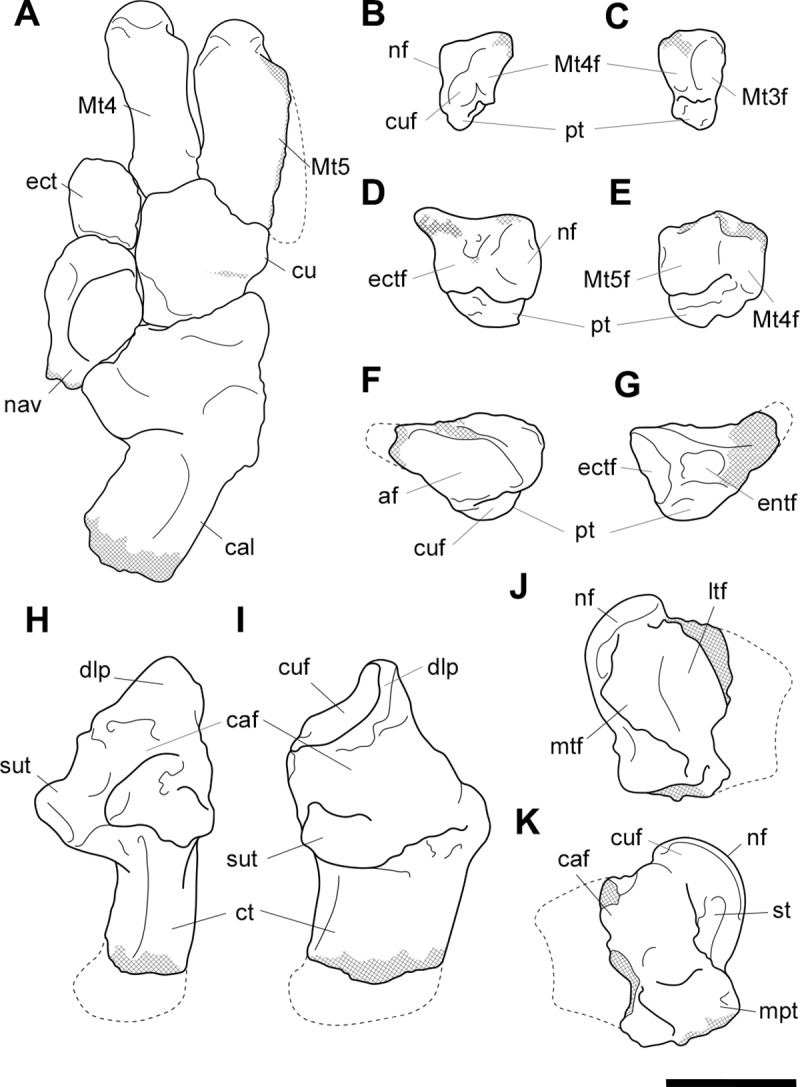
Labelled illustrations of associated pedal elements of *Palorchestes azael*. (A) articulated partial right side pes in dorsal view; right side ectocuneiform in (B) lateral and (C) distal views; right side cuboid in (D) medial and (E) distal views; right side navicular in (F) proximal and (G) distal views; right side calcaneus in (H) dorsal and (I) medial views; mirrored left side astragalus in (J) dorsal and (K) plantar views. Hatching indicates surface damage to cortical bone, dashed lines indicate inferred bone contours. Abbreviations: **af**, astragalar facet; caf, calcaneoastragalar facet; **cal**; calcaneus; **ct**, calcaneal tuber; **cu**, cuboid; **cuf**, cuboid facet; **dlp**, distolateral process; **ect**, ectocuneiform; **ectf**, ectocuneiform facet; **entf**, entocuneiform facet; **ltf**, lateral tibial facet; **mpt**, medial plantar tuberosity; **Mt4**, metatarsal 4; **Mt4f**, metatarsal 4 facet; **Mt5**, metatarsal 5; **Mt5f**, metatarsal 5 facet; **mtf**, medial tibial facet; **nav**, navicular; **nf**, navicular facet; **pt**, plantar tuberosity; **st**, sulcus tali; **sut**, sustentaculum tali. Scale bar 50 mm.

The pes of *P*. *azael* is very unlike the highly modified pedes of other giant vombatiforms (Figs 17 and 18).

**Calcaneus.** The sustentaculum is better developed and more distinct than in *Zygomaturus* and *Diprotodon*, presenting a steeply sloped astragalar facet laterally. The sustentaculum has a curved articular facet on its superior edge and is dorsoventrally thickened compared to the calcaneus of *Ng*. *tedfordi*. The calcaneal tuber is comparatively straight. The posteriormost part of the tuber is eroded so the total length and extent to which it curves medially is not known. However, it is clear that the tuber is not the narrow sigmoid-shaped structure of *Zygomaturus* and instead appears to resemble the morphology seen in *Nimbadon*, *Thylacoleo* and *Ng*. *tedfordi*. The distolateral process is very pointed in dorsal view. The cuboid facet is a near-spherical concavity, more dorsally-facing than in most diprotodontids, bearing the closest resemblance to that of *Neohelos* and *Ng*. *tedfordi*. The plantar surface is pitted and rugose, especially towards the distal end.

**Astragalus.** The astragalus is damaged posterolaterally, missing the lateral part of the tibial facet and the entire fibular facet–their contours are estimated in Fig 18B and 18C. As such, it is not possible to discern the extent of the facet for the pyramidalis sesamoid on its dorsal surface. Overall the remaining morphology strongly resembles *Ngapakaldia tedfordi* astragali in proportions and joint surface shape, the principal difference besides size being that it is dorsoventrally deeper as in *Nimbadon*. Like in *Ng*. *tedfordi* and *Nimbadon*, the tibial facet dorsally is very concave, much more than in *Zygomaturus*. In distal view, the navicular facet is ventromedially sloped relative to the tibial surface, unlike the flatter facets in *Zygomaturus* or *Nimbadon*. In ventral view, the calcaneal surface is much smaller and less anteriorly extensive than in *Zygomaturus*, as well as having a much better developed medial plantar tuberosity (*sensu* Munson [24]). The navicular surface sits separate and ventral to the calcaneal surface on the plantar aspect and has a distinct facet for articulation with the cuboid. Anterolaterally, the sustentacular facet is very convex, dipping into a deeply concave fossa (much more concave than in *Zygomaturus*) before the plantar tuberosity arises medially. The sulcus tali is similar to that of *Zygomaturus* though is more deeply excavated.

**Navicular.** The navicular is robust and relatively dorsoventrally expanded compared to those of *P*. *parvus* and *Ng*. *tedfordi*. The cuboid facet is relatively larger than in *Ng*. *tedfordi* and quite concave. The facet for the ectocuneiform is broad and only slightly concave. This articulation appears relatively larger in *P*. *azael* than in *Nimbadon*. Its plantar tuberosity is not as developed as in derived diprotodontids or *Thylacoleo*.

**Cuboid.** The cuboid has a well-developed plantar tuberosity, anterior to which runs a deep, narrow sulcus for the *m*. *peroneus longus* tendon as it approaches the first digit on the plantar surface of the pes. This sulcus is the deepest we observed among our comparative taxa.

**Ectocuneiform.** In dorsal view the ectocuneiform is much larger relative to the cuboid than in *Ng*. *tedfordi* and *Zygomaturus*, but not as large as in *Thylacoleo*. Distally, it presents a concave ovoid medial facet for metatarsal 3, two-thirds the height of its distal face, the other ventral third being made up of a well-developed plantar tuberosity. Immediately lateral and oriented 45° to this ovoid facet is a sub-equally sized flat facet for the proximomedial surface of metatarsal 4. The similarity in size between these facets may indicate that *P*. *azael* had less reduced second and third digits than *P*. *parvus*, or alternatively may reflect a greater degree of articulation of metatarsal 4 with the cuboid and thus reduction in articulation with the ectocuneiform. On the lateral edge of the ectocuneiform is a smaller surface for articulation with the cuboid, while its proximal face is almost completely occupied by a smooth, concave facet for the navicular.

**Metatarsals.** The metatarsals of *P*. *azael* are thick and robust, though more elongate than the smaller *P*. *parvus* species. Both exhibit large median keels on the plantar aspect of their heads, suggesting the presence of flexor sesamoids.

**Metatarsal 4.** The fourth metatarsal of *P*. *azael* has a smoothly convex, triangular cuboid facet, relatively more dorsoventrally elongate than in *P*. *parvus* and lacking the horizontal sulcus on its articular surface seen in the latter. The proximomedial facet for the ectocuneiform is also more dorsoventrally extensive and more sagittally oriented than in other members of its genus, instead resembling the condition in *Nimbadon*. There is no accompanying facet to suggest articulation with the third metatarsal, unlike in *P*. *parvus*. Proximolaterally, the facet for metatarsal 5 is slightly lunate and proportionally broader than in *P*. *parvus*, but not as dorsoventrally extensive, terminating above the volar tip of the metatarsal. The metatarsal head appears hemispherical in dorsal view, while in ventral view its large central keel is flattened proximally and projects strongly toward the plantar surface, flanked by smaller medial and lateral keels. In distal view the head is domed and symmetrical, lacking the lateral cant seen in *P*. *parvus*.

**Metatarsal 5.** Metatarsal 5 is a stout bone. The proximal tuberosity is damaged, leaving the bone almost cylindrical in shape with flattened ventral and medial shaft surfaces. However, it is likely that *P*. *azael* had an extensive proximal tuberosity here based on its occurrence in both *P*. *parvus* AM F58870 and other diprotodontoids, and the shape of the eroded metatarsal surface. Proximally the cuboid facet is smoothly convex and meets the facet for metatarsal 4 medially. There is a deep notch on the proximoventral edge of the bone, bounded medially by a ventral projection of the facet for metatarsal 4 –this would have provided passage for digital flexor tendons. The head appears hemispherical in dorsal view, the extensive median keel only becoming visible in distal view. The medial keel is flattened and reduced, and the lateral keel is sloped and reduced almost to absence.

### Palorchestes parvus De Vis 1895

#### Referred material

Measurements for all referred material below are provided in S1 Table.

**AM F58870.** Associated partial skeleton including: premaxilla fragment with incisor alveoli matches syntype QMF789 (Woods [3], Fig 4; Trusler [36], Fig 5.19K); left humerus; right os coxa (acetabulum and partial ilium); left femur (two fragments; proximal two-fifths, some damage to posterior femoral neck and greater trochanter; distal epiphysis); left tibia (proximal two-fifths fused in flexion to distal femur); partial left manus (missing phalanges 5 and all carpals except for trapezium); partial left pes (missing calcaneus and astragalus). Collected by G. Hope from ‘cave at Wee Jasper, Punch Bowl Hill, below and left of Signature Cave’, NSW in 1977.

**NMV P159792.** Associated partial skeleton including: left tibia (shaft broken and repaired above distal epiphysis, some cortical bone missing, referred based on morphological match with tibia from AM F58870); left ulna (proximal fragment containing humeral and radial articulations with some damage to articular surfaces); right radius (proximal two-thirds with damage to the capitular rim); left radius (distal fragment with well-preserved epiphysis). These are smaller, yet mature elements registered as NMV P159792, leading us to conclude that *P*. *azael* and *P*. *parvus* species are both represented, with a total MNI of 3 across all NMV P159792 specimens (two *P*. *azael* and one *P*. *parvus*). Collected by F. Spry from Buchan Caves (probably Foul Air Cave), VIC in 1907.

#### Humerus (Figs 19 and 20)

**Fig 19:**
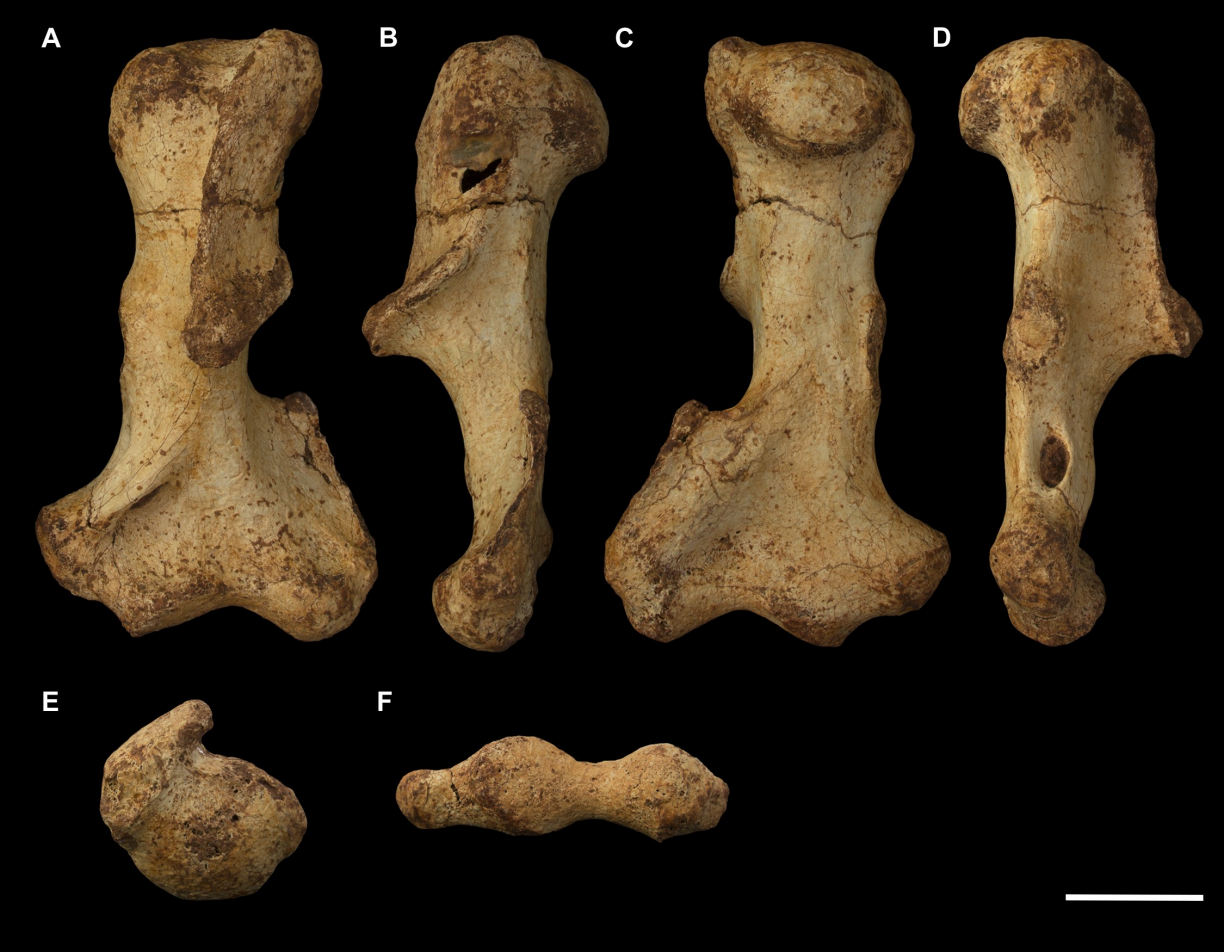
Left humerus of *Palorchestes parvus* AM F58870. (A) anterior; (B) lateral; (C) posterior; (D) medial; (E) proximal; (F) distal views. Scale bar 50 mm.

**Fig 20:**
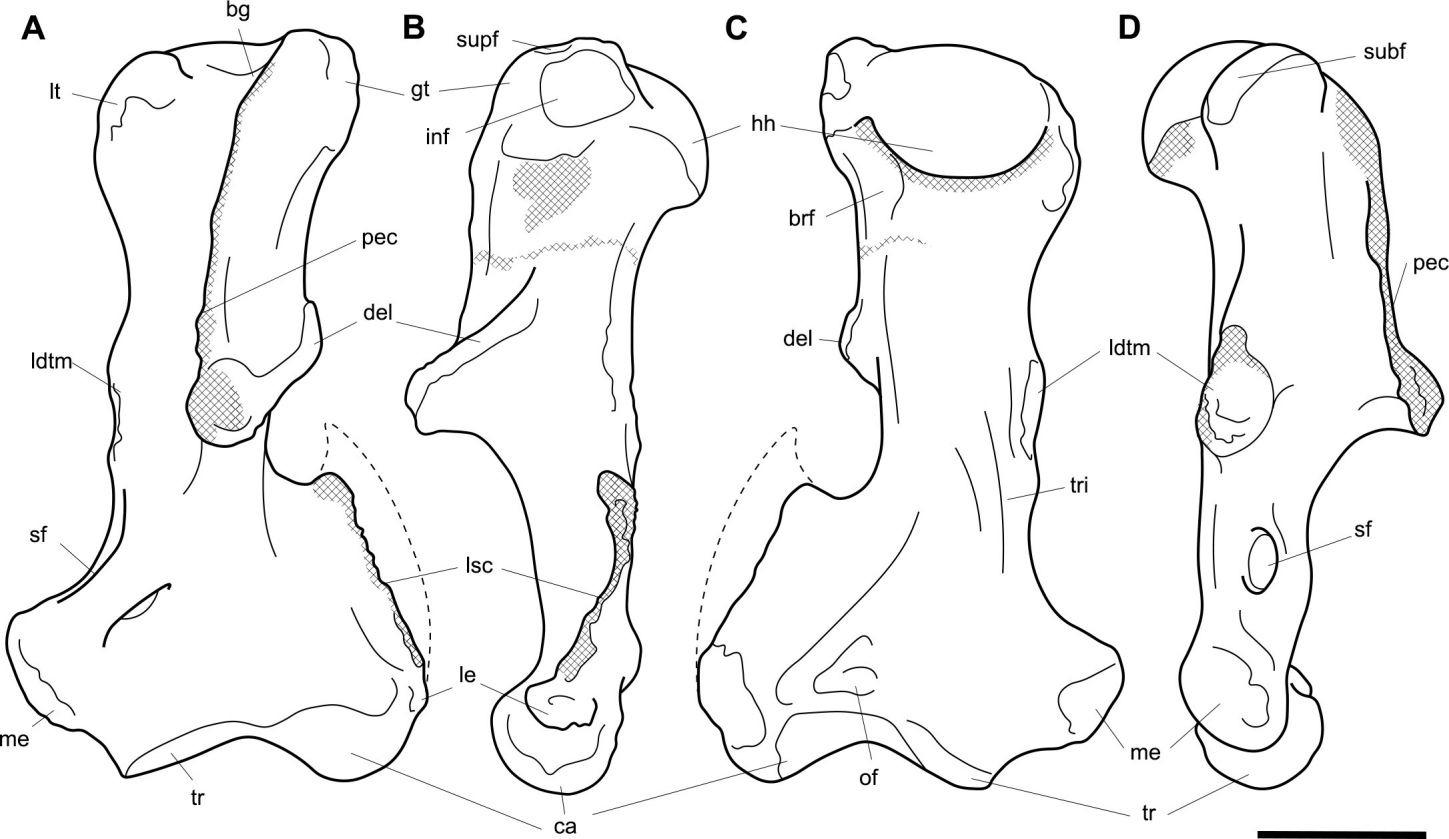
Labelled illustrations of the *Palorchestes parvus* left humerus AM F58870. (A) anterior; (B) lateral; (C) posterior; (D) lateral views. Hatching indicates surface damage to cortical bone, dashed lines indicate inferred bone contours. Abbreviations: **bg**, bicipital groove; **brf**, fossa for *m*. *brachialis* origin; **ca**, capitulum; **del**, deltoid insertion; **eta**, origin for *m*. *epitrochleoanconeus*; **gt**, greater tubercle; **hh**, humeral head; **inf**, fossa for insertion of *m*. *infraspinatus;* **ldtm**, insertion for *mm*. *latissimus dorsi* and *teres major;* **le**, lateral epicondyle; **lsc**, lateral supracondylar crest; **lt**, lesser tubercle; **me**, medial epicondyle; **of**, olecranon fossa; pec, pectoral crest; **subf**, fossa for insertion of *m*. *subscapularis;* **supf**, fossa for insertion of *m*. *supraspinatus;* **sf**, supracondylar foramen; **tr**, trochlea; **tri**, origin for humeral heads of *m*. *triceps brachii*. Scale bar 50 mm.

The humerus of *P*. *parvus* is wombat-like overall, with stout and robust proportions and thick muscle attachment crests (Figs 19 and 20).

**Head.** The humeral head is sub-hemispherical and larger relative to its surrounding proximal humeral structures than in other palorchestids. The posteroinferior tip of the head appears to ‘beak’ less from the posterior diaphysis in lateral view than in *P*. *azael*.

**Greater tubercle.** The greater tubercle in *P*. *parvus* projects slightly proximally to the humeral head and has two major muscle attachment fossae; the fossa for insertion of *m*. *supraspinatus*, which is a flattened ovoid shape on the proximal surface of the tubercle, and the broad, posterolaterally-oriented fossa for *m*. *infraspinatus*. These fossae resemble those of *P*. *azael* in relative proportions to the tubercle and to each other. In proximal view, the greater tubercle is slightly more anteriorly positioned than in *P*. *azael*, but not as anterior as in *Propalorchestes*. In lateral view the greater tubercle is extensive anteroposteriorly, to the same degree as in *P*. *azael*.

**Lesser tubercle.** In anterior view, the apex of the lesser tubercle sits just inferior to the humeral head, the highest relative position of the palorchestids. In this view it has a rounded medial margin which appears more inflated than any other taxon studied. In medial view, the elongate attachment scar for *m*. *subscapularis* lies obliquely along the posterior margin of the tubercle, similar to that of *Propalorchestes*. Viewed proximally, the lesser tubercle resembles that of *P*. *azael* in all respects except in the contours of the bicipital groove, which in *P*. *parvus* is more concave/less flattened and positioned nearer to the midline of the bone.

**Deltopectoral crest.** The deltopectoral crest in *P*. *parvus* is thick and well developed, being similarly shaped to vombatids overall but differing in some key respects. The pectoral insertion crest runs subvertically from the greater tubercle down the approximate midline of the humeral shaft, and along its entire length the pectoral crest sharply overhangs the bicipital groove medially. This is unlike vombatids where the entire deltopectoral crest is offset laterally from the humeral shaft, the posterior part overhanging the brachialis fossa rather than the anteromedial part overhanging the bicipital groove. The medial overhang of this crest begins at the proximal metaphysis and is clearly visible in proximal view–this overhang is more proximal than in *P*. *azael*, though the crest becomes less developed than the latter as it passes distally. Like vombatids, there is an oblique crest coursing inferoanteriorly from the lateral shaft to converge medially with the pectoral crest at its distal tip. The lateral edge of this oblique crest is likely to have been the attachment site for the scapular part of *m*. *deltoideus*. Just inferior to the greater tubercle, a faint ridge runs vertically for a short distance on the anterolateral aspect of the pectoral crest–this probably marks the insertion point for the clavicular deltoid. This insertion ridge in *P*. *parvus* is weaker than the vombatid condition but is totally absent in *P*. *azael*, suggesting the clavicular deltoid played a reduced role in *Palorchestes* species (and especially in *P*. *azael*) relative to wombats. In *P*. *parvus* the terminal point on the deltopectoral crest is swollen into a distinct tuberosity. Vombatids lack a tuberosity here, and the shared distal end of these crests is more laterally positioned. *Propalorchestes* has a similar tuberosity in this position, although it is not associated with the scapular deltoid insertion which is situated proximally.

**Tuberosity for *mm*. *teres major* and *latissimus dorsi*.** The insertion scar for the combined tendon of *mm*. *teres major* and *latissimus dorsi* is a lachrymiform fossa situated on the medial humeral shaft overhanging its posterior border slightly more than halfway down its length. In relative terms it is both the largest and most distally-positioned of these tuberosities among all the palorchestids.

**Diaphysis.** In anterior and lateral views, the *P*. *parvus* humeral shaft appears straight overall though somewhat distorted by the crests and tubercles along its length. Like *P*. *azael*, in *P*. *parvus* attachment scars for *m*. *triceps brachii* lie on the posterior shaft surface, though the fossa for the origin of *m*. *brachialis* below the lateral lip of the humeral head is less pronounced. Inferiorly, a deep triangular olecranon fossa lies above the posterior articular surface of the capitulum, facilitating at least some extension of the elbow. The posteroinferior diaphysis lacks the marked rugosity for *m*. *epitrochleoanconeus* seen in *P*. *azael*.

**Lateral epicondyle.** The lateral epicondyle projects slightly from the upper rim of the capitulum when viewed anteriorly. In lateral view, it appears pitted and rugose, resembling that of *Propalorchestes* in its shape and extent. From this epicondyle, the lateral supracondylar crest extends proximally as a thin sheet of bone, its lateral margin damaged but the remnant characteristic vombatiform hook still visible.

**Trochlea.** In *P*. *parvus* the trochlea faces inferiorly but due to slight convexity both mediolaterally and anteroposteriorly its articular surface is just visible in anterior view. This represents an intermediate morphology between the curved, wombat-like condition in *Propalorchestes* and the flat trochlea of *P*. *azael*. Like the latter species, in anterior view the trochlea and capitulum project equally distally, though the angle between the two processes is less acute in *P*. *parvus*. In distal view the trochlea is ovoid and a little larger than the capitulum in dorsoventral depth. It is aligned with the capitulum in the dorsal (coronal) plane of the humerus.

**Supracondylar foramen.** The supracondylar foramen in *P*. *parvus* is deep and ovoid, spanned by a bridge broader and more robust than in other palorchestids.

**Medial epicondyle.** The medial epicondyle projects strongly medially from the vertical axis of the humeral shaft. In anterior view, the overall shape and relative extent the *P*. *parvus* medial epicondyle resembles that of *Propalorchestes*, being less wide and rounded than in *P*. *azael*. In medial view the *P*. *parvus* epicondyle is bulbous and thick anteroposteriorly.

#### Ulna (Figs 21 and 22)

**Fig 21:**
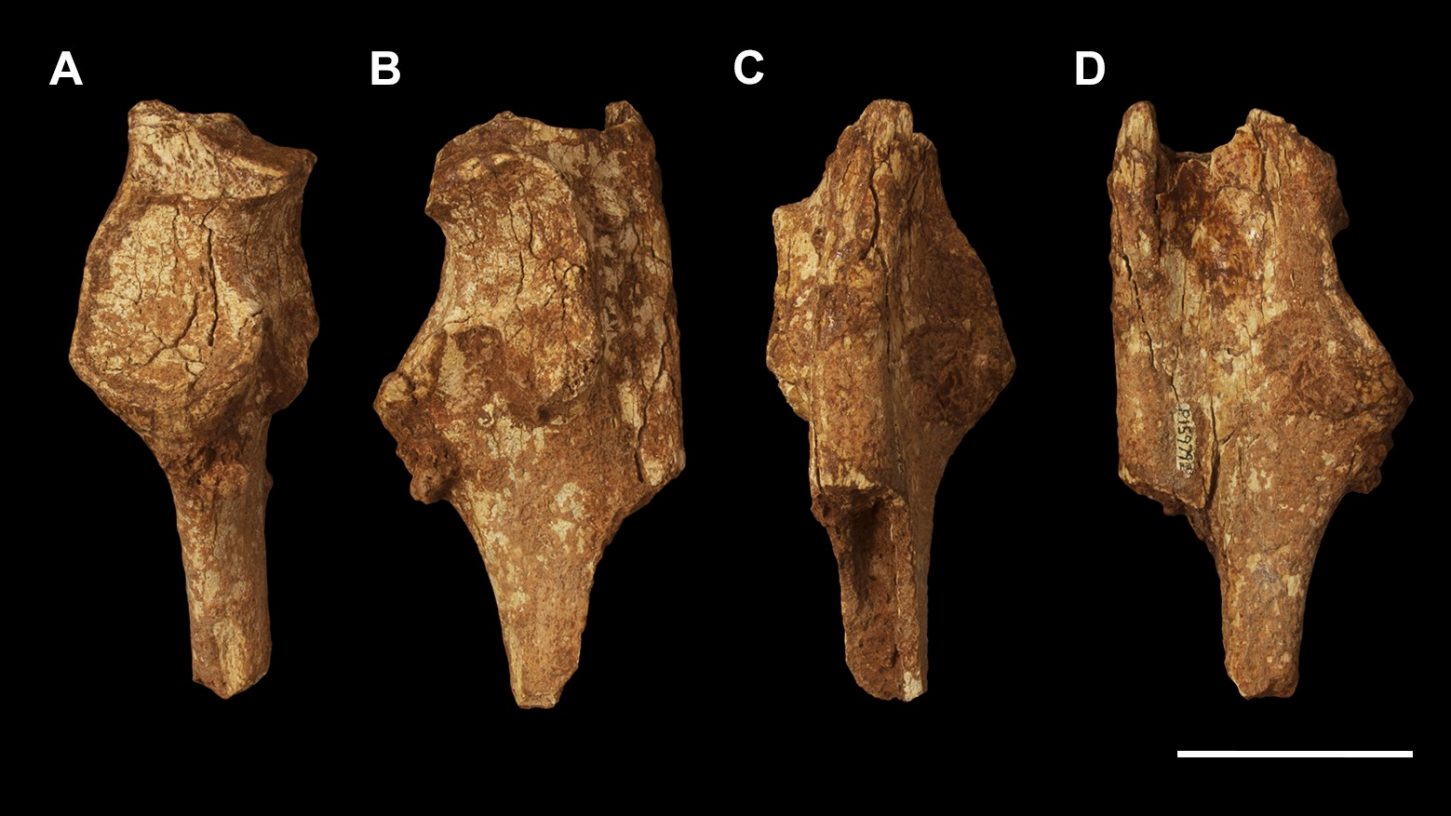
Left ulna fragment of *Palorchestes parvus* NMV P159792. (A) anterior; (B) lateral; (C) posterior; (D) medial views. Scale bar 50 mm.

**Fig 22:**
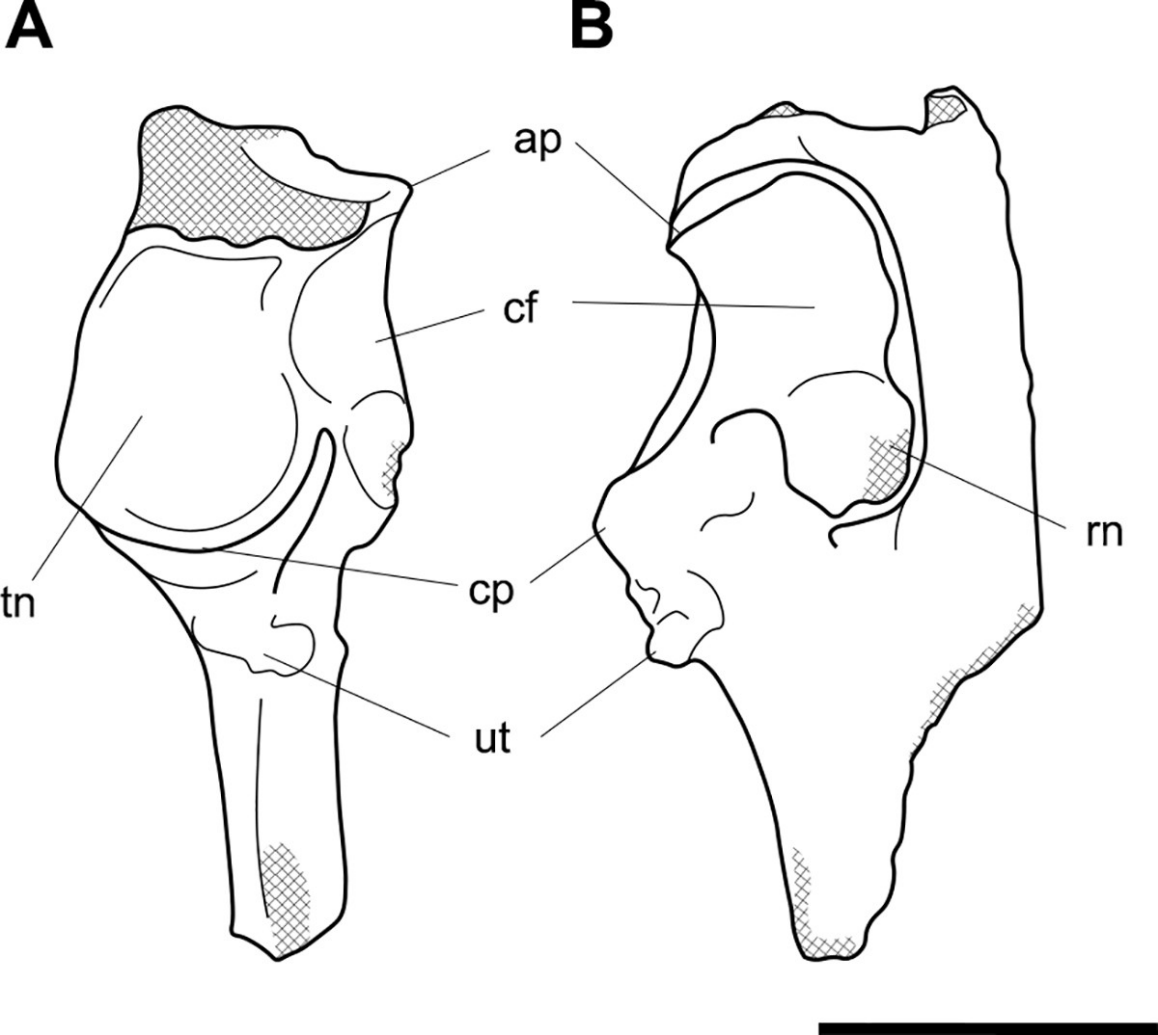
Labelled illustrations of *Palorchestes parvus* left ulna fragment NMV P159792. (A) anterior; (B) lateral views. Hatching indicates surface damage to cortical bone. Abbreviations: **ap**, anconeal process; **cf**, capitular facet; **cp**, coronoid process; **rn**, radial notch; **tn**, trochlear notch; **ut**, ulnar tuberosity. Scale bar 50 mm.

The only ulna specimens are fragments preserving the proximal articulations and short adjacent portion of the olecranon (Figs 21 and 22). The preserved morphology suggests that the olecranon would have been consistent with other palorchestids, aligned with the diaphysis and not posteriorly deflected as in diprotodontids, and not medially deflected as in *Propalorchestes*, instead resembling the morphology in *P*. *azael*. The trochlear surface is broad and subcircular like that of *Vombatus* and *Ng*. *bonythoni*, not narrowed and elongate as in *P*. *azael*. The tallest point of the anconeal process is much more anteriorly oriented than the laterally-deflected equivalent in *P*. *azael*, and creates a C-shaped trochlear notch in medial view similar to those of *Propalorchestes* and extant wombats. The coronoid process is relatively low and proximodistally thick like in *P*. *azael*, not thin and tall as in *Vombatus*. The radial notch and facet for the humeral capitulum lie in the same dorsal plane, more in line with one another than in *P*. *azael* where the radial notch is more dorsally offset than the capitular facet. The radial notch is large, approximately half the proximodistal length of the adjacent trochlear surface. In *P*. *azael* the radial notch is relatively shorter. The pit for attachment of the annular ligament between the trochlear and radial notches is shallower in *P*. *parvus* than in *P*. *azael*. The ulnar tuberosity is small and less rugose than in *P*. *azael*, and in a more proximolateral position. The posterior border of the ulnar shaft immediately dorsal to the coronoid process is narrower mediolaterally than in *P*. *azael*, indicating a less robust bone overall.

#### Radius (Figs 23 and 24)

**Fig 23:**
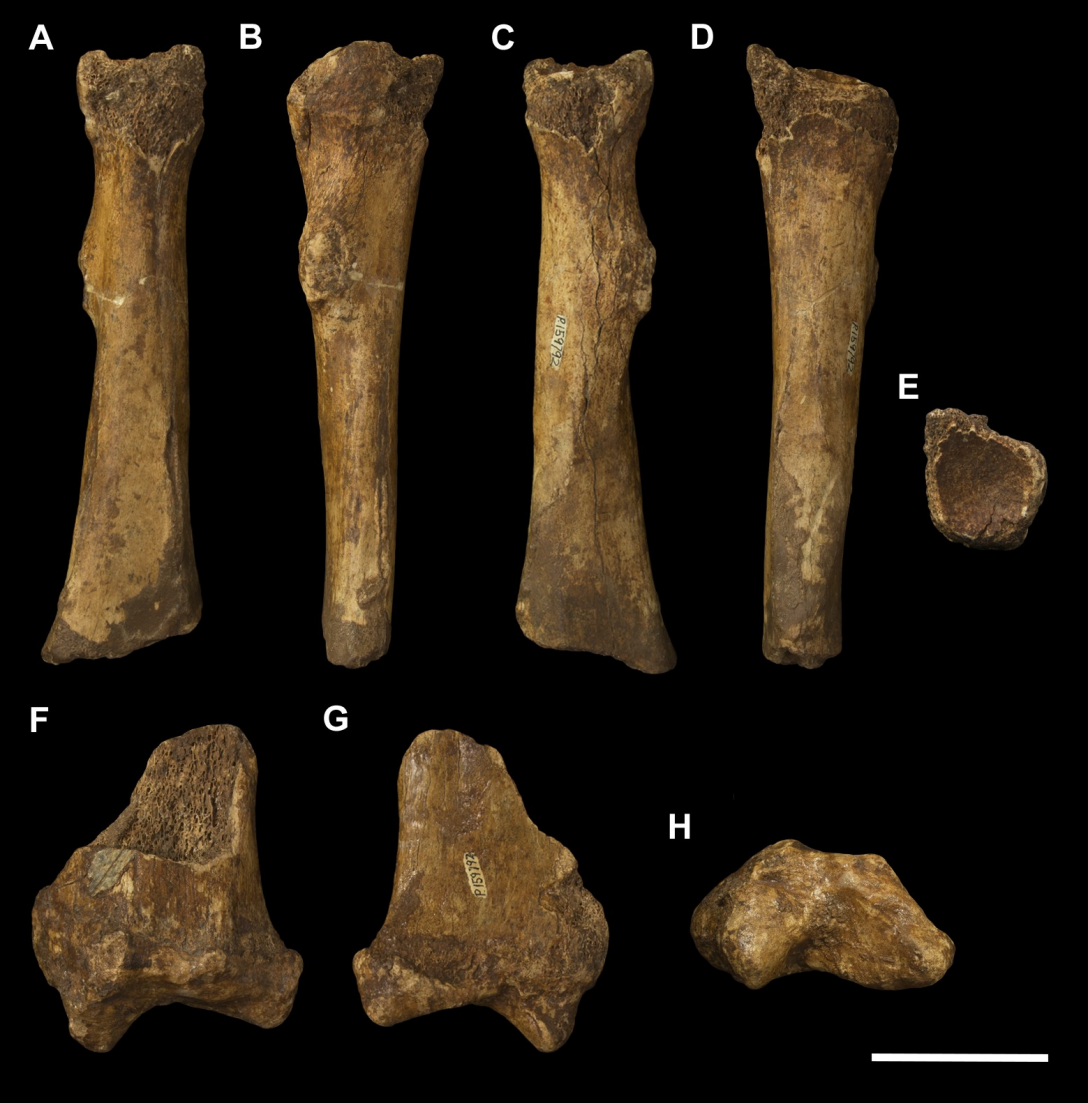
Radius fragments of *Palorchestes parvus* NMV P159792. Right side radius in (A) dorsal; (B) medial; (C) ventral; (D) lateral; (E) proximal views. Distal left side radius in (F) dorsal; (G) ventral; (H) distal views. Scale bar 50 mm.

**Fig 24:**
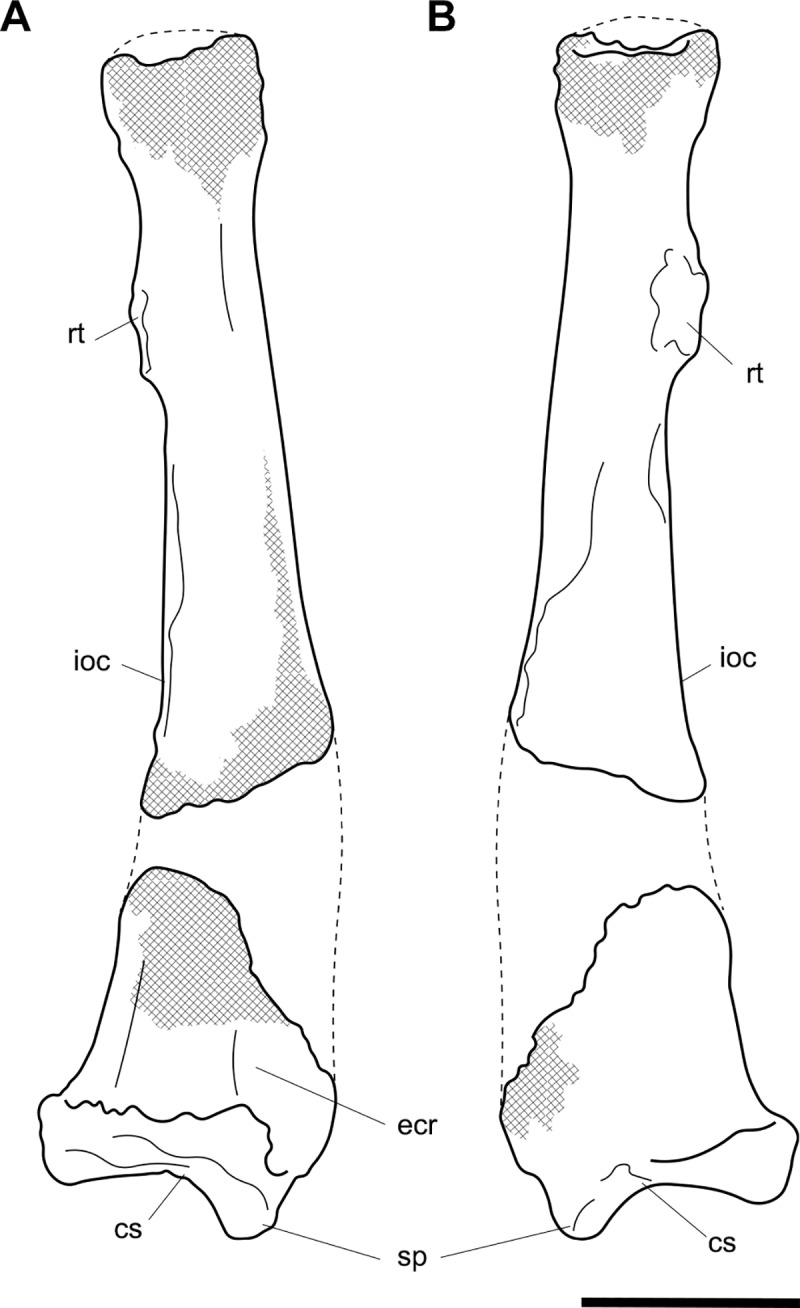
Labelled illustration of estimated reconstruction of *Palorchestes parvus* right radius. (A) dorsal; (B) ventral views. Length estimated based on *Palorchestes azael* radius (Fig 7), left side distal fragment mirrored and scaled to fit. Hatching indicates surface damage to cortical bone, dashed lines indicate inferred bone contours. Abbreviations: **cs**, carpal surface; **ecr**, notch for tendon of *m*. *extensor carpi radialis;* **ioc**, interosseous crest; **rt**, radial tuberosity; **sp**, styloid process. Scale bar 50 mm.

No complete radius is known for *P*. *parvus* (Figs 23 and 24). The overlapping morphology of the proximal fragment is similar to that of *P*. *azael*, the principal difference being the medial and lateral borders are less curved, indicating a straighter radius overall. The radial tuberosity for insertion of *m*. *biceps brachii* is further distal to the head than in *P*. *azael*. The distal fragment is trapezoidal in profile like in *P*. *azael*, though in *P*. *parvus* it is more dorsoventrally flattened. The radiocarpal surface most resembles *Ngapakaldia* in form, in particular the relative size of the styloid and extent to which it is inset from the medial border of the epiphysis. The flattened dorsal surface is notched for *mm*. *extensor carpi radialis* and *extensor digitorum communis* tendons.

#### Manus (Figs 25 and 26)

**Fig 25:**
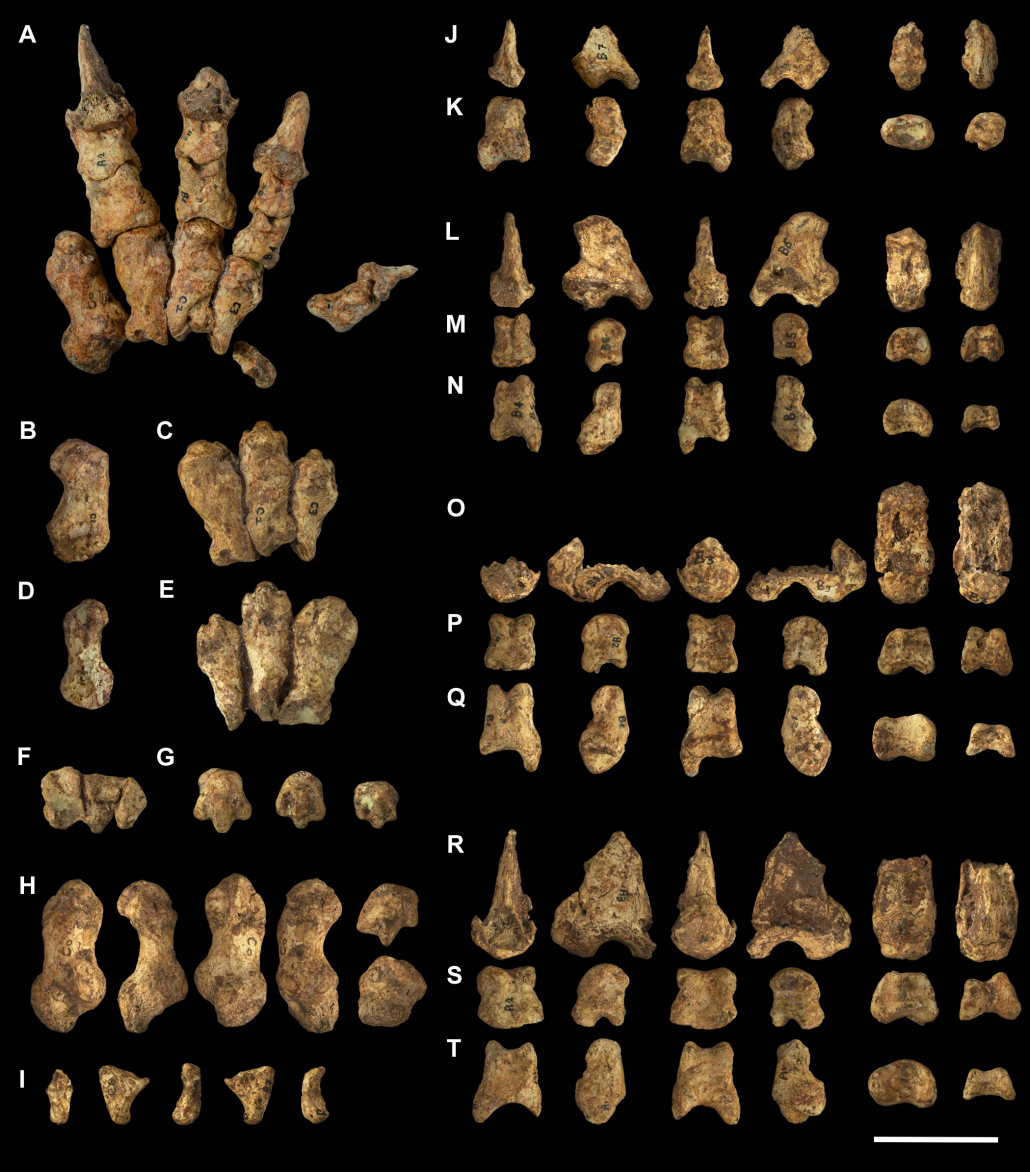
Associated partial left manus of *Palorchestes parvus* AM F58870. (A) articulated manus in dorsal view; (B) metacarpal 4 in (left to right) lateral view; (C) metacarpals 4–2 (cemented together) in dorsal view; (D) metacarpal 2 in medial view; (E) metacarpals 2–4 in palmar view; (F) metacarpals 4–2 in proximal view; (G) metacarpals 4–2 in distal view; (H) metacarpal 5 in (left to right, top to bottom) dorsal, lateral, plantar, medial, distal and proximal views; (I) trapezium in dorsal, proximal, lateral, distal and medial views; (J-K) digit 1/pollex ungual and proximal phalanges in dorsal, lateral, plantar, medial, proximal and distal views; (L-N) digit 2 ungual, intermediate and proximal phalanges in dorsal, lateral, plantar, medial, proximal and distal views; (O-Q) digit 3 ungual, intermediate and proximal phalanges in dorsal, lateral, plantar, medial, proximal and distal views; (R-T) digit 4 ungual, intermediate and proximal phalanges in dorsal, lateral, plantar, medial, proximal and distal views. Scale bar 50 mm.

**Fig 26:**
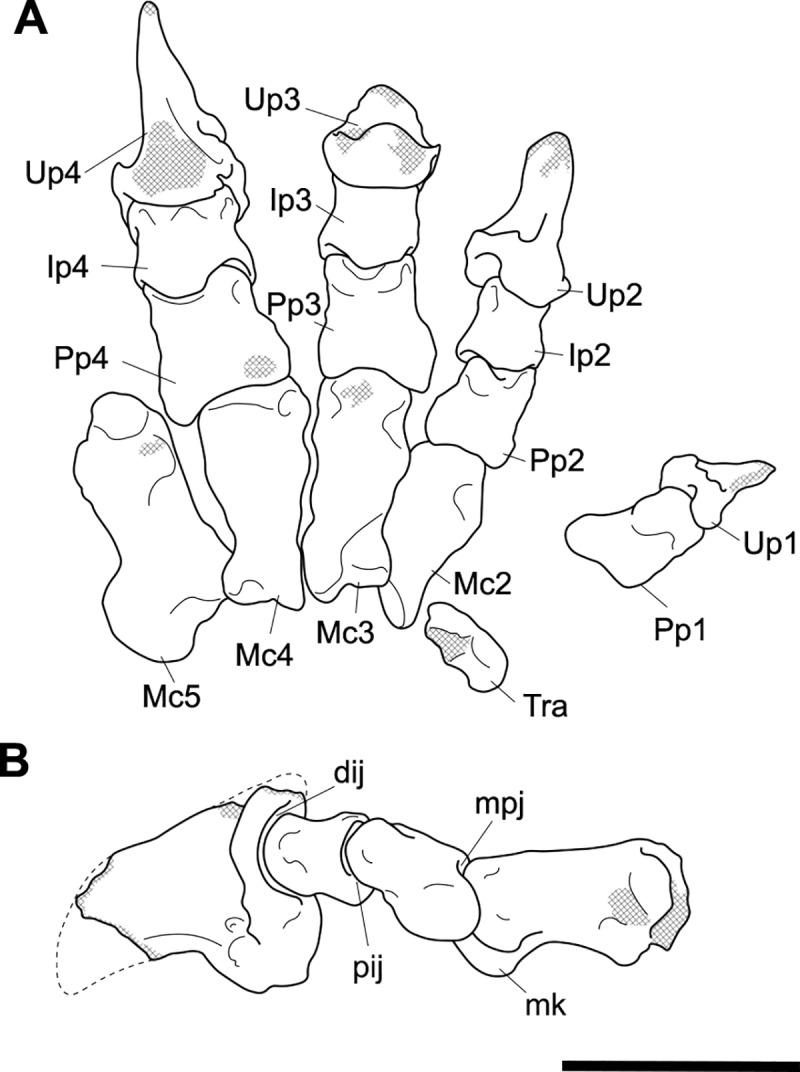
Labelled illustration of articulated *Palorchestes parvus* left manus AM F58870. (A) articulated manus in dorsal view; (B) digit 4 in lateral view. Hatching indicates surface damage to cortical bone, dashed lines indicate inferred bone contours. Abbreviations: **dij**, distal interphalangeal joint; **Ip1-4**, intermediate phalanges 1–4; **Mc2-5**, metacarpals 2–5; **mk**, median keel; **mpj**, metacarpophalangeal joint; **pij**, proximal interphalangeal joint; **Pp1-4**, proximal phalanges 1–4; **Tra**, trapezium; **Up1-4**, ungual phalanges 1–4. Scale bar 50 mm.

The *P*. *parvus* manus is represented by an associated set of stout metacarpals and phalanges (Figs 25 and 26).

**Trapezium.** The trapezium is very similar to that of *Ngapakaldia bonythoni* overall, being only a little larger and more proximodistally compressed. It has a saddle-shaped facet for the first metacarpal on its palmar-distal face with slightly flatter contours than the former species. A shallow concave facet for the trapezoid lies on its lateral face (though this is somewhat eroded), and a well-defined facet for the palmar process of the scaphoid sits on its proximal surface. The orientation of its articulations indicates the pollex would have been abducted to a similar degree to that seen in *Ngapakaldia* and much more than in *Zygomaturus* or extant wombats.

**Metacarpals.** The metacarpals overall are stouter and more distally expanded than in *Vombatus*, zygomaturines and *Ngapakaldia*. They are compact, with contoured margins for adjacent contact over most of their length. Distal condyles are sub-hemispherical in lateral view, with pronounced keels on the palmar surface whose contours are visible even in dorsal view. The metacarpals are deeply pitted distolaterally and distomedially for attachment of collateral ligaments.

**Metacarpal 2.** The second metacarpal is considerably smaller than its neighbours, with a sharply tapering proximal end resembling that of *Ngapakaldia*. The metacarpal presents a short, oblique dorsolateral facet for the magnum/capitatum and convex medial facet where the trapezium cups its medial edge.

**Metacarpal 3.** The third metacarpal has a triangular proximal facet for the magnum/capitatum, which in dorsal view has a chevron-shaped notch as in *Ngapakaldia*, and a broad, flat facet laterally for metacarpal 4.

**Metacarpal 4.** The fourth metacarpal is the longest and most robust as is common in marsupials. Proximally, the facet for the unciform/hamatum is triangular, with an oblique median groove. On the proximolateral surface, two ovoid tubercles separated by a sulcus are present for articulation with metacarpal 5.

**Metacarpal 5**. Metacarpal 5 has a strongly developed tuberosity bulging proximolaterally from the proximal quarter of the bone and extending beyond the unciform/hamatum facet. Due to the overall shape of the metacarpal, this tuberosity is more exaggerated in relation to the shaft than in *Zygomaturus* or *Phascolonus* but does not approach the enormous equivalent in *Diprotodon*. There is a lunate dorsomedial facet for articulation with the fourth metacarpal. As in *Ngapakaldia*, the facet for the lateral process of the unciform does not extend as far laterally as it does in *Vombatus*.

**Proximal phalanges.** There are well-developed pits and crests for collateral ligaments of the digital joints throughout the hand. The proximal phalanges are short, broad and flattened dorsoventrally with dished-in dorsal surfaces. Their bases are markedly asymmetrical in dorsal view, with deeply concave metacarpal sockets buttressed strongly on one side (4 and 1 laterally, 2 and 3 medially). This asymmetry is presumably to resist different prevailing forces acting on each metacarpophalangeal joint. This buttressing pattern was also noted in *Nimbadon* and may be indicative of similar range of motion (ROM) and loading regimes at these joints; however, the *P*. *parvus* proximal phalanges lack the palmar tuberosities of *Nimbadon*, so similar overall use of the manus seems unlikely. The heads are wide, each with two flared distal condyles tilted ventrally. These create joint surfaces necessitating a flexed posture for the proximal interphalangeal joints. Between these articular surfaces there are deep median incisurae to accommodate the corresponding dorsomedian processes of the intermediate phalanges. The proximal phalanx of the pollex is slightly different to the other digits, being more gracile overall, with a more compact and bulbous distal end allowing greater ROM in extension.

**Intermediate phalanges.** The intermediate phalanges are short and squat, similar in overall proportions to those of vombatids and *Diprotodon*, with broader proximal than distal ends in dorsal view. In proximal view the joint surfaces approach isosceles trapezoids in shape. Their proximal articular surfaces are divided by dorsomedian processes into two deeply excavated fossae to accommodate the condyles of the preceding phalanges. These fossae are subequal in size, with 3 and 4 having slightly larger lateral surfaces. There is slight axial torsion in the orientation of the proximal and distal articular ends, especially in the fourth digit where the distal end is medially rotated relative to the proximal. The bicondylar distal articulations of these intermediate phalanges are most strongly congruent with their unguals in a flexed distal interphalangeal joint position. These condyles have a more reduced dorsal articular surface than in *Nimbadon* or *Neohelos*, indicating a reduced extension ROM. The intermediate phalanges of the manus can be distinguished from those of the pes by the shape of the proximoventral border; manual phalanges have a distinct median excavation between the condylar fossae, making a “W” shaped ventral contour. Pedal intermediate phalanges 4 and 5 are much flatter, with little to no concavity.

**Ungual phalanges.** No individual ungual from the manus of AM F58870 is complete and undamaged, but the intact morphology can be understood in composite across the specimens available. In shape and proportion they agree with unguals of *P*. *azael*, being only slightly smaller than the smallest example from that species (see S1 Table). Across the digits the unguals differ greatly in size, with the pollex being smallest and fourth ungual the largest.

#### Os coxa (Figs 27 and 28)

**Fig 27:**
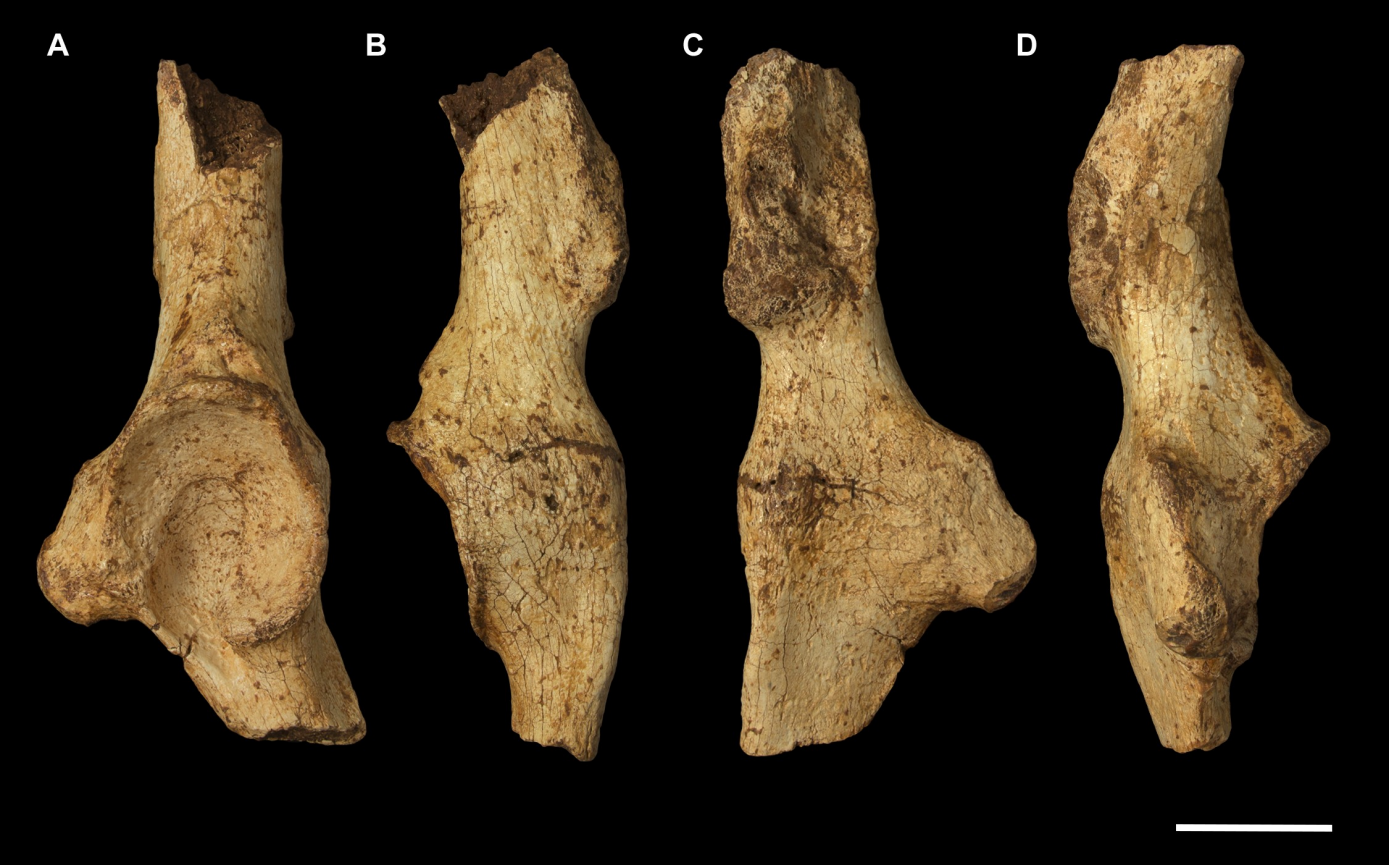
Left os coxa fragment of *Palorchestes parvus* AM F58870. (A) lateral; (B) dorsal; (C) medial; (D) ventral views. Scale bar 50 mm.

**Fig 28:**
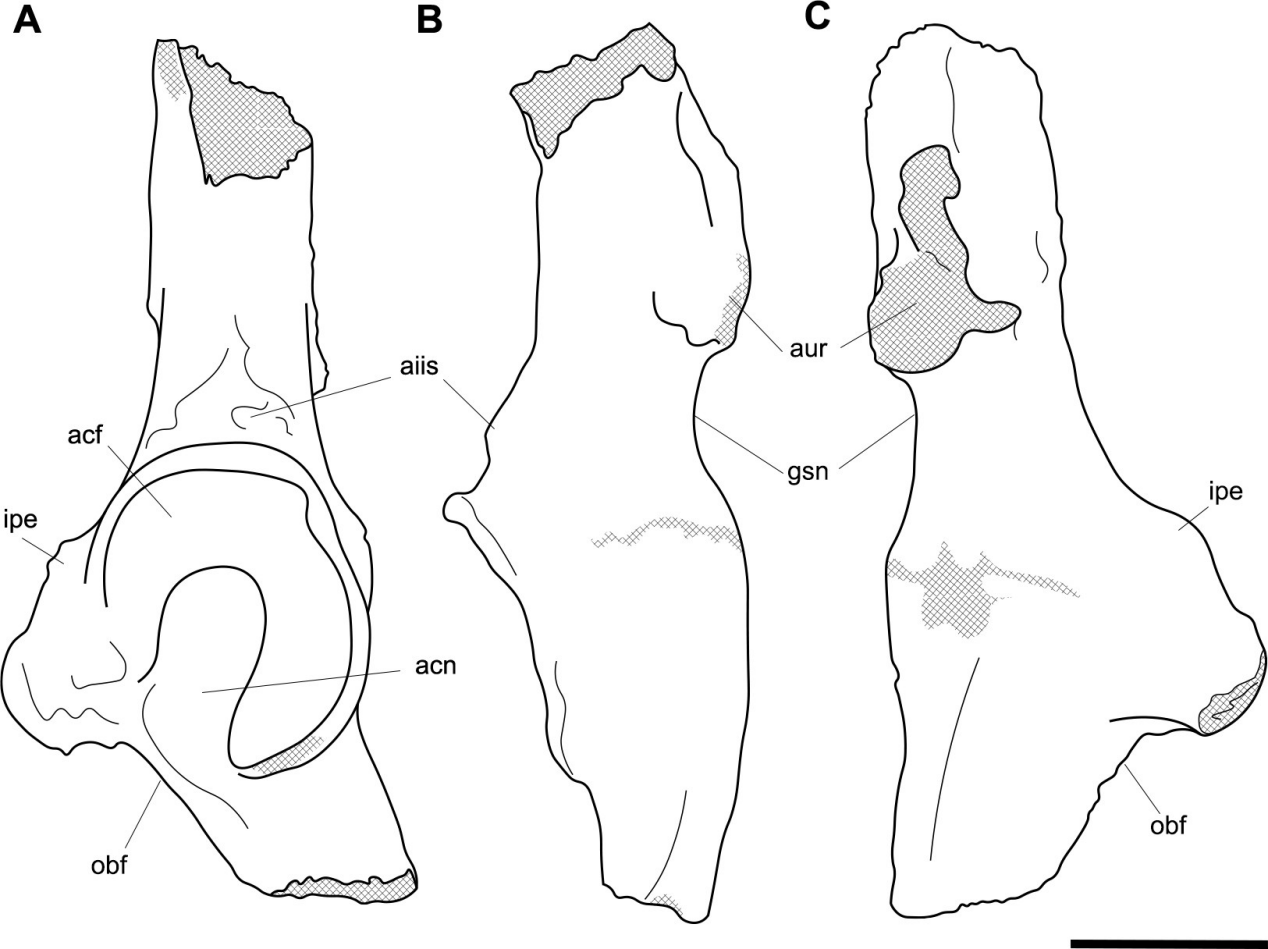
Labelled illustrations of *Palorchestes parvus* left os coxa fragment AM F58870. (A) lateral; (B) dorsal; (C) medial views. Hatching indicates surface damage to cortical bone. Abbreviations: **acf**, acetabular fossa; **acn**, acetabular notch; **aiis**, anterior inferior iliac spine; **aur**, auricular surface; **gsn**, greater sciatic notch; **ipe**, iliopectineal eminence; **obf**, obturator foramen. Scale bar 50 mm.

The single os coxa specimen for *P*. *parvus* is missing the iliac blade and most of the pubis and ischium (Figs 27 and 28). The remaining element appears similar to that of *P*. *azael*, with the same positioning of the ilium, ischium and pubis relative to the acetabulum in lateral view, though the acetabulum may be slightly smaller relative to its surrounding morphology in *P*. *parvus*.

**Ischial body.** The proximal-most part of the ischial body is preserved in *P*. *parvus*. It is thicker and more triangular in section than in *P*. *azael*, but its full extent and shape of the ischial tuberosity is not known.

**Acetabulum.** The acetabulum in *P*. *parvus* is slightly more circular than in *P*. *azael*, with relatively less overhang and narrower articular surface area posterior to the acetabular notch.

**Anterior inferior iliac spine.** The anterior inferior iliac spine is a broad, triangular scar for origin of *m*. *rectus femoris*. It is relatively wider and more deeply pitted than in *P*. *azael* and lies higher on the iliac body relative to the acetabular rim, similar to that of *Neohelos* though more rugose.

**Iliopectineal eminence.** The iliopectineal eminence is more diffuse and flattened compared to that of *P*. *azael*.

**Auricular surface.** The auricular surface in *P*. *parvus* is relatively more superoinferiorly extensive and more irregular than in *P*. *azael*, lacking the distinct facets seen in the latter. It is bordered posteriorly by a more open greater sciatic notch than in the larger species.

#### Femur (Figs 29, 30 and 31)

**Fig 29:**
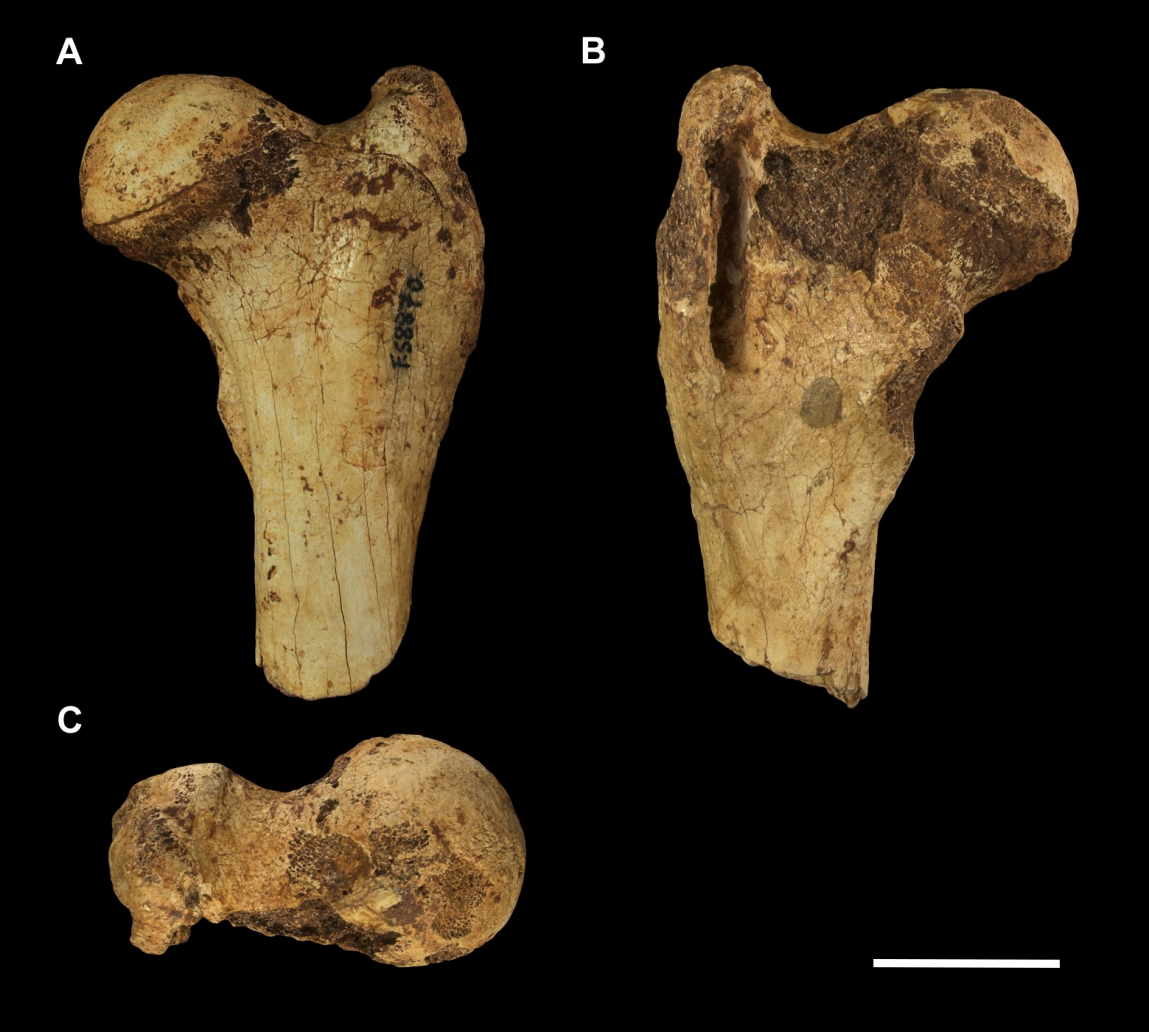
Proximal left femur of *Palorchestes parvus* AM F58870. (A) anterior view; (B) posterior view; (C) proximal view. Scale bar 50 mm.

**Fig 30:**
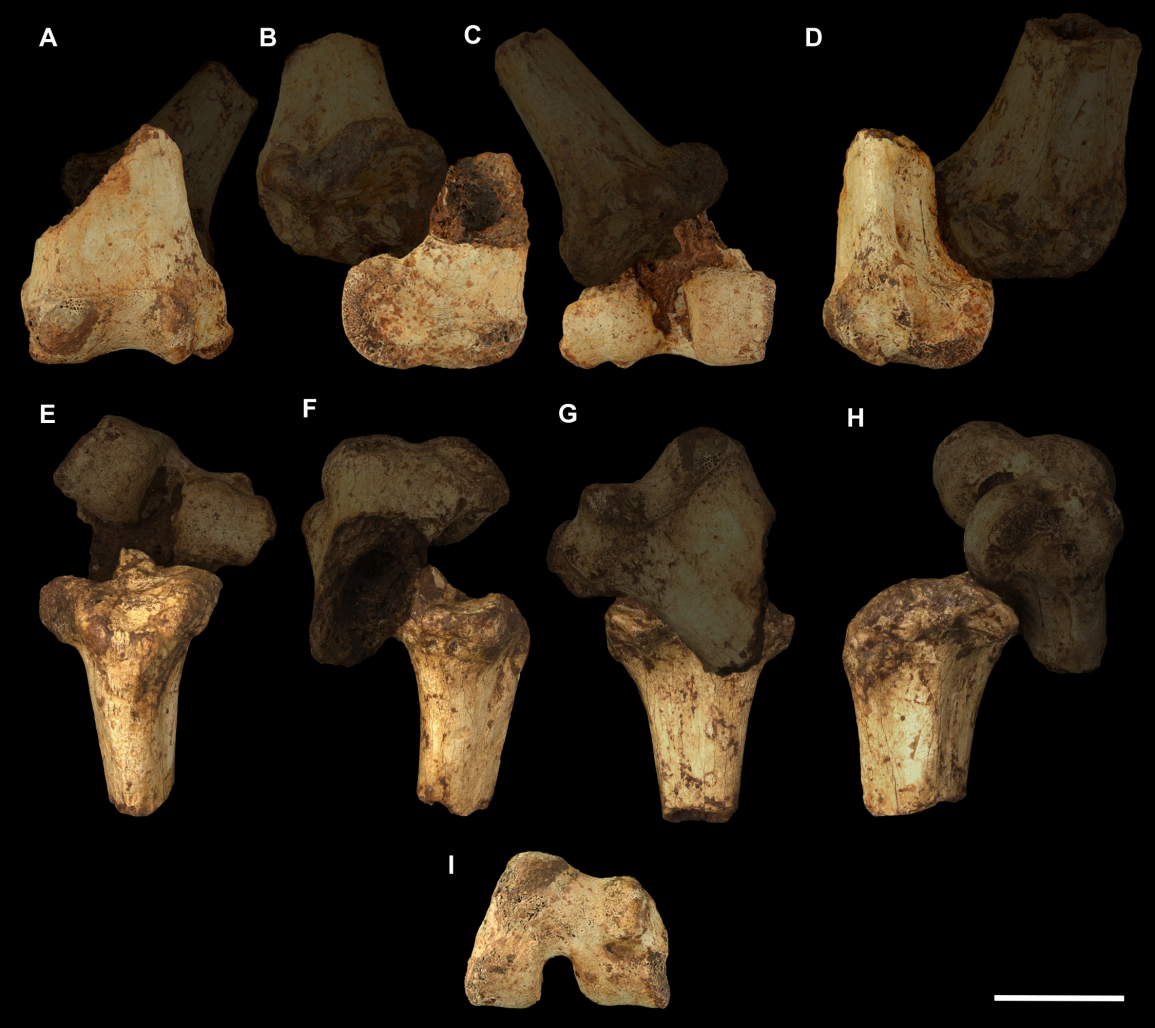
Cemented fragments of distal left femur and proximal left tibia of *Palorchestes parvus* AM F58870. Distal left femur fragment photographed in orthogonal views with tibia fragment greyed out (A-D, I) and proximal left tibia fragment photographed in orthogonal views with femur fragment greyed out (E-H) in (A, E) anterior; (B, F) medial; (C, G) posterior; (D, H) lateral; (I) distal views. Scale bar 50 mm.

**Fig 31:**
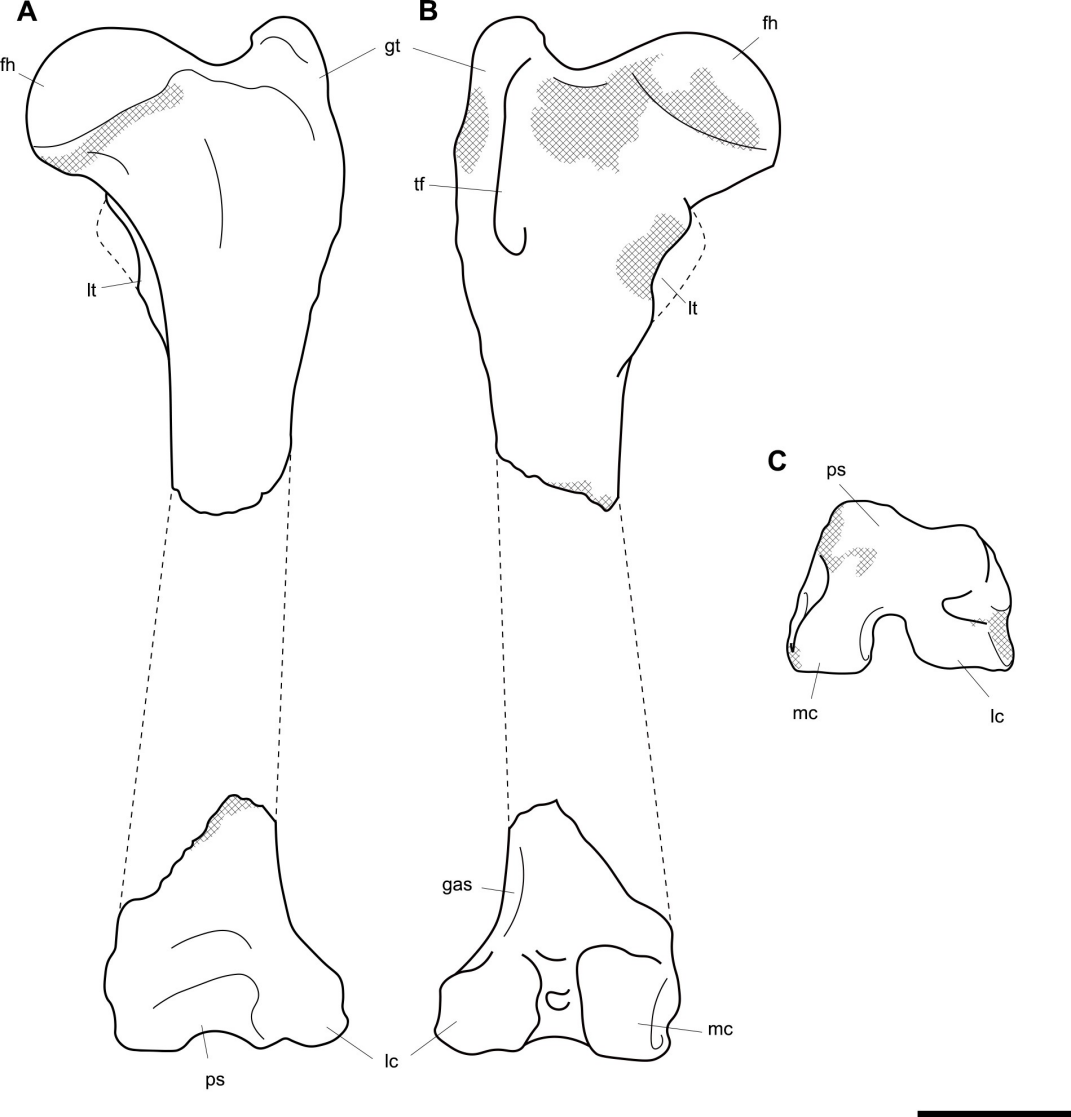
Labelled illustrations of estimated reconstruction of *Palorchestes parvus* left femur AM F58870. (A) anterior; (B) posterior; (C) distal views. Length estimated based on *Palorchestes azael* femur (Fig 13). Hatching indicates surface damage to cortical bone, dashed lines indicate inferred bone contours. Abbreviations: **fh**, femoral head; **gas**, origin for *m*. *gastrocnemius* lateral head; **gt**, greater trochanter; **lc**, lateral condyle; **lt**, lesser trochanter; **mc**, medial condyle; **ps**, patellar surface; **tf**, trochanteric fossa. Scale bar 50 mm.

The femur of *P*. *parvus* is represented by associated proximal and distal ends, missing the central femoral diaphysis (Figs 29 and 30). For this reason, the length of the intact bone can only be estimated (378 mm predicted based on ratio of proximal breadth to length in *P*. *azael*, see Fig 31). In shape and proportions it strongly resembles the *P*. *azael* femur, albeit with a broader distal epiphysis relative to proximal breadth (0.86 in *P*. *parvus*, 0.80 in *P*. *azael*).

**Femoral head.** The femoral head is hemispherical, with approximately equal articular surface available anteriorly, medially and posteriorly. The head is anteriorly offset from the diaphyseal axis as in the larger *P*. *azael*, but to a slightly lesser extent. The neck is short and does not project superomedially from the proximal shaft to the degree seen in the long-necked femora of *Zygomaturus* or *Diprotodon*, or to a lesser extent *Neohelos*.

**Greater trochanter.** The greater trochanter is proximally tapered and anteroposteriorly deep. In anterior view its rugose muscle attachments are superolaterally located as in *P*. *azael*, more so than the lower and more anterior trochanter of *Phascolonus*. Unlike the femur of *P*. *azael*, the *P*. *parvus* greater trochanter lacks the distinct swelling at the distal end where the epiphysis merges with the anterolateral femoral shaft and extends a shorter distance down the shaft. Posteriorly, the trochanteric fossa is elongate and deep as in *P*. *azael*.

**Lesser trochanter.** The main tuber of the lesser trochanter is eroded so its full medial extent is unknown, however it appears to have been more robust and its flange more developed than *P*. *azael*, while proximodistally a little shorter. At the base of the lesser trochanter on the medial diaphysis there is a rugose area immediately proximal to the break in the shaft.

**Diaphysis.** The bulk of the femoral diaphysis is missing, leaving only the proximal and distal portions, however from the section profiles it appears similar in shape to that of *P*. *azael*. Notable on the distolateral diaphysis is a deep and narrow, proximodistally elongate muscle scar for the lateral head of *m*. *gastrocnemius*. In contrast to *P*. *azael*, this scar is deeply inset, being similar to that of *Phascolarctos* in shape and proximal extent.

**Medial condyle.** In anterior view, as in *P*. *azael*, the distal surfaces of the medial and lateral condyles sit approximately level. Viewed distally the medial condyle is more anteroposteriorly extensive than the lateral, but they are more alike here in *P*. *parvus* than in any of the other diprotodontoid femora studied. The medial condyle in this view also appears less bulbous and presents a flattened surface posteriorly, unlike the inflated and more laterally-canted condyle in *P*. *azael*.

**Lateral condyle.** The lateral condyle is short, broad and quadrangular in distal and posterior views, very closely resembling the morphology of *P*. *azael* in all respects.

**Patellar surface.** The patellar surface of the distal femur is anteroposteriorly and proximodistally short. The medial condyle of *P*. *parvus* projects anteriorly even less than in *P*. *azael*. Overall this creates a distal femur in stark contrast to those of other diprotodontoids and *Phascolonus*, again resembling extant wombat morphology most closely.

#### Tibia (Figs 30, 32 and 33)

**Fig 32:**
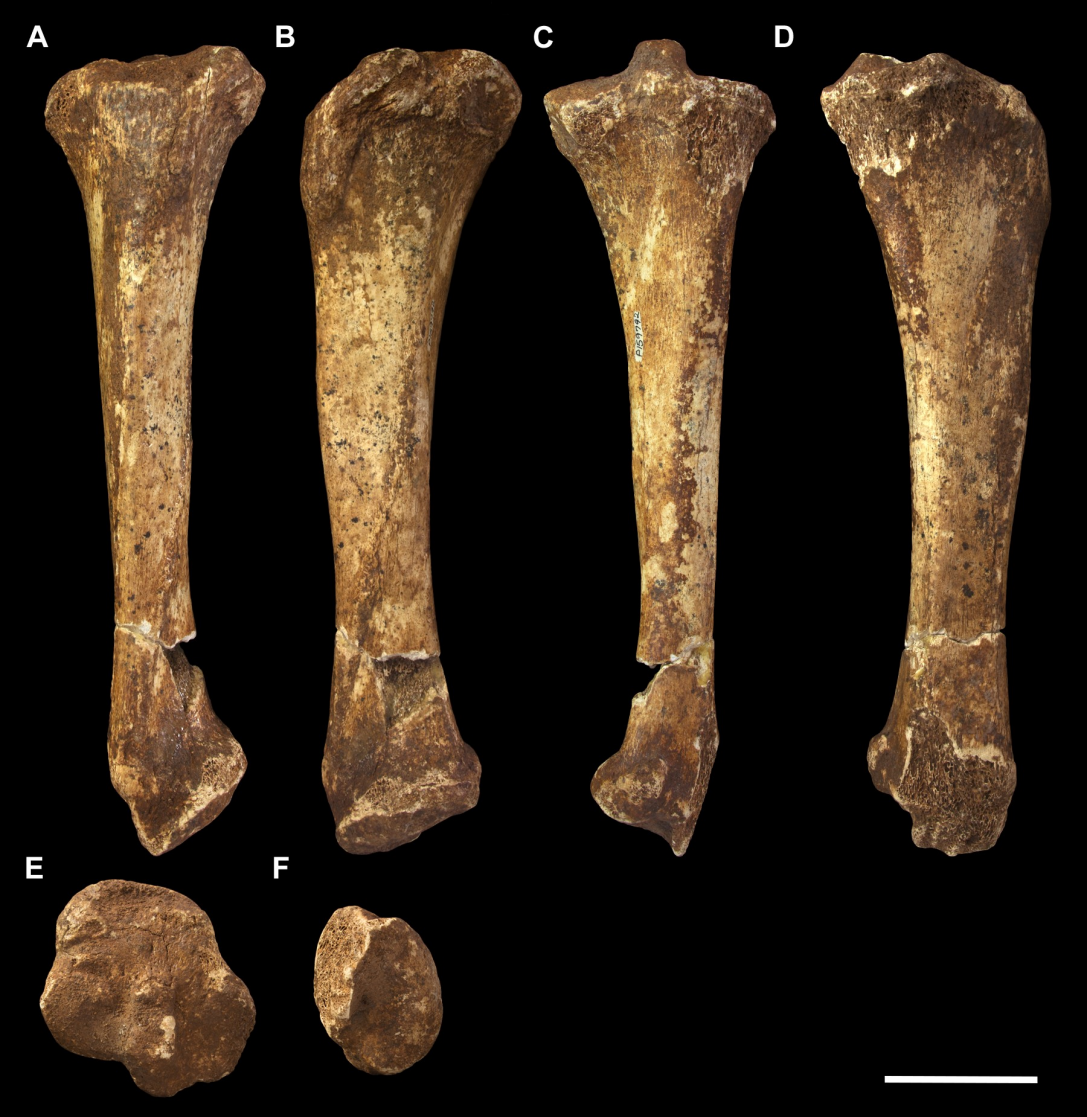
Right tibia of *Palorchestes parvus* NMV P159792. (A) Anterior; (B) lateral; (C) posterior; (D) medial; (E) proximal; (F) distal views. Scale bar 50 mm.

**Fig 33:**
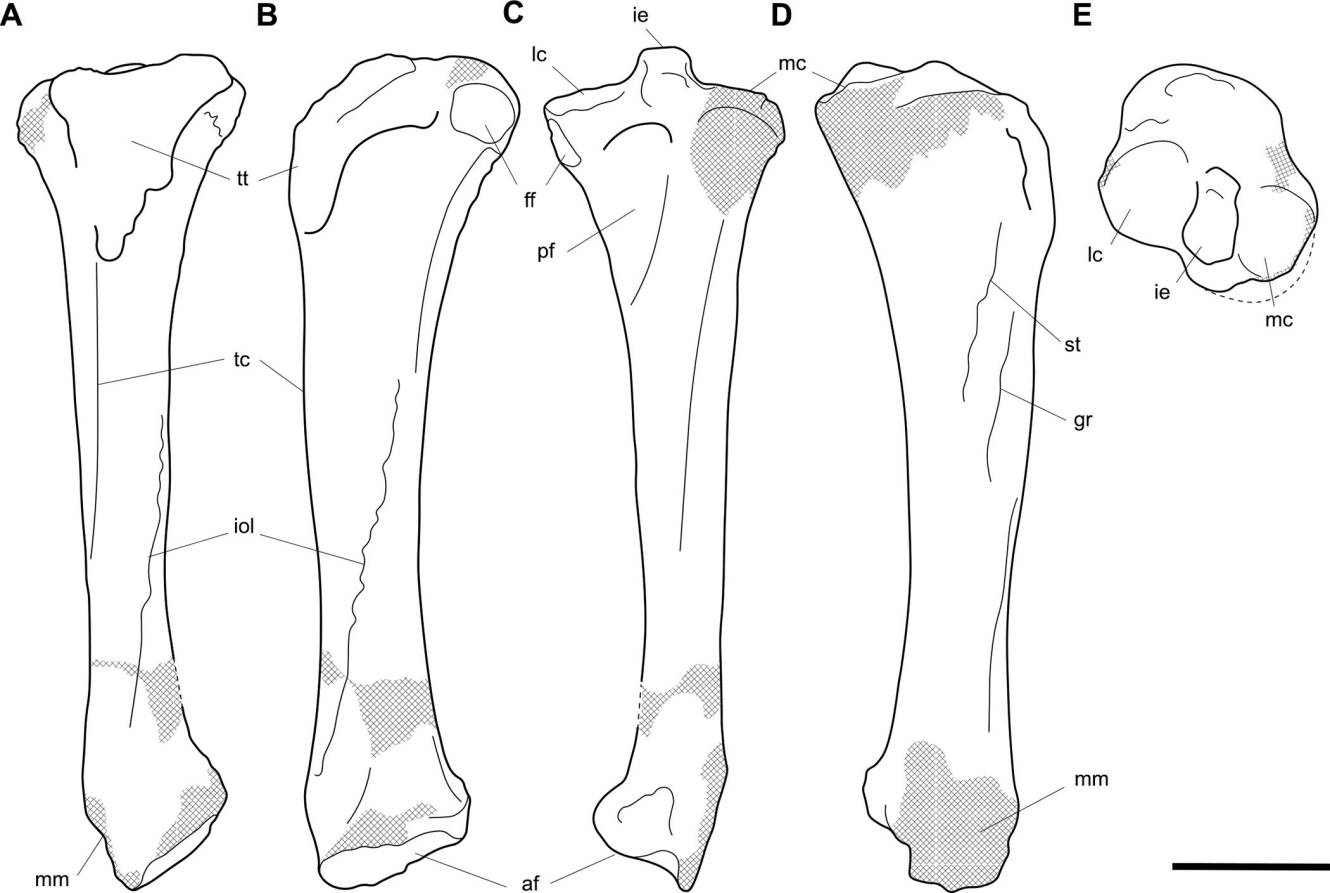
Labelled illustrations of the *Palorchestes parvus* right tibia NMV P159792. (A) Anterior; (B) lateral; (C) posterior; (D) medial; (E) proximal views. Hatching indicates surface damage to cortical bone, dashed lines indicate inferred bone contours. Abbreviations: **af**, astragalar facet; **ff**, fibular facet; **gr**, insertion of *m*. *gracilis;* **ie**, intercondylar eminence; **lc**, lateral condylar surface; **mc**, medial condylar surface; **mm**, medial malleolus; **pf**, popliteal fossa; **ptm**, posterior tubercle of medial malleolus; **st**, insertion of *m*. *semitendinosus;* **iol**, interosseus line; **tc**, tibial crest; **tt**, tibial tuberosity. Scale bar 50 mm.

The tibia of *P*. *parvus* is very similar morphologically to that of *P*. *azael*, the principal difference being that for approximately the same length it is more gracile, an allometric difference expected for the smaller species (Figs 30, 32 and 33). Differences to the *P*. *azael* morphology described above are provided below.

**Proximal articulations.** The medial condyle, though its posteromedial border is eroded, appears much smaller and less posteriorly extensive than in *P*. *azael*, probably representing a weightbearing allometric difference. The intercondylar eminence is substantially narrower mediolaterally in *P*. *parvus* than in *P*. *azael*. The fibular facet is preserved in both *P*. *parvus* specimens. It is distinct and expanded proximally to form a more laterally-facing circular shape than the flattened, inferiorly-directed oval facet of *Vombatus*, being more similar to that of *Phascolarctos*. This distinct facet does not resemble the more diffuse depression for the fibula in *Diprotodon* or *Zygomaturus* tibiae. This may indicate a more mobile and less weightbearing proximal tibiofibular articulation in *P*. *parvus*. The popliteal fossa on the posterior surface is more deeply furrowed than in *P*. *azael*, similar to that of vombatids.

**Diaphysis.** In *P*. *parvus* the diaphysis is more slender overall. In mediolateral view the tibial crest has a concave profile immediately below the tibial tuberosity before flaring out again at the point of insertion for *m*. *gracilis* roughly a third of the way down the diaphysis, strongly resembling the *Vombatus* condition. The interosseous border is more rounded than in vombatids and diprotodontids, but not quite to the extent seen in *P*. *azael*.

**Distal articulations.** The medial malleolus is eroded in the only specimen preserving the distal tibia, but from the available material it appears similar in proportion and extent to that of *P*. *azael*. The remaining astragalar surface on the distal tibia is much more steeply inclined and less laterally extensive than in *P*. *azael*, though similarly convex.

#### Pes (Figs 34 and 35)

**Fig 34:**
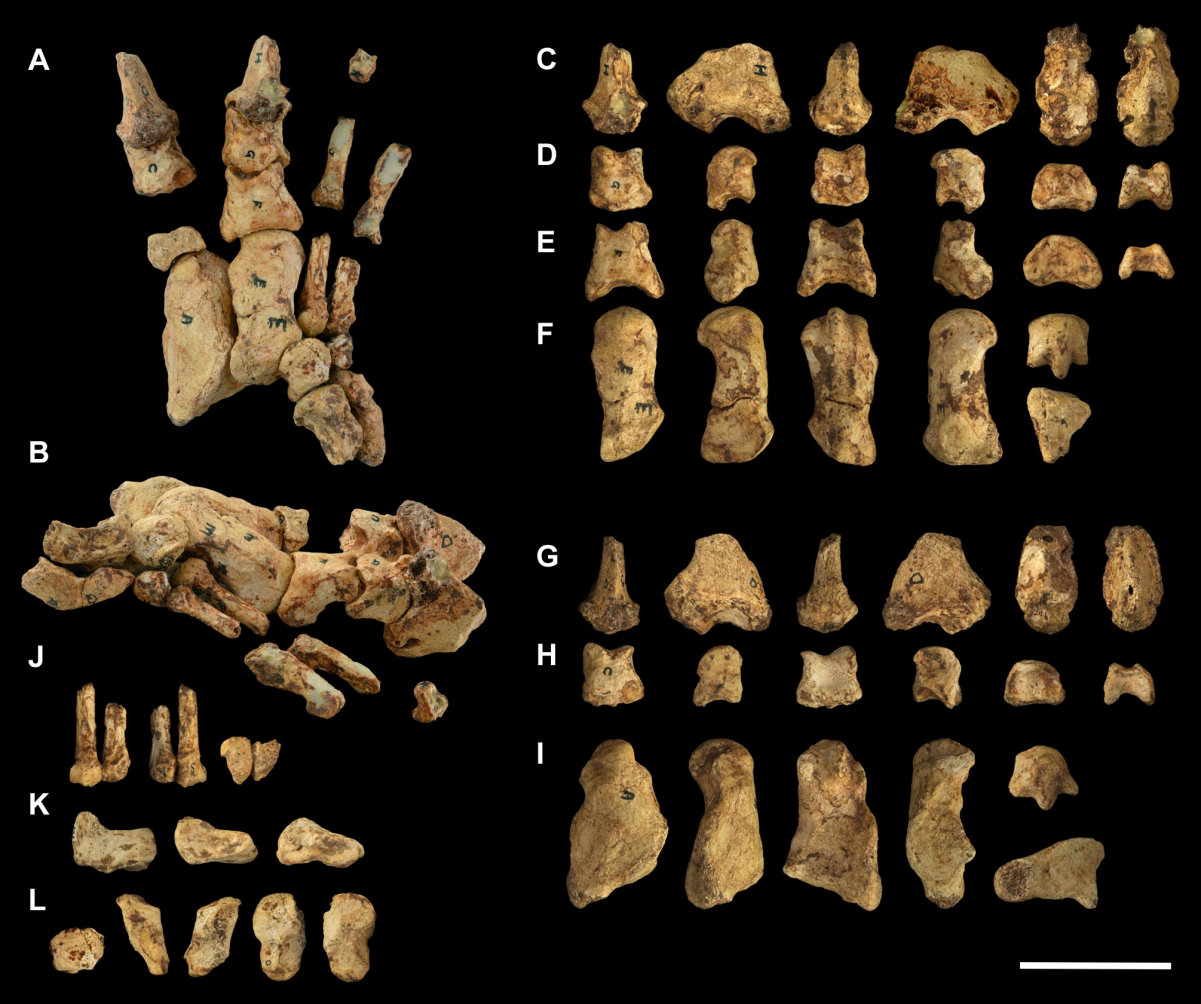
Associated left pes of *Palorchestes parvus* AM F58870. Articulated pes in (A) dorsal and (B) mediodorsal views; (C-E) digit IV ungual, intermediate and proximal phalanges in (left to right) dorsal, lateral, plantar, medial, proximal and distal views; (F) metatarsal 4 in (left to right, top to bottom) dorsal, lateral, plantar, medial, distal and proximal views; (G-H) digit 5 ungual and intermediate phalanges in dorsal, lateral, plantar, medial, proximal and distal views; (I) metatarsal 5 in (left to right, top to bottom) dorsal, lateral, plantar, medial, distal and proximal views; (J) metatarsals 2 and 3 (cemented together) in dorsal, plantar and proximal views; (K) navicular in dorsal, proximal and distal views; (L) ectocuneiform in dorsal, lateral, medial, proximal and distal views. Scale bar 50 mm.

**Fig 35:**
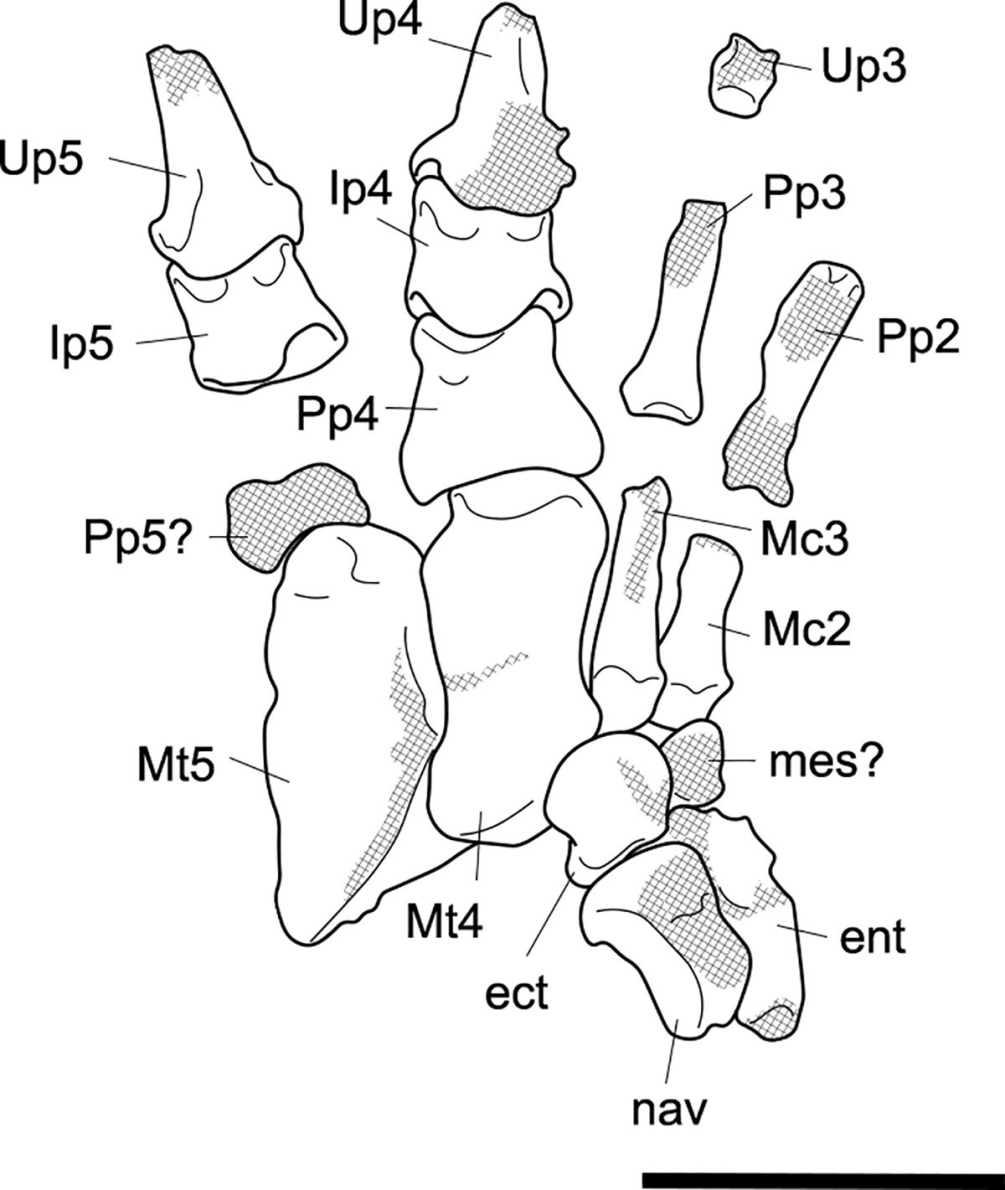
Labelled illustration of the articulated *Palorchestes parvus* left pes AM F58870 in dorsal view. Hatching indicates surface damage to cortical bone. Abbreviations: **ect**, ectocuneiform; **ent**, entocuneiform; **Ip4-5**, intermediate phalanges 4–5; **mes?**, possible mesocuneiform; **Mt2-5**, metatarsals 2–5; **nav**, navicular; **Pp2-4**, proximal phalanges 2–4; **Pp5?**, possible proximal phalanx 5; **Up3-5**, ungual phalanges 3–5. Scale bar 50 mm.

The digital posture of the pes in *P*. *parvus* appears clearly plantigrade, with the shape of the proximal interphalangeal joints indicating a habitually straightened position unlike the flexed posture in the manual equivalent (Figs 34 and 35). The second and third digits are extremely reduced relative to their robust lateral counterparts, more so than in *Nimbadon*, *Ng*. *tedfordi* or *Thylacoleo*.

**Ectocuneiform.** The ectocuneiform is intermediate in form between those of *Ng*. *tedfordi* and *P*. *azael*, being dorsoventrally longer and proximodistally thicker than the former, but with relatively smaller, dorsally positioned and more circular metatarsal facets than the latter. The facet for metatarsal 3 in particular is small, commensurate with the small proximal facet of this highly reduced syndactylous digit. Proximally, the facet for the navicular is flattened and dorsoventrally elongate as in *P*. *azael*.

**Entocuneiform.** The entocuneiform is highly eroded, with most facet edges and bony margins incomplete. Overall it is elongate, slightly inflected, and appears larger relative to the navicular than in other diprotodontoids. Distomedially the preserved surface of the facet for metatarsal 1 is quite flat, being less saddle-shaped than in *Nimbadon*.

**Navicular.** The navicular is similar in mediolateral length to that of *Ng*. *tedfordi*, but is anteroposteriorly much thicker and dorsoventrally compressed, with a less concave facet for the astragalus proximolaterally. Due to its increased thickness the *P*. *parvus* navicular has a much larger convex facet surface for the ectocuneiform and mesocuneiform distally, but a similarly elongate articular surface for the entocuneiform medially. The navicular is smaller relative to its neighbouring entocuneiform than in *Ng*. *tedfordi* or *Nimbadon* pedes.

**Metatarsals 2 and 3.** As is typical of diprotodontian marsupials, these elements are syndactylous and strongly reduced compared to the other highly robust pedal digits, with the third being slightly larger than the second. At their base they are triangular, with convex articular surfaces for the ecto- and mesocuneiforms. The third metatarsal has a dorsolateral facet at the base for articulation with metatarsal 4 as in *Diprotodon*. The diaphyses of both bones appear similar in dimensions. The distal ends are missing so lengths are unknown.

**Metatarsal 4.** The fourth metatarsal is shorter and relatively more robust than in *P*. *azael*. The concave, triangular cuboid facet is transected horizontally by a shallow sulcus. A facet for the ectocuneiform extends mediodistally from the medial edge of the cuboid facet and is smaller and more dorsally positioned than in *P*. *azael*. The mediodistal tip of this ectocuneiform facet projects from the medial shaft and on the ventral surface of this projection lies a small triangular facet for articulation with the dorsolateral facet of metatarsal 3. Viewed distally, the dorsalmost articular surface head is laterally canted, with slight reduction of the lateral keel as in the fifth metatarsal.

**Metatarsal 5.** The fifth metatarsal is slightly shorter than the fourth when measured between the base and head. But overall the fifth is longer owing to the very large lateral tuberosity that tapers proximally beyond the articular surface for the cuboid. This lateral tuberosity is similar in shape and proportion to that of *Phascolonus*, being a continual subtriangular flange originating at the distal end of the metatarsal. This is distinct from the more bulbous equivalent in diprotodontids and extant wombats. The lateral tuberosity lacks the dorsal uptick of the flattened *Ngapakaldia* equivalent. This tuberosity would have provided attachment for the *m*. *peroneus brevis* proximally, with another smaller tubercle distolaterally for the *m*. *abductor digiti V*. The smooth triangular cuboid facet on the base curves medially to provide articulation with the matching contour of the neighbouring fourth metatarsal. In ventral aspect the central keel of the head is laterally inclined with the lateral keel reduced, in contrast to the enlarged lateral keel described for *Nimbadon*, or subequal lateral keel in *Ngapakaldia*. Though we lack a proximal phalanx for this digit this morphology may indicate an abducted posture for the *P*. *parvus* fifth digit, and along with its lateral tuberosity shows high lateral loading of the pes.

**Phalanges.** The proximal interphalangeal joints of the pes are less restricted in extension due to slightly smaller dorsomedian crests. They were able to extend to a straightened position unlike the flexed manual equivalent. When viewed mediolaterally, their proximal articular fossae are less tightly concave and more open than the intermediate phalanges of the manus. In distal view, the condyles are more flared ventrally to create a wider sub-articular angle than the steep, more vertically-oriented distal condyles in the manus. In proximal view, the intermediate phalanges are slightly asymmetrical, with broader lateral facets.

**Unguals.** The non-syndactylous pedal digits 4 and 5 have unguals of very similar morphology to those of the manus. The dorsoventral extent of the condylar fossae and flattened plantar areas on the flexor tubercles indicate these pedal unguals were habitually held in maximal extension with their straight dorsal border parallel to the ground. Presumably this was to accommodate an even more ventrally recurved keratin claw sheath. The much smaller ungual for digit 3 is poorly preserved but has the same twin lunate condylar fossae which are slightly broader ventrally than dorsally and separated by a median keel, as those seen in other palorchestid unguals. Ventrally, this ungual lacks the plantar flexor tubercle seen in the manual and large pedal unguals, likely reflecting its reduced weightbearing role and weaker flexor tendon. The ungual process is broken off and not preserved.

Based on this associated specimen, the pedal unguals of digits 4 and 5 in *P*. *parvus* appear to have a dorsoventrally deeper flexor tubercle, with more deeply concave articular facets lying more dorsally on the bone than their manual equivalents.

***Propalorchestes* sp**. **Murray 1986**

#### Referred material

Measurements for all referred material below are provided in S1 Table.

All specimens were collected by T. H. Rich from Top Site, Bullock Creek, NT. As two *Propalorchestes* species are known from the Bullock Creek local fauna, and no postcrania are yet assigned to either, we refer the following elements to *Propalorchestes* sp. on the basis of their morphological distinctiveness from the other diprotodontoids which are well known from this locality, *Neohelos* spp. The articular surfaces of the unassociated humerus and ulna agree in shape but are from individuals of slightly different sizes.

**NTM P87115-6.** Left humerus (intact but for damage to the lateral margin of the lateral supracondylar crest). Photographed in Vickers-Rich et al. [72], pg. 170, Fig 232.

**NMV P253947.** Right ulna (proximal half, some damage to radial facet).

**NMV P179370.** Ungual phalanx.

#### Humerus (Figs 36 and 37)

**Fig 36:**
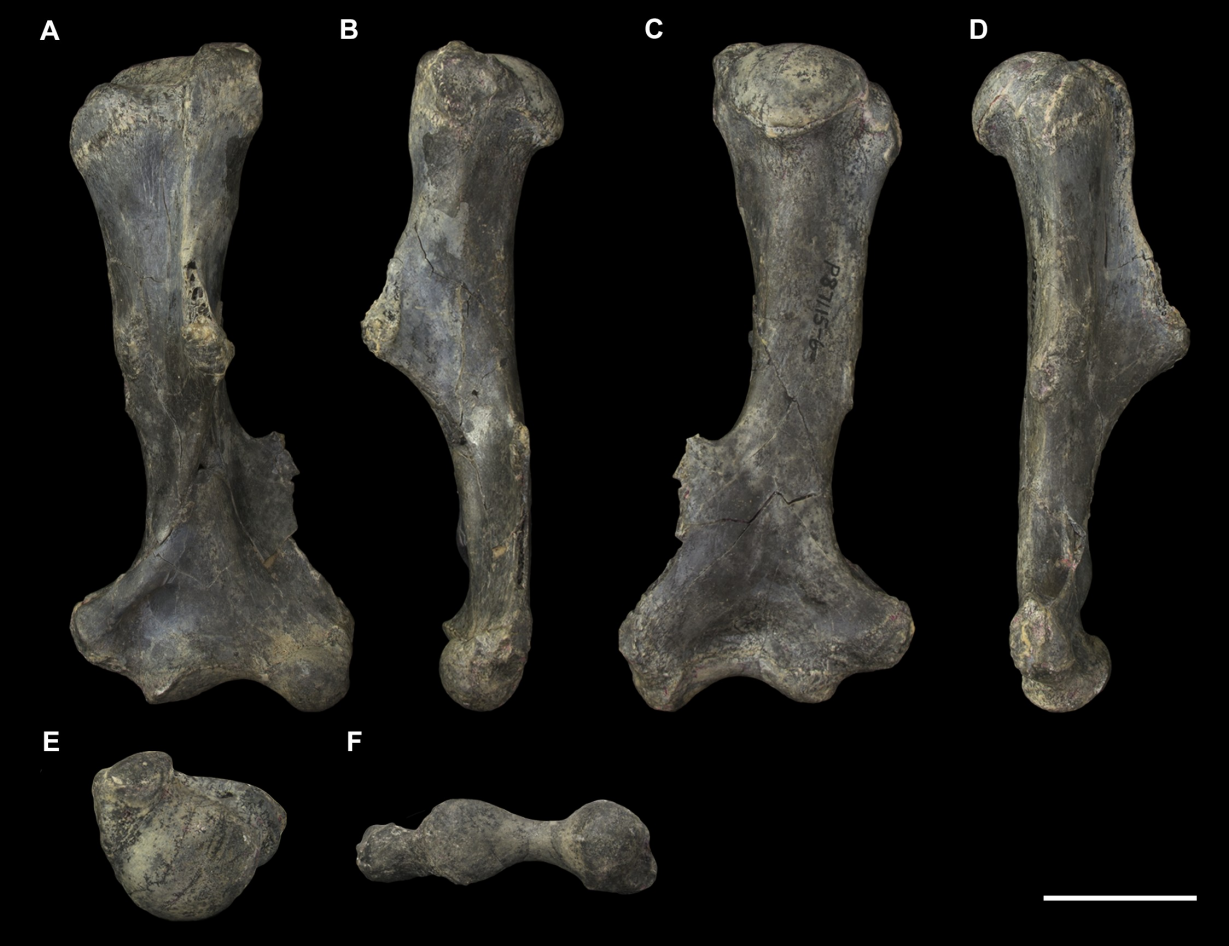
Left humerus of *Propalorchestes* sp. NTM P87115-6. (A) anterior; (B) lateral; (C) posterior; (D) medial; (E) proximal; (F) distal views. Scale bar 50 mm.

**Fig 37:**
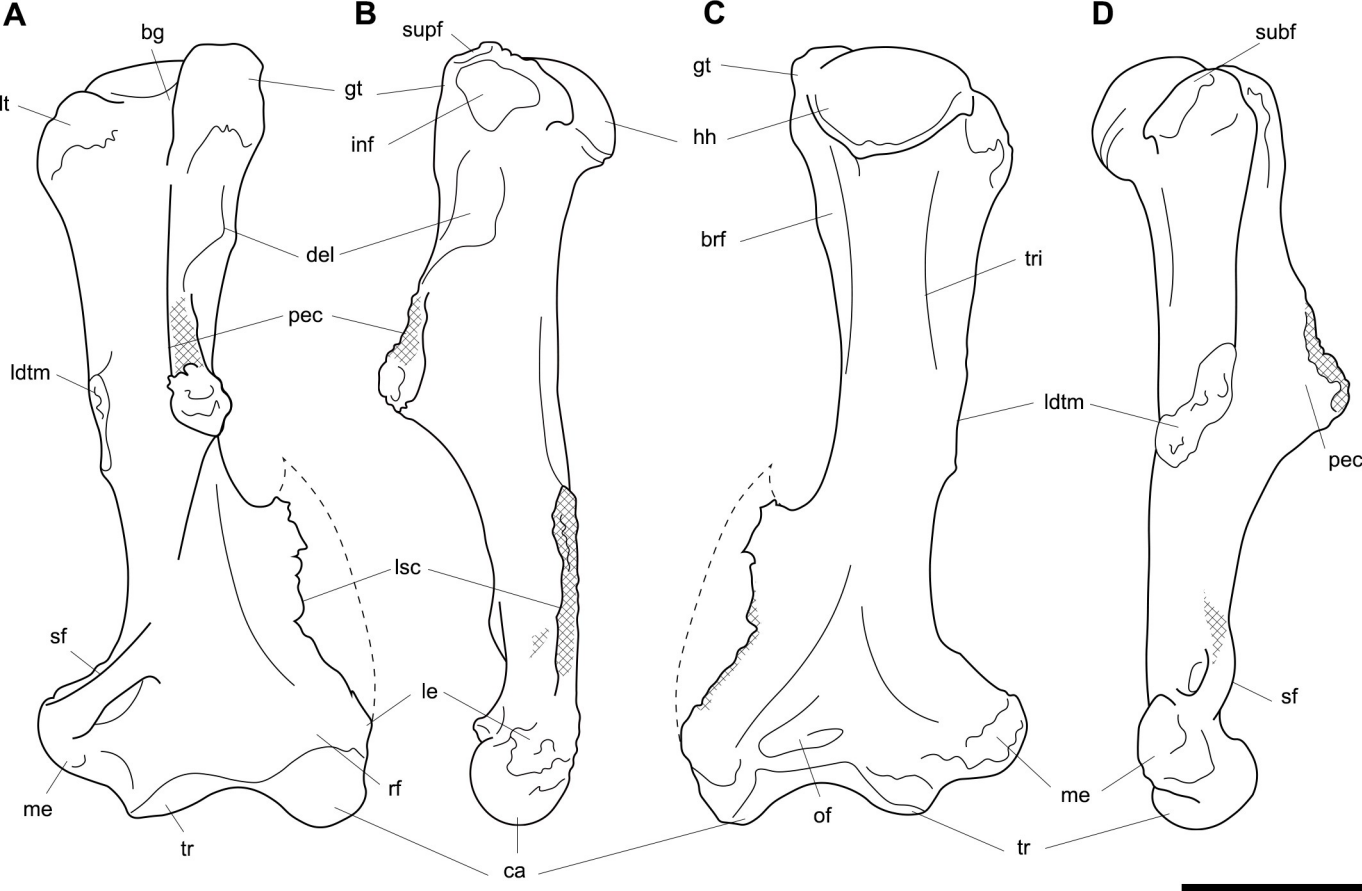
Labelled illustrations of the *Propalorchestes* sp. left humerus NTM P87115-6. (A) anterior; (B) lateral; (C) posterior; (D) medial views. Hatching indicates surface damage to cortical bone, dashed lines indicate inferred bone contours. Abbreviations: **bg**, bicipital groove; **brf**, fossa for *m*. *brachialis* origin; **ca**, capitulum; **del**, deltoid insertion; **gt**, greater tubercle; **hh**, humeral head; **inf**, fossa for insertion of *m*. *infraspinatus;* **ldtm**, insertion for *mm*. *latissimus dorsi* and *teres major;* **le**, lateral epicondyle; **lsc**, lateral supracondylar crest; **lt**, lesser tubercle; **me**, medial epicondyle; **of**, olecranon fossa; **pec**, pectoral crest; **rf**, radial fossa; **subf**, fossa for insertion of *m*. *subscapularis;* **supf**, fossa for insertion of *m*. *supraspinatus;* **sf**, supracondylar foramen; **tr**, trochlea; **tri**, origin for humeral heads of *m*. *triceps brachii*. Scale bar 50 mm.

The *Propalorchestes* humerus is a robust bone with a straight diaphysis and broad epiphyses, particularly the distal portion which is both laterally and medially expanded. The shaft has pronounced flanges anteriorly and laterally, for deltopectoral and wrist extensor muscles respectively (Figs 36 and 37).

Overall the humerus of *Propalorchestes* bears the strongest resemblance to that of *Ngapakaldia*, though it is larger than that of the latter (see S1 Table and S1 Fig).

**Head.** The humeral head is sub-hemispherical, with a posteriorly offset position creating the distinct ‘beak’ in medial view present in all vombatiforms. The head is slightly broader mediolaterally than anteroposteriorly, but not to the same degree seen in *P*. *azael* or the vombatid species and totally unlike the flattened, laterally-expanded head of *Neohelos*.

**Greater tubercle.** The greater tubercle in *Propalorchestes* projects proximally beyond the humeral head as in other palorchestids, unlike the in zygomaturine or diprotodontine species where it is subequal in height to the humeral head. The tubercle has two distinct attachment scars; a small fossa for *m*. *supraspinatus* presenting a flat surface superoanteriorly, and a larger fossa for *m*. *infraspinatus* with a directly lateral orientation, similar to that seen in other palorchestids. In lateral view the greater tubercle occupies two thirds of the anteroposterior extent of the proximal humerus, like other palorchestids. In proximal view the greater tubercle in *Propalorchestes* lies more anteriorly on the epiphysis than the laterally-positioned equivalent in *P*. *azael*. The tubercle overhangs medially to create a deep notch at the proximal part of the intertubercular sulcus, as seen in *P*. *parvus*. This contrasts with the shallow groove here seen in diprotodontines and vombatids.

**Lesser tubercle.** In *Propalorchestes* the lesser tubercle presents a flattened anterior surface, with margins slightly pointed proximally and rounded medially. This medial margin projects slightly more in *Propalorchestes* than in the other diprotodontoids, though not as much as in vombatids. In proximal view the tubercle is larger relative to the humeral head and more anteriorly situated than in other palorchestids, most closely resembling the extant wombat species in this respect. In medial view the well-defined insertion scan for *m*. *subscapularis* is obliquely oriented, descending posteriorly at an angle ~25° from vertical. This is similar to the orientation seen in *P*. *parvus*, steeper than in *P*. *azael* (~40°) and vombatids (~30°) and much steeper than in diprotodontids (> 55°).

**Pectoral crest.** Descending from the broad greater tubercle, the pectoral attachment in *Propalorchestes* narrows to a tall crest which runs sub-vertically down the approximate midline of the anterior humeral shaft. In medial view the crest is a broad-based isosceles triangle, reaching its apex slightly proximal to the midpoint of the humeral shaft. This makes it the shortest pectoral crest among the vombatiforms relative to total humeral length. Viewed anteriorly, its medial edge curls slightly over the bicipital sulcus, but to a far lesser extent than in other palorchestids. The apex of the crest terminates in a bulbous protuberance resembling that of *P*. *parvus*. However, the deltoid insertion does not converge directly on this protuberance as in the latter species. Instead, the insertion for the deltoid muscle is a poorly-defined curved scar. This scar is bent anteromedially from a site beneath the greater tubercle to merge with the pectoral crest approximately halfway down its length (a quarter of the way down the shaft). This is distinct from the morphology in *P*. *parvus* and vombatids where the deltoid insertion ridge is discrete and runs parallel lateral to the pectoral crest before converging on it distally. It also differs from small diprotodontids like *Neohelos* in which the deltoid insertion scar is a weakly defined vertical facet that merges with the distal apex of the pectoral crest.

**Tuberosity for *mm*. *teres major* and *latissimus dorsi*.** In *Propalorchestes* the attachment scar for the *mm*. *latissimus dorsi* and *teres major* is a sharply defined ovoid on the lateral shaft just distal to its midpoint. Like other palorchestids, its posterior margin is recurved and overhangs the posterior surface of the diaphysis. Two distinct scars of subequal size are discernible within this ovoid in the *Propalorchestes* humerus; an anteroproximal, slightly bulging scar, and a posterodistal rugose portion.

**Diaphysis.** The shaft of the *Propalorchestes* humerus appears straight in anterior and medial views. The posterior surface of the shaft is not strongly scarred, though fossae for the humeral heads of *m*. *triceps brachii* and *m*. *brachialis* are discernible, and a deeply excavated olecranon fossa is present distally. This olecranon fossa is much deeper than in *P*. *parvus* and instead resembles the ulnae of *Ngapakaldia* species in form and positioning. As in *P*. *parvus*, the *Propalorchestes* ulna lacks the strong origin scar for *m*. *epitrochleoanconeus* seen in *P*. *azael*. In overall section the shaft is a laterally-expanded oval, distorted anteriorly by the large pectoral crest and distally by the sheet-like lateral epicondylar crest arising on its distolateral margin. Unlike in *Palorchestes* species, a deep radial fossa lies proximal to the capitulum on the anterodistal shaft surface.

**Lateral epicondyle.** The lateral epicondyle only barely projects from the lateral edge of the capitulum in *Propalorchestes*, making it the least developed of the palorchestids in this respect. In lateral view it tapers proximally into the supinator crest almost immediately. Its surface is strongly rugose, with sharply defined scarring present on all sides and a distinct fossa for the attachment of the lateral collateral ligament. In distal view, the lateral epicondyle is slightly posteriorly deflected from the transverse axis of the distal epiphysis, unlike the aligned, undeflected condition in *Palorchestes*.

**Trochlea.** In *Propalorchestes* the humeral trochlea is a domed shape presenting slightly more articular surface on the anterior side than posteriorly. It is more convex than in *P*. *parvus* and much more so than the flattened trochlea of *P*. *azael*. The trochlea and capitulum, though subequal in size, are distinct and separate from one another. A ~115° angle separates the two in anterior view, and there is a narrow constriction dividing their dilated surfaces when viewed inferiorly. This is similar for most vombatiform humeri except for *Neohelos* in which this constriction is much thicker and a much shallower angle (> 150°) separates the structures in anterior view. Viewed inferiorly the *Propalorchestes* trochlea is ovoid, with its long axis slightly anteriorly deflected to lie offset from the transverse axis of the medial epicondyle as in other palorchestids. This is distinct from vombatid and *Diprotodon* humeri, where the medial epicondyle is posteriorly deflected from the trochlear axis, and from *Ngapakaldia*, where the trochlea is aligned with the medial epicondyle. On the posterior aspect the trochlear surface is barely visible, while a deeply excavated triangular olecranon fossa.

**Capitulum.** The *Propalorchestes* capitulum is large and hemispherical in anterior view, unusual amongst vombatiforms in its marked distal offset from the transverse plane of the distal articular surface, giving the humerus a slightly lopsided appearance. In lateral view the anteroposterior extent of the capitulum is shallower (as in all palorchestids) than the proportionally deep capitula of other vombatiforms. Viewed distally, the *Propalorchestes* capitulum tapers slightly to form a small capitular tail, but not to the extent of the pronounced tail seen in *Ngapakaldia* where it forms a lateral buttress for the olecranon process when the elbow is fully extended. Such buttressing is weak in the *Propalorchestes* humerus, which in posterior view shows only a small amount of capitular surface compared with other palorchestids and vombatiforms generally.

**Supracondylar foramen.** The supracondylar foramen is a well-defined ovoid canal. In orientation it more closely resembles the arrangement in *P*. *parvus* than the near-vertical foramen in *P*. *azael*.

**Medial epicondyle.** The medial epicondyle in *Propalorchestes* is a rounded, medially-projecting rugose process. It is greatly expanded to create a broad distal epiphysis. In distal view the medial epicondyle accounts for 29% of the total distal epiphyseal width, comparing closely to *P*. *parvus* (29%), *Ngapakaldia* (25%) and extant wombat (27%) humeri, but not quite approaching the proportions in *P*. *azael* (32%). The medial epicondyle lies in the same dorsal (coronal) plane as the lateral supracondylar crest.

#### Ulna (Figs 38 and 39)

**Fig 38:**
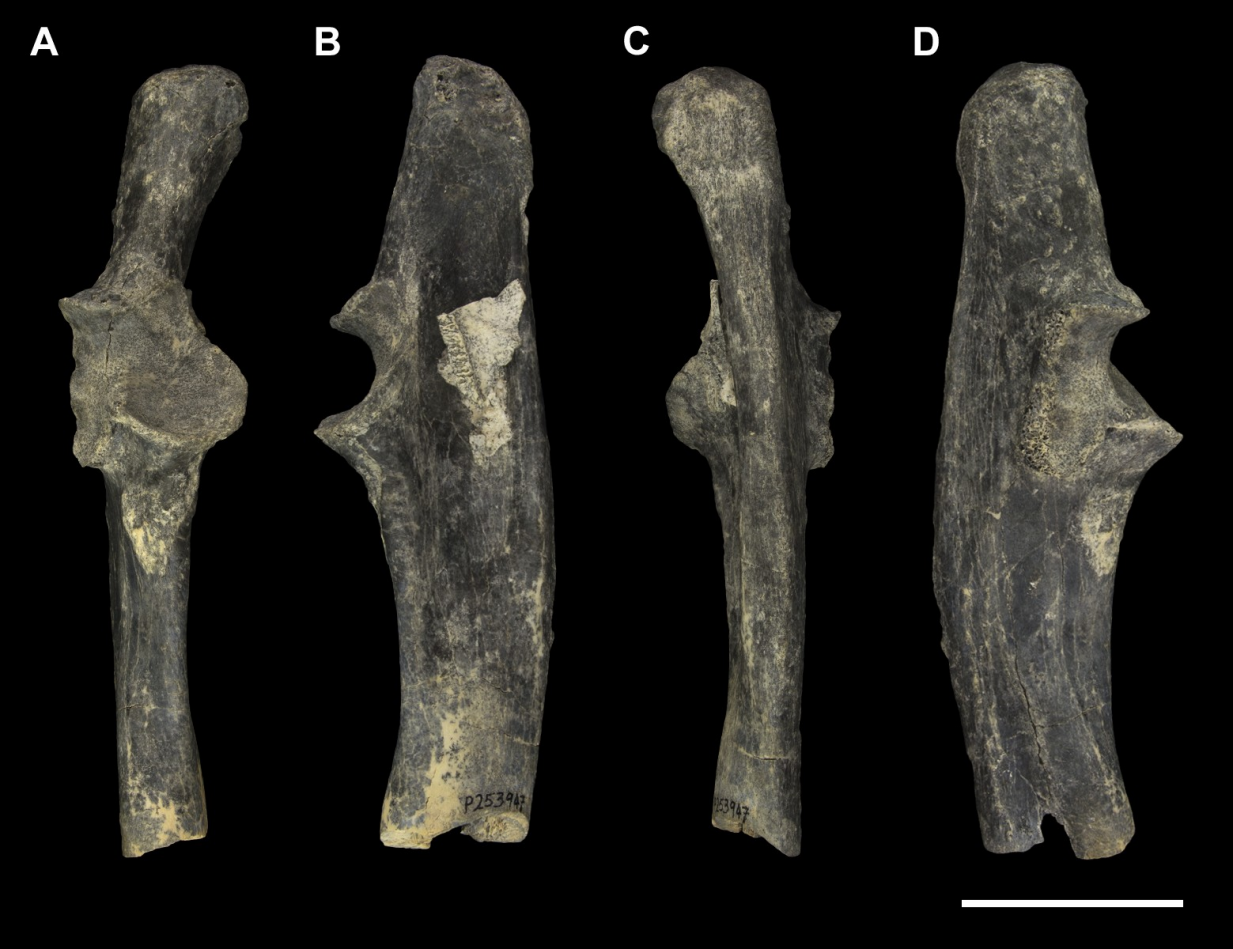
Right ulna fragment of *Propalorchestes* sp. NMV P253947. (A) anterior; (B) medial; (C) posterior; (D) lateral views. Scale bar 50 mm.

**Fig 39:**
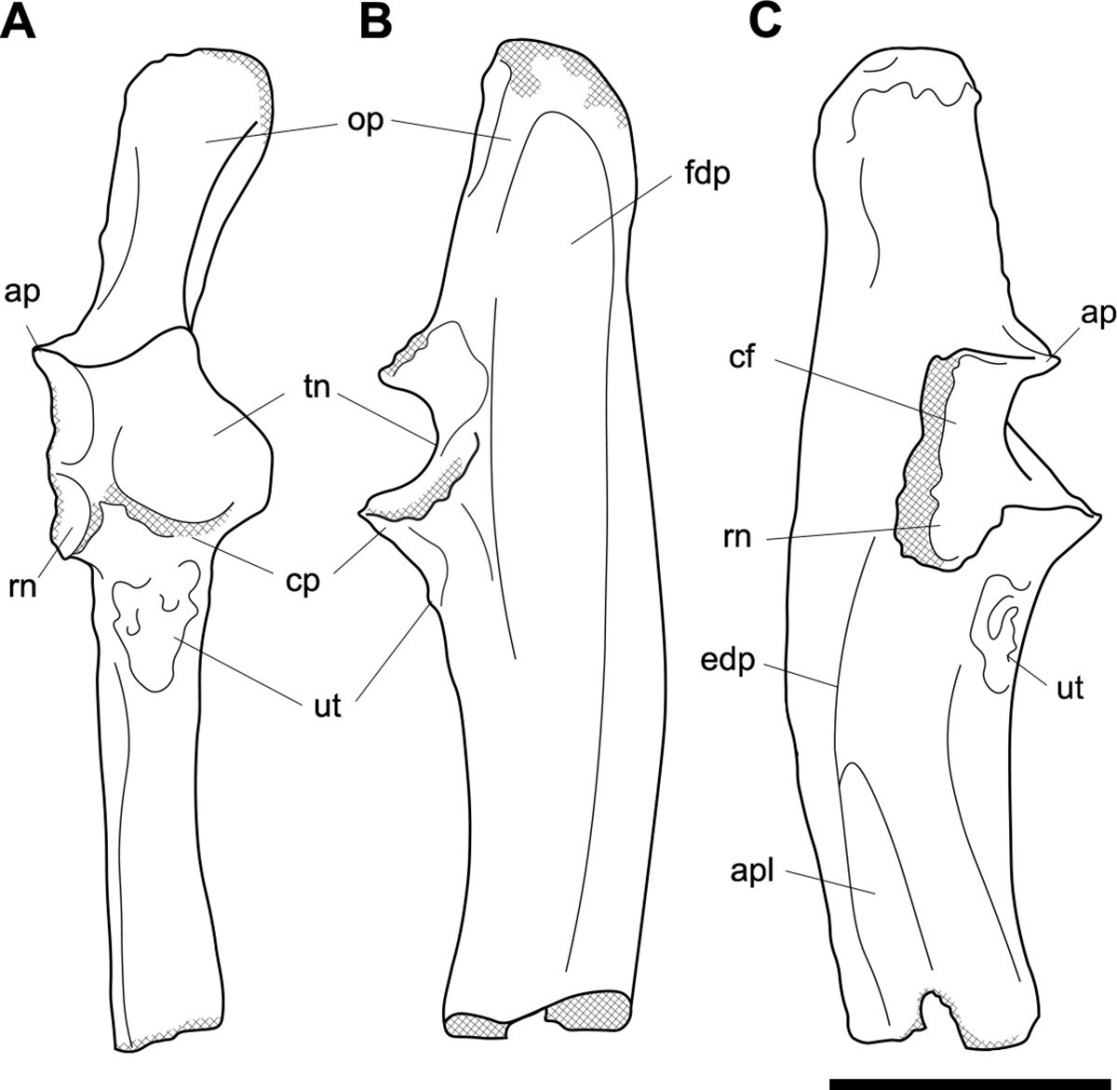
Labelled illustrations of the *Propalorchestes* sp. right ulna fragment NMV P253947. (A) anterior; (B) medial; (C) lateral views. Hatching indicates surface damage to cortical bone. Abbreviations: **ap**, anconeal process; **apl**, origin for *m*. *abductor pollicis longus;* **cf**, capitular facet; cp, coronoid process; **edp**, origin for *m*. *extensor digitorum profundus;* **fdp**, fossa for origin of *m*. *flexor digitorum profundus;* **op**, olecranon process; **rn**, radial notch; **tn**, trochlear notch; **ut**, ulnar tuberosity. Scale bar 50 mm.

The *Propalorchestes* ulna strongly resembles that of *Vombatus* in proportions and morphology but is approximately 50% larger, similar in overall size to those of *Thylacoleo* and *Ng*. *bonythoni*, albeit with a much longer olecranon relative to the humeral articular surface than either of the latter (Figs 38 and 39). The ulna NMV P253947 is missing its distal half, so the total length and distal epiphyseal morphology are not known (though we approximated the length to be 276 mm based on *Vombatus* proportions).

**Olecranon.** The olecranon process is elongate, medially deflected and proximally enlarged, nearly identical to *Vombatus* in all respects bar absolute size. It is less mediolaterally thick and proximally bulbous than in *Ng*. *bonythoni*. In medial view, the ventral border of the olecranon tapers slightly towards the bulbous proximal tip, again similar to that of *Vombatus* and quite unlike the more regular, quadrangular olecranon in *P*. *azael*.

**Trochlear surface.** The *Propalorchestes* ulna has a scooped, subcircular trochlear surface. It resembles *Vombatus*, *Ng*. *bonythoni* and *P*. *parvus* trochleae more than the elongate, narrow and flat surface in *P*. *azael*, but is relatively broader than in any of the former taxa.

**Coronoid process.** The coronoid process appears relatively shorter dorsoventrally than in *Vombatus;* however this is due to the relative increase in dorsoventral depth of the *Propalorchestes* shaft immediately distal to it, likely for increased load bearing.

**Anconeal process.** The anconeal process is tall and in lateral view rises roughly perpendicular to the longitudinal axis of the ulnar shaft, though not as tall or proximally curled as in wombats. This curled anconeal process in wombats articulates with their pronounced capitular tail on the humerus, a feature lacking in palorchestid humeri. The anconeal process is less laterally deflected in the transverse plane than in *P*. *azael* or *Ng*. *bonythoni*, instead resembling the *P*. *parvus* condition.

**Radial notch.** The radial notch sits in a ventral position on the lateral shaft, resembling *P*. *parvus*, *Ng*. *bonythoni* and *Vombatus* ulnae in this regard rather than the dorsal positioning of the notch in *P*. *azael*. Its perimeter is eroded in NMV P253947, but the preserved morphology indicates a wide concave platform for the head of the radius, unlike the proximo-distally narrow, curved notch in extant wombats.

**Ulnar tuberosity.** In *Propalorchestes*, the ulnar tuberosity takes the form of a raised subtriangular area of rugosity as in wombats and *Ng*. *bonythoni*, rather than a distinct tubercle as seen in *Palorchestes* species. However, it does resemble the morphology of *Palorchestes* in its position on the ventrolateral border of the shaft, well distal of the coronoid process and radial notch unlike the condition in wombats.

**Diaphysis.** In lateral view, the ulnar shaft has a posteriorly convex dorsal border. Based on the uptick in the ventral contour immediately proximal to the break, the bone may have had the gently sinuating shaft contour of extant wombats rather than the gradual ventral concavity seen in *P*. *azael*. The attachment scar for *m*. *abductor pollicis longus* is strongly expressed and lies both more dorsally on the lateral shaft and arises more distally than in *Vombatus* (but not as distal as in *P*. *azael*). The attachment scar for *m*. *extensor digitorum profundus* is less rugose than in *Vombatus* and is more dorsal on the lateral shaft, as in *P*. *azael*.

#### Ungual (Fig 40)

**Fig 40:**
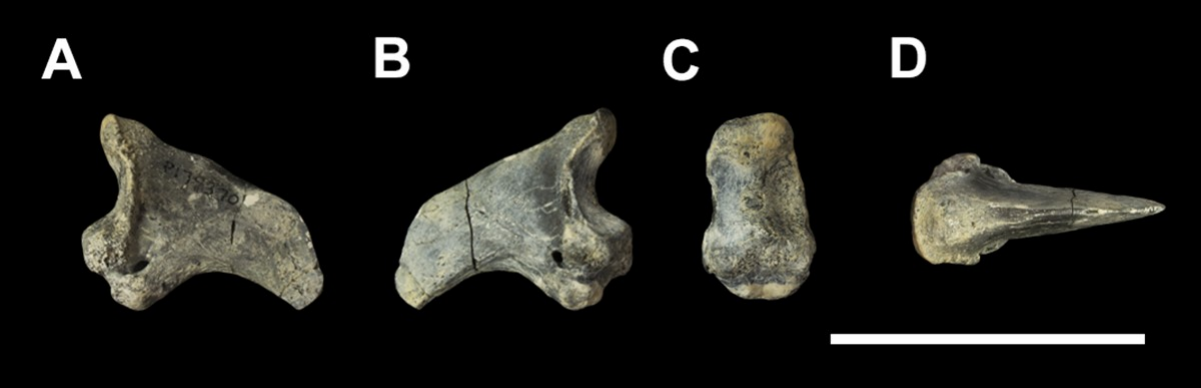
Ungual phalanx of *Propalorchestes* sp. NMV P179370. (A) Lateral; (B) medial; (C) proximal; (D) dorsal views. Scale bar 50 mm.

The single known *Propalorchestes* ungual (NMV P179370, Fig 40) is similar to the contemporaneous *Nimbadon* but is absolutely larger than all examples we observed of that taxon. This size difference is most marked in proximal view, with the *Propalorchestes* ungual also possessing a much more dorsally deflected extensor process. Compared to its more derived palorchestid kin, the ungual is slightly shallower dorsoventrally, with a proportionally thicker ungual process, but the unique palorchestid shape is evident even in this early species. The flexor tubercle is more discrete from the rest of the proximal ungual than in *Palorchestes* species, being in proximal view slightly narrower than the articular facets dorsal to it, and in lateral view slightly smaller relative to the rest of the ungual.

### Palorchestid body mass estimate results

Body mass estimates for palorchestid specimens are presented in Table 3, with values given in kilograms representing the prediction error around each point estimate. As per the findings of Campione and Evans [63], the equation derived from combined humeral and femoral circumference data had the most predictive power, with the lowest PPE and SEE of the three models, followed by humerus-only, with the femoral-only equation giving the lowest predictive power.

**Table 3:** Palorchestid body mass estimates.

Taxon	Humerus specimen #	C_H_ (mm)	Femur specimen #	C_F_ (mm)	C_H+F_ (mm)	C_H_ mass estimate (kg)	C_F_ mass estimate (kg)	C_H+F_ mass estimate (kg)
*Palorchestes azael*	SAMA P55199	179	-	-	-	**788** ± 125	-	-
	NMV P159792	191	NMV P26534	161	352	**926** ± 265	**627** ± 212	**791** ± 202
	NMV P157144	235	NMV P157144	201	436	**1597** ± 458	**1160** ± 392	**1413** ± 362
	NHMUK PV OR 46914	258	-	-	-	**2059** ± 629	-	-
*Palorchestes parvus*	AM F58870	145	AM F58870	122	267	**438** ± 125	**289** ± 98	**367** ± 94
*Propalorchestes* sp.	NTM P87115-6	98	-	-	-	**155** ± 45	-	-

Model I OLS regression formulae

Humeral circumference: logBM = 2.662*logC_H_− 0.108, R^2^ = 0.986, PPE = 27.5%, SEE = 0.145

Femoral circumference: logBM = 2.809*logC_F_− 0.401, R^2^ = 0.979, PPE = 34%, SEE = 0.175

Combined humeral and femoral circumferences: logBM = 2.752*logCH+F− 1.110, R^2^ = 0.988, PPE = 25.5%, SEE = 0.133

Estimates are given for all individuals for which stylopodia are known, ± prediction error for each estimate in kilograms. Mass estimates for other comparative vombatiform taxa calculated using this method are provided in S2 Table. Abbreviations: C_H_, minimum humeral circumference; C_F_, minimum femoral circumference; C_H+F_, total minimum humeral and femoral circumference; PPE, percent prediction error; SEE; standard error of the estimate.

As expected, where associated stylopodia were available, estimates incorporating humeral circumferences were heavier than those using the femur, reflecting the irregular minimum humeral section profile typical of these animals. The maximal body mass predictions for each species, calculated using humeral circumference only, were 155 kg for *Propalorchestes*, 438 kg for *Palorchestes parvus* and 2059 kg for *P*. *azael*. Femur-based estimates were much smaller, with 289 kg for *P*. *parvus* and 1160 kg for the largest example of *P*. *azael*. This large individual yielded estimates of 1597 kg from the humerus-only and 1413 kg from the combined formulae, demonstrating how widely the estimates differ between humeral and femoral models.

Mass estimates for comparative vombatiform taxa are available in S2 Table. Prior predictions for *Diprotodon* and *Nimbadon* (see Table 1) fell within the bounds of prediction error of our estimates generated using the combined circumference model. Our estimates for *Neohelos* and *Thylacoleo* were substantially lower than existing predictions (likely due to our particular specimens), while other results were considerably greater–our *Phascolonus* estimates ranged from 460–737 kg, while our estimate for a *Zygomaturus* specimen surpassed one tonne.

## Discussion

### Palorchestids were larger than previously suspected

The mass values presented in Table 3 are the first empirical estimates for body mass in Palorchestidae. In all cases we found masses to be substantially heavier than existing predictions: the early palorchestid *Propalorchestes* may have weighed around 150 kg, *Palorchestes parvus* approximately 300–400 kg, and *P*. *azael* possibly more than one tonne.

Campione and Evans [63] found that combined circumference data from both stylopodia provided the most robust estimates across tetrapods generally. However, they found taxa with highly apomorphic humeral morphology (talpids) were extreme outliers, even in their combined circumference model, and opted to exclude them. We posit that the highly derived morphology of the *P*. *azael* humerus produces similar overestimates which should be interpreted with caution (this may also apply to the unusual humerus of *Phascolonus*, see S2 Table). We suggest that femoral circumference alone is a more conservative metric by which to estimate body mass for *P*. *azael*, despite the lower precision of the femur-based equation overall. To estimate mass via the femur alone is to assume that *P*. *azael* distributed its weight across the fore- and hindlimbs in the same way as the extant taxa in the dataset from which our equations were derived (S2 Table). Until more is known about the postcranial body form and habitual locomotion of this species, such assumptions must be made as we cannot yet predict the placement of the centre of mass. Where a femur was available, *P*. *azael* body mass estimates were lower than for the associated humerus. No associated femur is known for the largest humerus NHMUK PV OR 46914, which itself yielded an estimate of 2059 kg, an implausible value approaching the mass of the objectively larger *Diprotodon*. However, based on humeral dimensions that individual would certainly have been even bigger than NMV P157144 (S1 Table). So, while our body mass estimates will necessarily have wide error margins, it does appear that *Palorchestes* species were heavier members of the Australian Pleistocene fauna than previously suspected. This has deep implications for future inferences of many aspects of their palaeobiology including life history strategy, range size and feeding ecology.

Our body mass estimates for *P*. *azael* overlapped with the size range we found for *Zygomaturus* specimens (see S2 Table). Gigantism evolved independently in both the diprotodontid and palorchestid lineages after their divergence in the Palaeogene, as part of a broader trend toward larger body mass among the Australian Pleistocene megafauna [7, 38]. Though *P*. *azael* is invariably rare, both species co-occur in assemblages across the continent [30, 73, 74], so the likelihood that *P*. *azael* converged on a similar body size to *Zygomaturus* provides additional support for their special adaptation to a unique niche separate from this related taxon.

In light of the possibility that *P*. *azael* and *P*. *parvus* may have been sympatric at the Buchan site (Table 2), the body size disparity we have shown between the species is interesting. Trusler and Sharp [37] note that the coexistence of palorchestid species at various periods in their evolutionary past–particularly during the Pleistocene–shows that smaller species were able to persist in the same environmental conditions in which their close relatives achieved gigantism. While *P*. *azael* may have been able to use its extraordinary forelimbs to exploit higher volumes of poorer-quality browse, smaller species like *P*. *parvus* may have continued targeting the food sources they were ancestrally adapted for. This aligns with dental evidence showing that *P*. *azael* appears to have had morphological (and perhaps dietary) affinities divergent from the smaller *P*. *parvus* (and *P*. *pickeringi*), with which it may have been sympatric at several localities [46].

With multiple humeri representing several individuals we were able to provide some idea of the notable body size variation within *P*. *azael*. The two smaller *P*. *azael* humeri have open metaphyses which suggests these animals died before reaching an asymptotic size. However, the two larger specimens both appear to have fused metaphyses while differing in length by approximately 30%. The size range represented here supports variability in dental dimensions observed by Trusler [36] in *P*. *azael* individuals of similar dental wear and eruption stages. Both indeterminate growth and sexual size dimorphism are common throughout Order Diprotodontia [75, 76] and are suspected to have occurred in some diprotodontids [21, 42]. But, like Trusler [36], we are similarly hindered by small sample sizes and are unable to comment on ontogenetic variation or the likelihood of sexual dimorphism in these species. Likewise, without more precise dating information it is impossible to know how close these humeri are temporally–in principle, they could represent adult individuals two million years apart in evolutionary time.

### Specialisation of the palorchestid appendicular skeleton occurred much later than the craniodental anatomy

The preceding descriptions depict a morphocline within the palorchestid lineage toward the highly derived forelimb seen in the largest and latest species *Palorchestes azael*. Prior analyses of the cranial morphology of the group have recognised that their apomorphic cranial characteristics were already well-established in the smaller, earliest-known species and that the origin of their specialised rostrum was not associated with increasing body size [28, 36, 37, 68]. Here we demonstrate that specialisation of the postcrania was delayed relative to the skull, and may indeed have been linked to increasing body size. That is, the forelimb morphology of the mid-Miocene *Propalorchestes* is similar to other small early Miocene diprotodontoids such as *Ngapakaldia*, and the truly unusual forelimb did not arise until the Late Pleistocene giant *P*. *azael*. From this ancestral morphology, palorchestid forelimbs responded very differently to increasing body size than their sister diprotodontids, with shortening and widening of the humerus relative to the ulna, elongation and straightening of the olecranon process, and eventual ‘locking’ of the elbow in a flexed position (see discussion below). The inverse appears to have occurred in giant diprotodontids–they are characterised by lengthened humeri, short ulnae with posteriorly deflected olecranon processes and more columnar limb posture with increased trochleation of the elbow (see S1–S3 Figs). Divergence in palorchestid hindlimb morphology from diprotodontids is subtler, but is especially evident in the femur and the pes. In many respects even the more derived palorchestids actually resemble vombatids in their limb anatomy, however with so few fossil vombatid species represented by postcrania it is difficult to say how close this resemblance truly is.

### Forelimb adaptations

*Propalorchestes*, with its broad distal humerus, large muscle insertion processes and a long, medially-curved olecranon process, already had the hallmarks of a fairly powerful and specialised forelimb user (Figs 36–39). *Palorchestes parvus* was an animal with even more muscular forelimbs and slightly reduced ROM in elbow flexion than its predecessor (Figs 19–22). But undoubtedly, the most exaggerated palorchestid forelimb anatomy is found in *Palorchestes azael*, with its flat, seemingly-immobile humeroulnar joint setting it apart from all its vombatiform kin (Figs 3–6).

#### The elbow was fixed in *Palorchestes azael*

The shape of the humeroulnar articulation in *P*. *azael* would have effectively fixed the elbow in a flexed posture approaching a 100° angle, a condition seen in no other marsupial or placental mammal known to the authors (Figs 3–6). This elbow appears to be a palorchestid adaptation for a particularly specialised use of the forelimb, likely in acquiring food. This dietary niche was apparently occupied by the earliest known palorchestids as evidenced by their already derived craniodental and claw morphology, both of which persisted in the lineage as body size increased. The immobilisation of the elbow may represent a compromise that arose in later species which attained giant body size. Fixation may have been necessary to stabilise the joint in a particular posture (see below) and resist rotational forces at the elbow during feeding activities in a much heavier animal. Such stabilisation would be reinforced by the relatively sharp angle between the trochlea and capitulum, additionally bracing the elbow against lateral stresses.

When considering the uniqueness of the humeroulnar joint in *P*. *azael*, it must be noted that their humeroradial joint displays curiously unspecialised morphology. It does not seem to vary sympathetically with the humerus and ulna to ‘compensate’ for the reduced mobility of the elbow by providing increased pronation and supination. The radius is not particularly bowed, nor does the articular circumference of the radial head appear to allow any greater rotational movement than in extant wombats or *Ngapakaldia*, and the pronator muscle attachment scars along its diaphysis are in all cases less rugous and pronounced than their relatives (Figs 7 and 8D). It appears the humeroradial joint of this animal did not have especially large rotational ROM, limiting these movements again in favour of stability. This indicates that the freedom and positioning of the manus was limited and is further evidence for the highly specialised nature of the *P*. *azael* forelimb.

Some other (notably xenarthran) mammalian taxa show a similar flattening of the ulnar trochlear surface (e.g., myrmecophagids in Taylor, [77] and White,[78]; *Paramylodon* in Stock [79]). However, these taxa do not show flattening of the corresponding humeral trochlea, and have nothing like the apparent loss of flexion and extension seen in *P*. *azael*. Such ulnar (but not humeral) flattening is also expressed in the large bodied extinct vombatid *Phascolonus gigas* (S1D and S2D Figs). Murray [6] suggested this morphology may help stabilise and evenly distribute forces through the *Phascolonus* elbow under high loads experienced during digging or tearing with the manus. These patterns of forelimb engagement are also seen in myrmecophagids and (presumably) *Paramylodon*. That *P*. *azael* exhibits flattening not only of the ulna but also of the humerus may suggest it was especially committed to such forelimb uses. However, we propose that fossorial behaviour is unlikely in palorchestids (see discussion of the unguals below).

#### Postural differences

Could the flat humeroulnar joint have arisen to cope with a more medially-loaded elbow in the heaviest palorchestid? All palorchestid humeri appear to transmit weight medially over the ulna rather than centrally or laterally as in other vombatiforms. This is evidenced by the fact that in all palorchestid humeri, the vertical midshaft axis intersects the distal epiphysis through the trochlea, rather than through the approximate midline (as in *Phascolonus*, *Neohelos* and *Diprotodon)* or laterally through the capitulum (as in modern wombats and *Zygomaturus*) (S1 Fig). This loading regime appears to have persisted across the lineage, until in the largest species *P*. *azael* the fixation of the elbow may be a consequence of this imbalanced loading, exacerbated at heavier body masses. That *P*. *azael* loaded its elbow on the medial rather than lateral side could indicate a unique forelimb posture, perhaps somewhat sprawled with the elbows abducted from the sagittal plane. Fujiwara and Hutchinson [80] found sprawling tetrapods could be distinguished from other locomotory categories by their longer medial epicondyles, creating a greater elbow adductor moment arm in order to resist abducting ground reaction forces during sprawling gait. Such a posture is atypical for mammals, but the unusually elongate medial epicondyle in *P*. *azael*, along with their apparently medial loading regime and flattened humeroulnar articulation, may support postural reconstruction of *P*. *azael* with a sprawled forelimb.

#### Altered muscle actions on a fixed elbow

Immobilisation of the elbow significantly alters the primary actions of muscles crossing this joint. The key flexor *m*. *brachialis* becomes an important stabiliser, gaining leverage via distal positioning of its insertion on the ulnar tuberosity (Figs 5 and 6), and creating a deep spiral fossa and marked scars on the upper lateral and posterior humerus (Figs 3B, 3C, 4B and 4C). *M*. *epitrochleoanconeus*, a minor extensor in other marsupials, instead becomes hugely enlarged in its stabilising role, creating a diagnostic muscle scar on the posterior surface of the medial epicondyle and humeral shaft seen only in *P*. *azael* (Figs 3C and 4C).

In an immobile elbow, a biarticular muscle like *m*. *biceps brachii* becomes more significant in shoulder flexion and supination of the manus. Similarly, *m*. *triceps brachii*, rather than an active elbow extensor as suggested by the large olecranon [81], stabilises and maintains posture against the force of gravity when bearing weight on the forelimb. It would also perform powerful shoulder retraction as may be needed in raking and tearing with the manus when the elbow cannot be extended, evidenced by the huge infraglenoid origin for its scapular head (Figs 1 and 2).

#### Compensating for a fixed elbow

Compensation for lack of mobility in the elbow may instead have been available via humeral rotation, which would aid in positioning of the manus during locomotion and also manipulation of the environment when feeding. Without a more intact scapula such inferences are tentatively made, but many distinctive features on the *P*. *azael* humerus suggest powerful rotational movements of the shoulder. Firstly, the deltoid insertion is entirely separate and laterally displaced, increasing mechanical advantage for lateral rotation as noted in *Tamandua* by McAfee [82] (Fig 4A and 4B). Similarly, the elevation of the greater tubercle superior to the humeral head, while limiting ROM in abduction, would increase leverage in lateral rotation as well as stabilising the actions of the rotator cuff muscles (Fig 4A–4C). This morphology resembles that of extant wombats which perform powerful strokes with the shoulder when digging (but see discussion of the unguals below) (S1B and S1C Fig). Additionally, the insertion scar for *mm*. *latissimus dorsi* and *teres major* is very large and very distally positioned on the medial humerus, creating significant leverage for medial rotation (and/or resisting lateral rotation), as well as powerful retraction (Figs 4, 20 and 37). That this feature is common to all palorchestids speaks to the importance of these actions in their functional ecology. Finally, the pronounced medial cant peculiar to the *P*. *azael* pectoral crest may also reflect particularly strong medial rotation. The crest, freed of opposing deltoid muscle forces, is curled over the humerus by the pull of pectoral muscles medially rotating and adducting the shoulder (Fig 4A and 4D). Perhaps strong medial rotation and adduction of the shoulders allowed *P*. *azael* to exert a strong bilateral grip on the bole of a tree, grasping with the hands and retracting the shoulder to pull the upper body toward the tree to feed.

#### Plesiomorphic wrist and manus anatomy

The lack of known palorchestid carpals is frustrating; however, based on the available associated manual material from *P*. *parvus*, the group appear to have retained a fairly primitive plantigrade manus (Figs 25 and 26), resembling the *Ngapakaldia* condition. Distal forelimb and pedal similarities support this morphological affinity with *Ngapakaldia*, although the shortened, robust digit rays and thick metacarpals in *P*. *parvus* reflect increased weightbearing in the manus of these larger palorchestids.

The nature of this manual weightbearing is different to other large vombatiforms. Despite their apparently heavy bodies, large palorchestids were not graviportally adapted. The distal radius and ulna in *P*. *azael* and radius in *P*. *parvus* lack the dilated morphology apparent in *Zygomaturus* and *Diprotodon*, and contrast even with the wrists of the robust but smaller *Phascolonus* (S2 Fig). *Palorchestes* species retain a *Ngapakaldia-*like, trapezoidal distal radius and gracile, hooked styloid process. It seems that selective pressure for a relatively unmodified, dextrous manus in *Palorchestes* precluded the weight-related changes seen in graviportal taxa like *Zygomaturus* and *Diprotodon*.

#### Enlarged claws

The other remarkable manual morphology characteristic of palorchestids are their deep, laterally-compressed, knife-like, penetrating ungual phalanges (Figs 9, 10, 25, 26, 34, 35 and 40). These would have been encased within keratinous claw sheaths that extended the length and curvature of the bone contour by a great deal–around 30% based on koalas but as much as ~80% based on large myrmecophagids and ursids (pers. obs.). Being dorsoventrally deep they appear adapted for high stresses such as those experienced during climbing or digging [83]. However, an arboreal habit appears extremely unlikely, both due to their now-apparent large body size and lack of important substantiating morphological evidence such as palmar tuberosities on the proximal phalanges indicative of the strong habitual grasping characteristic of arboreal vombatiforms like *Nimbadon* and *Phascolarctos* [20]. Likewise, the lateral compression and sharp distal ends of the claws make them poorly shaped for digging. Indeed, Szalay [71] notes that palorchestid claws are ‘almost at the opposite end of terminal phalanx construction’ to fossorial mammals. This indicates that palorchestids interacted very differently with the substrate than their contemporary diprotodontid sister taxa, in which the ancestral laterally-compressed claw shape became reduced in favour of weightbearing in larger species [11]. Despite attaining Pleistocene body masses comparable to *Zygomaturus*, palorchestids retained this ancestral autopod with claws apparently adapted not for arboreal or fossorial uses, but for slicing, clinging and raking.

#### Powerful movements of the wrist and digits

*P*. *parvus* has short, sturdy digits which when extended could be partially abducted at the domed metacarpophalangeal joints to splay the fingers and increase the spread of the manus (Figs 25 and 26). The metacarpal heads are strongly trochleated on the palmar side, more so even than in extant ursids, which would stabilise the digits when flexed and resist lateral deviations when under high loads experienced during raking actions (Fig 25A–25H). The proximal interphalangeal joints are restricted to dorsoventral movements by their keeled articulations, although dorsal bony stops at each joint prevent hyperextension. The distal interphalangeal joints, while clearly mobile, certainly do not permit the degree of ungual hyperextension seen in felids or viverrids. This eliminates the possibility of ‘retractable claws’ suggested by Flannery and Archer [10], while their other propositions of knuckle-walking or manolateral hand postures are not supported by any morphology of the palorchestid wrist and hand currently known.

Use of the manus in powerful clinging and tearing motions is further supported by the hugely broad distal humerus characteristic of palorchestids (Figs 3, 4, 19, 20, 36 and 37), providing both large muscle attachment area and increased leverage for powerful flexors and extensors of the wrist and digits. Flexors from the medial epicondyle would provide the power to grip and embed the claws into the substrate, while strong extensors from the lateral epicondyle would assist in tearing and pulling that substrate apart. Such enhanced forearm strength and large claws are again strongly suggestive of adaptation of the limb to use during feeding. Coombs [11] noted that few large mammals today use their clawed forelimbs to browse, exceptions being some bear species, and highlighted that appropriate extant analogues for such behaviour are few. Future comparative analyses may help to resolve the appendicular biomechanics of feeding in the palorchestid forelimb and will be important in understanding the overall dietary adaptations of the group.

### Hindlimb adaptations

#### Narrow, adducted hips

Large diprotodontids are conventionally reconstructed with a wide-set stance, columnar hindlimb posture and slightly bowed knees, owing to the obtuse angle between their long femoral neck and adjoining shaft, the unequal form of the femoral condyles (S3 Fig), and the morphology of the tibia and talocrural joint [84]. Fossil trackways have supported such reconstructions, and have even been suggested to depict sexual dimorphism in gait width due to large and cumbersome pouch young carried by the female [85]. Palorchestid morphology described here indicates a more gracile hindlimb in a very different posture, with hips adducted and more extended knees.

The *Palorchestes* femur differs strongly from diprotodontids of similar size and suggests habitual postural differences. With a more circular diaphyseal section profile and very short neck aligning the femoral head approximately vertically level with the medial epicondyle, this femoral morphology resembles *Phascolonus* (S3 Fig). In fact, the specimen figured (Figs 13 and 14) was long referred to that genus. However, in proportions the *Palorchestes* femur is much more elongate than *Phascolonus* and differs markedly in its distal morphology (S3D Fig). In *Palorchestes* the femoral condyles lie approximately level in anterior view (Figs 13A and 31A) and the medial patellar surface does not protrude anteriorly (Figs 13F and 30I), contrasting with *Phascolonus* and other large-bodied vombatiforms *Zygomaturus* and *Diprotodon*. In these taxa the lateral condyle is also distally offset, reflecting a more abducted hip posture. This posture requires marked anterior projection of the medial patellar surface to resist medial tracking of the patelloid due to the more acute angle between the iliac origin of the quadriceps its insertion on the tibial tuberosity. *Palorchestes* femora lack this morphology and instead these animals appear to have held their femur and tibia more vertically in the dorsal plane.

The os coxae are slender, and when rearticulated using the symphyseal epiphysis produce a relatively narrower width across the ilia than in diprotodontid or vombatid pelves (Figs 11 and 12). This indicates a leaner abdominal girth in *Palorchestes* compared to other browsing vombatiforms, which may relate to dietary differences. Coprolites or fossilised gut contents could potentially be used to test this idea, however such specimens from any marsupial megafauna, let alone *Palorchestes*, are extremely rare [86, 87]. So, the question of whether this pelvic morphology is actually related to dietary ecology awaits further evidence.

#### Extended hindlimb posture

The limited height of the posterior femoral condylar surface belies the morphology seen in species that bear weight on a habitually flexed knee [82, 88] and may instead indicate a more extended posture (Figs 13C, 14C, 30C and 31C). Further to this are the tall, quadrangular intercondylar eminence on the proximal tibia (locking into the intercondylar fossa on the distal femur and stabilising an extended knee, Figs 15C, 16C, 32C and 33C), and the relatively proximal origin for the lateral head of *m*. *gastrocnemius* on the femur. In *P*. *azael* this origin is more proximal than any other vombatiform taxon studied, suggesting a significant role of this muscle in stabilising the extended knee. Its lateral head would act to increase lateral knee joint congruence, contracting to decrease the distance between lateral condyle and articulating tibial surface, and preventing leg adduction/*genu varus*. Additionally, the reduced and very proximal position of the tibial crest as an insertion for the *m*. *biceps femoris* on the lateral tibia indicates a shorter moment arm and lower leverage of this muscle across the knee, further evidence of habitually extended knee posture. These features point toward an adducted, extended hindlimb posture in *Palorchestes*, contrasting with the wider-set stance of diprotodontids and crouched posture in vombatids. Such postural differences would suggest palorchestids required less hindlimb muscle bulk than equivalently-sized diprotodontids, as more weight could be borne directly via the skeleton with the hindlimbs adducted under the body, without the extensive muscular support needed to sustain a wider-set diprotodontid-like posture.

A further peculiarity in the *Palorchestes* hindlimb is the vertical, laterally-facing and flat fibular facet on the proximal tibia in *P*. *parvus* (Fig 33B and 33C), suggesting reduced loads on the fibula than in diprotodontids or vombatids, and possibly increased tibiofibular mobility. With damage to this area in the *P*. *azael* specimen, and lacking any fibula or complete astragalus specimens, it is difficult to draw conclusions; however, this preserved morphology in two *P*. *parvus* specimens may hint at retention of a more primitive, mobile tibiofibular joint once important in an ancestrally arboreal lifestyle.

#### Plantigrade, syndactylous pes

Well-developed plantar tuberosities on the navicular (Figs 17F and 34K) and ectocuneiform (Figs 17D and 34L) in *Palorchestes parvus* indicate a plantigrade foot posture. Additionally, the syndactylous second and third digits are not hugely different from the plesiomorphic possum-like condition from which all diprotodontian feet evolved [71] (Figs 34A and 35). The combination of large body mass with the constraint of a syndactylous pes necessitated inflation of the fourth and fifth digits to bear the laterally-distributed weight. This is a less distorted pes than in the strange rotated toes in the giant *Diprotodon* [84], nor is it a true ‘pedolateral’ pes as seen in some extinct ground sloths [89], as the weight is not borne on the lateral surface of the metatarsals. Instead these thick, short, clawed lateral digits resemble a hyper-robust *Ngapakaldia* [24] and bore weight on their true plantar surface.

With additional associated tarsals for *P*. *azael*, including the cuboid and calcaneus, it is clear they too had a plantigrade pes (Figs 17 and 18). Comparing the metatarsal facets on the ectocuneiform between *P*. *parvus* and *P*. *azael* (Figs 17D and 34L), it appears digits 2 and 3 may have been less reduced in the latter, instead being slightly more equal in size to their neighbouring digits as in *Zygomaturus* and *Phascolonus*. This shift toward higher digital uniformity in *Palorchestes* could be related to large body size, where *P*. *azael* is above a threshold beyond which the ancestral disparity in digit proportions is maladaptive and therefore lost.

It is interesting to note that throughout these descriptions of the hindlimb, palorchestid morphology frequently compares most closely to *Ngapakaldia*, being mostly much larger but often similar in form. *Ngapakaldia* was originally described as a primitive palorchestid and this taxonomic status remained for many years [24, 90, 91]. Eventually the genus was shifted to Diprotodontinae after comprehensive craniodental phylogenetic analysis by Black [7]. These historical ideas about *Ngapakaldia* as a primitive palorchestid, or similar to a hypothetical common ancestor with mosaic features from zygomaturines, palorchestids and diprotodontines [92], are borne out by similarities in appendicular morphology even to the most derived *Palorchestes* species.

#### Facultative bipedality?

In order to employ the forelimb in the manner suggested by their anatomy, palorchestids would need to rear into a bipedal stance to free the manus from weightbearing while in use. However, few aspects of the hindlimb in *P*. *azael* point towards specific adaptation to bipedal posture, and several features appear to hinder it. For example, the hindlimb overall is less robust than similarly-sized diprotodontids. The acetabulum appears surprisingly shallow, reducing stability of the hip. In the femur, a short femoral neck means that the greater trochanter projects above the head, limiting abduction and rotation and restricting the hip to movements largely in the parasagittal plane. This contrasts with the proximal femoral shape of more adept facultative bipeds such as ursids [93]. Indeed, the bear-like elevated femoral head in the Pleistocene zygomaturine *Hulitherium* was cited as evidence for a facultatively bipedal stance in that species [94].

However, given their apparent forelimb strength and penetrating claws it seems likely that palorchestids could have pulled their body upright into a bipedal position while supported against a tree by the forelimbs, with the manual claws hooked into the bark. This would allow the elongate face and protrusible tongue to access additional, higher browse than could be reached in a quadrupedal position. Similarly, palorchestids may have adopted such a bipedal posture to topple ferns or cycads by throwing their bodyweight against them with the forelimbs, making young fiddlehead shoots or fleshy seeds up at the crown accessible at ground level. This possibility is further supported by the long, dorsally rotated ischium in *P*. *azael*, a character interpreted in other mammals as adaptive for upright trunk posture through maintaining the moment arm of the hamstrings when the femur is extended [95, 96]. Such morphology would allow stronger hip extension when standing erect in this scenario, perhaps to brace the hindlimb while pushing with the forelimb.

It is also possible that the relative gracility of the palorchestid hindlimb is evidence not of them being facultatively bipedal, but *tripodal–*that is, providing additional support for the rearing body with a muscular tail, behaviour well-known in macropodids [88, 97] and recently reasoned in the extinct vombatiform *Thylacoleo* [98]. *Palorchestes* has previously been noted to possess a ‘kangaroo-like tail’ [47, 99] larger than that of their diprotodontid kin, potentially as an adaptation to tripodal posture [6], though this has never been fully investigated. Axial elements including the caudal vertebrae were not considered in the current appendicular descriptions but may in future provide valuable insight into any palorchestid tripodal adaptation and potential convergence on such a ‘ground sloth’-like habit.

## Conclusions

This work represents the first quantitative body mass estimates and descriptions of appendicular morphology in the Palorchestidae, collating over 60 specimens across three taxa to finally provide a comprehensive view of their unique anatomy. The postcranial evidence presented here reinforces existing knowledge of the extraordinary palorchestid craniodental morphology to cement their status as one of the strangest marsupial lineages ever to have existed. Our findings certainly support Flannery and Archer [10] in their notion that palorchestids were adapted for a niche no longer occupied in modern Australian landscapes.

We are beginning to build a picture of the palorchestids as they were in life: Giant plantigrade quadrupeds with a slender body form, muscular bent forelimbs, straighter hindlimbs and enlarged claws. These features evoke a specialised feeder actively using its forelimbs to acquire browse, and with restricted elbow mobility in larger species such actions must have been driven by a powerful shoulder, perhaps in a somewhat abducted posture. They may have adopted a bipedal stance to feed, but further details of their locomotor adaptations such as carriage of the manus remain unresolved. However, it is clear that palorchestids moved and behaved in a way vastly different to their contemporaneous diprotodontid kin despite attaining comparable body sizes.

The paucity of known palorchestid postcranial fossils makes more exhaustive functional and evolutionary interpretation of the appendicular skeleton challenging, especially the lack of scapula, fibula and carpals. It is hoped that further unidentified material held in collections may become referable to the family, genus or species level as a result of the present work. Future studies on the currently known material will attempt to quantify the morphological differences described here in a broader mammalian context, and test hypotheses of limb function using geometric morphometric and musculoskeletal modelling methods.

## Supporting information

S1 Table Measurements and museum collection registration details for appendicular material of palorchestids and comparative taxa included in this study. (XLSX)

S2 Table Body size calculations for comparative vombatiform taxa included in this study, with dataset used to generate predictive equations. (XLSX)

S1 Fig Illustrations of humeri from comparative vombatiform taxa used in this study. All elements scaled to the same length to emphasise overall shape and proportion differences. (A) *Phascolarctos;* (B) *Vombatus;* (C) *Lasiorhinus;* (D) *Phascolonus;* (E) *Nimbadon;* (F) *Neohelos;* (G) *Zygomaturus;* (H) *Thylacoleo;* (I) *Ngapakaldia;* (J) *Diprotodon*; (K) *Propalorchestes;* (L) *Palorchestes parvus;* (M) *Palorchestes azael*. Scale bar 50 mm.
(TIF)

S2 Fig Illustrations of ulnae from comparative vombatiform taxa used in this study. All elements scaled to the same length to emphasise overall shape and proportion differences. Each pair shows anterior view on the left, medial view on the right. (A) *Phascolarctos;* (B) *Vombatus;* (C) *Lasiorhinus;* (D) *Phascolonus;* (E) *Nimbadon;* (F) *Neohelos;* (G) *Zygomaturus;* (H) *Thylacoleo;* (I) *Ngapakaldia;* (J) *Diprotodon*; (K) *Propalorchestes;* (L) *Palorchestes azael*. Scale bar 50 mm.
(TIF)

S3 Fig Illustrations of femora from comparative vombatiform taxa used in this study. All elements scaled to the same length to emphasise overall shape and proportion differences. Each pair shows anterior view on the left, posterior view on the right. (A) *Phascolarctos;* (B) *Vombatus;* (C) *Lasiorhinus;* (D) *Phascolonus;* (E) *Nimbadon;* (F) *Neohelos;* (G) *Zygomaturus;* (H) *Thylacoleo;* (I) *Ngapakaldia;* (J) *Diprotodon*; (K) *Palorchestes parvus;* (L) *Palorchestes azael*. Scale bar 50 mm.
(TIF)

S4 Fig Cladogram of Order Diprotodontia showing the relative position of Vombatiformes. (PDF)

## Abbreviations

AM F: Australian Museum fossil collection
FU: Flinders University collection
NHMUK: Natural History Museum, London
MZ: Monash University zoology collection
NMV C: Museums Victoria mammal collection
NMV P: Museums Victoria Palaeontology Collection
NTM: Museum and Art Gallery of the Northern Territory
SAMA P: South Australian Museum palaeontology collection
