# Supplementary figures and images for: The extraordinary osteology and functional morphology of the limbs in Palorchestidae, a family of strange extinct marsupial giants
Source: PLoS One. 2019 Sep 13;14(9):e0221824. doi: 10.1371/journal.pone.0221824 (PMC6744111; doi:10.1371/journal.pone.0221824)

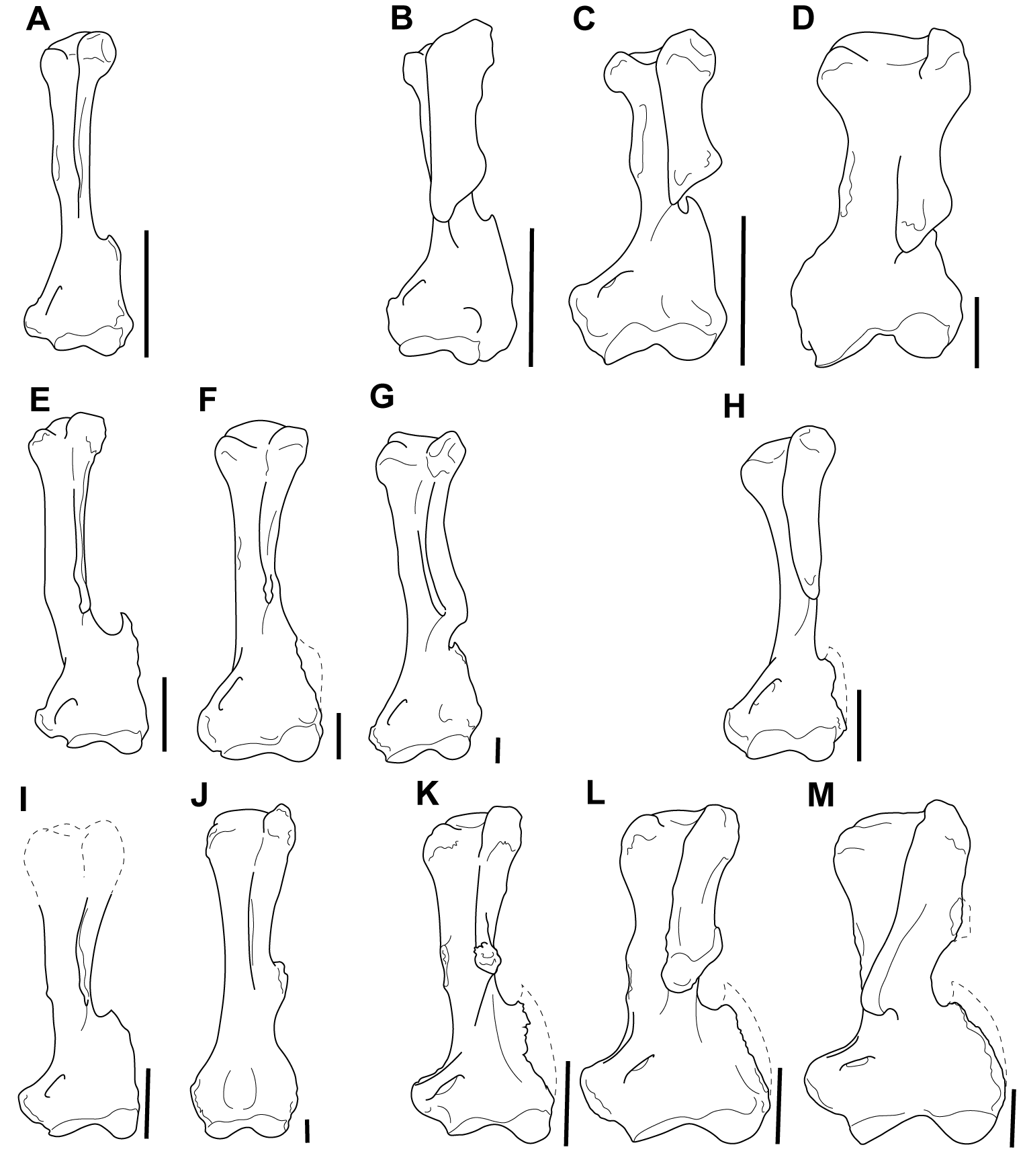

Supplement: S1 Fig — All elements scaled to the same length to emphasise overall shape and proportion differences. (A) Phascolarctos; (B) Vombatus; (C) Lasiorhinus; (D) Phascolonus; (E) Nimbadon; (F) Neohelos; (G) Zygomaturus; (H) Thylacoleo; (I) Ngapakaldia; (J) Diprotodon; (K) Propalorchestes; (L) Palorchestes parvus; (M) Palorchestes azael. Scale bar 50 mm. (TIF) [file pone.0221824.s003.tif]

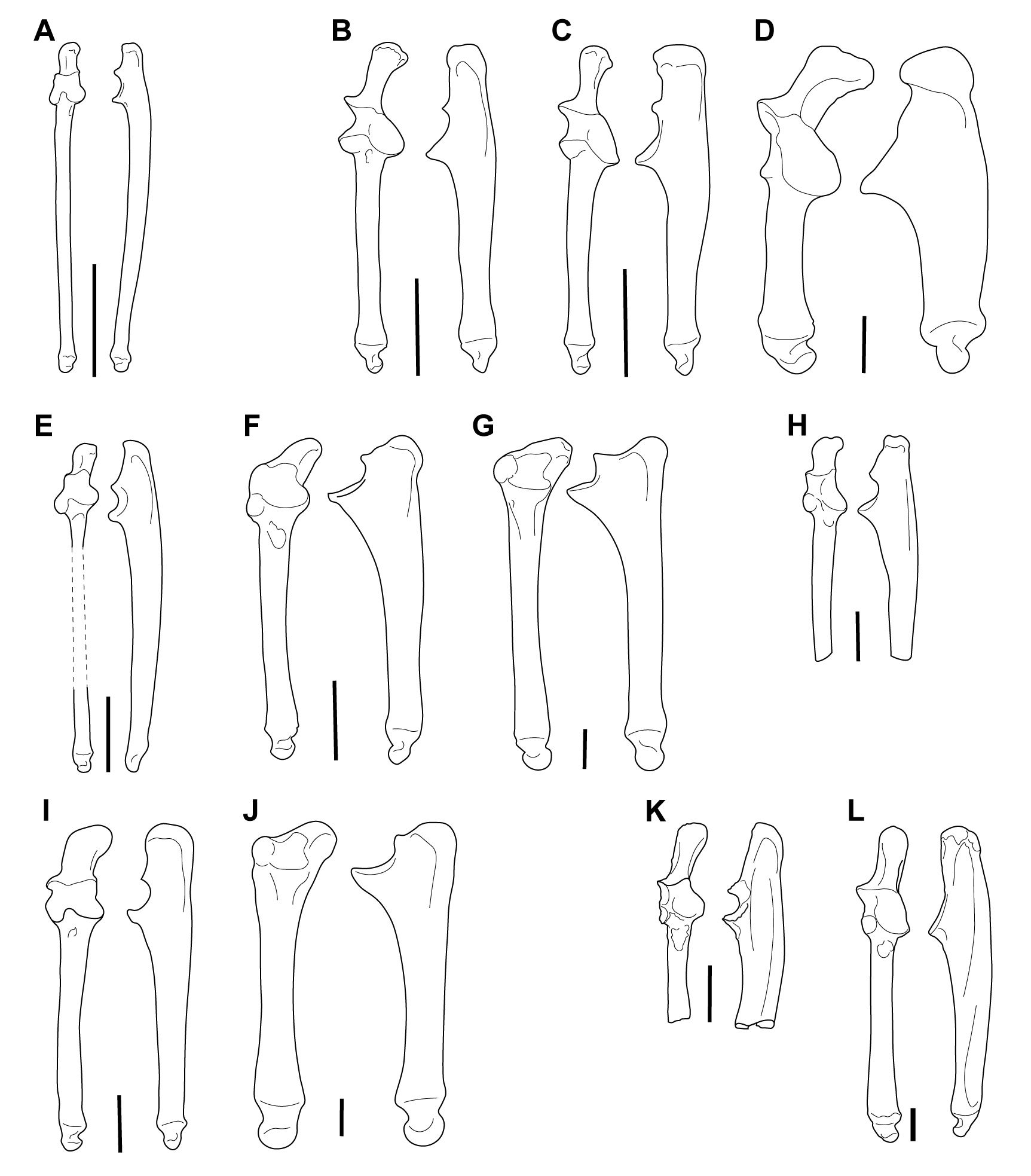

Supplement: S2 Fig — All elements scaled to the same length to emphasise overall shape and proportion differences. Each pair shows anterior view on the left, medial view on the right. (A) Phascolarctos; (B) Vombatus; (C) Lasiorhinus; (D) Phascolonus; (E) Nimbadon; (F) Neohelos; (G) Zygomaturus; (H) Thylacoleo; (I) Ngapakaldia; (J) Diprotodon; (K) Propalorchestes; (L) Palorchestes azael. Scale bar 50 mm. (TIF) [file pone.0221824.s004.tif]

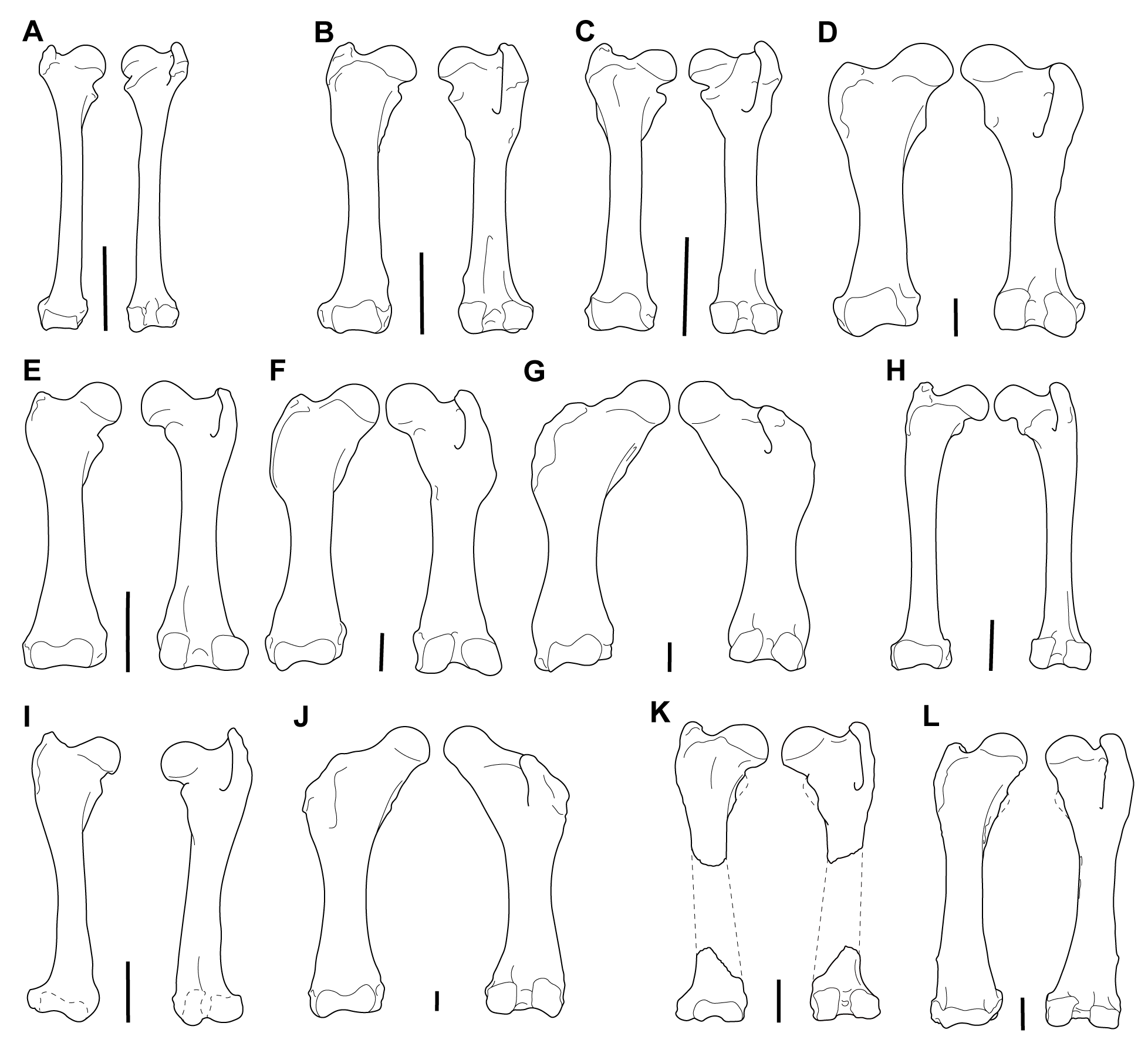

Supplement: S3 Fig — All elements scaled to the same length to emphasise overall shape and proportion differences. Each pair shows anterior view on the left, posterior view on the right. (A) Phascolarctos; (B) Vombatus; (C) Lasiorhinus; (D) Phascolonus; (E) Nimbadon; (F) Neohelos; (G) Zygomaturus; (H) Thylacoleo; (I) Ngapakaldia; (J) Diprotodon; (K) Palorchestes parvus; (L) Palorchestes azael. Scale bar 50 mm. (TIF) [file pone.0221824.s005.tif]
