# Supplementary material for: The extraordinary osteology and functional morphology of the limbs in Palorchestidae, a family of strange extinct marsupial giants
Source: PLoS One. 2019 Sep 13;14(9):e0221824. doi: 10.1371/journal.pone.0221824 (PMC6744111; doi:10.1371/journal.pone.0221824)
Supplement: S4 Fig — (PDF) [file pone.0221824.s006.pdf]

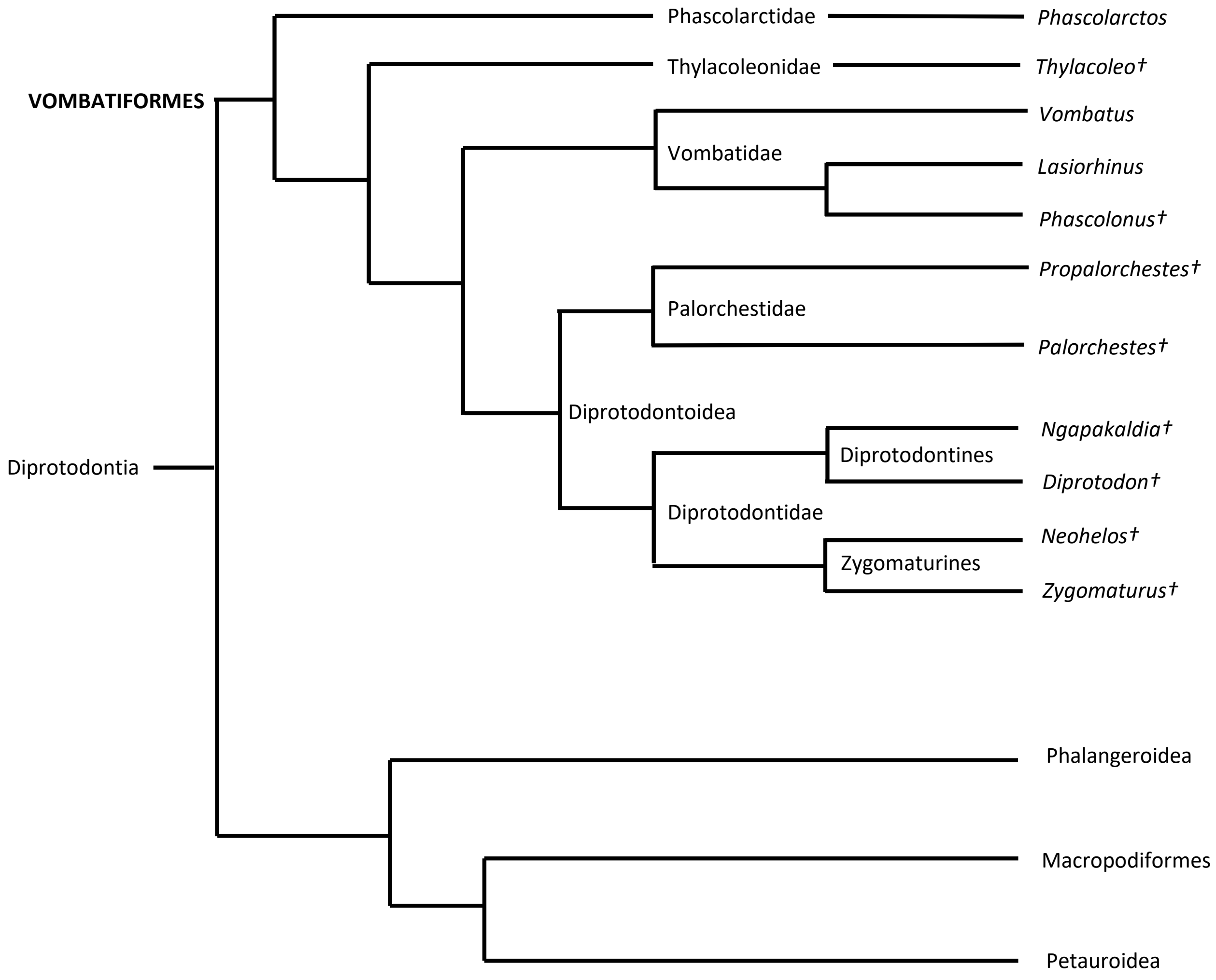

Simplified tree modified from from Murray 1998, Black et al. 2012, Mitchell et al. 2014

1. Murray P. Palaeontology and palaeobiology of wombats. In: Wells RT, Pridmore PA, editors. Wombats. Adelaide, South Australia: Surrey, Beatty and Sons; 1998. p. 1-33.
2. Black KH, Archer M, Hand SJ, Godthelp H. The rise of Australian marsupials: A synopsis of biostratigraphic, phylogenetic, palaeoecologic and palaeobiogeographic understanding. In: Talent JA, editor. Earth and Life. Netherlands: Springer; 2012. p. 983-1078.
3. Mitchell KJ, Pratt RC, Watson LN, Gibb GC, Llamas B, Kasper M, et al. Molecular Phylogeny, Biogeography, and Habitat Preference Evolution of Marsupials. Molecular Biology and Evolution. 2014;31(9):2322-30.
